# Double-Bridging Increases the Stability of Zinc(II) Metal–Organic Cages

**DOI:** 10.1021/jacs.4c09742

**Published:** 2024-11-04

**Authors:** Hannah Kurz, Paula C. P. Teeuwen, Tanya K. Ronson, Jack B. Hoffman, Philipp Pracht, David J. Wales, Jonathan R. Nitschke

**Affiliations:** Yusuf Hamied Department of Chemistry, University of Cambridge, Lensfield Road, Cambridge CB2 1EW, U.K.

## Abstract

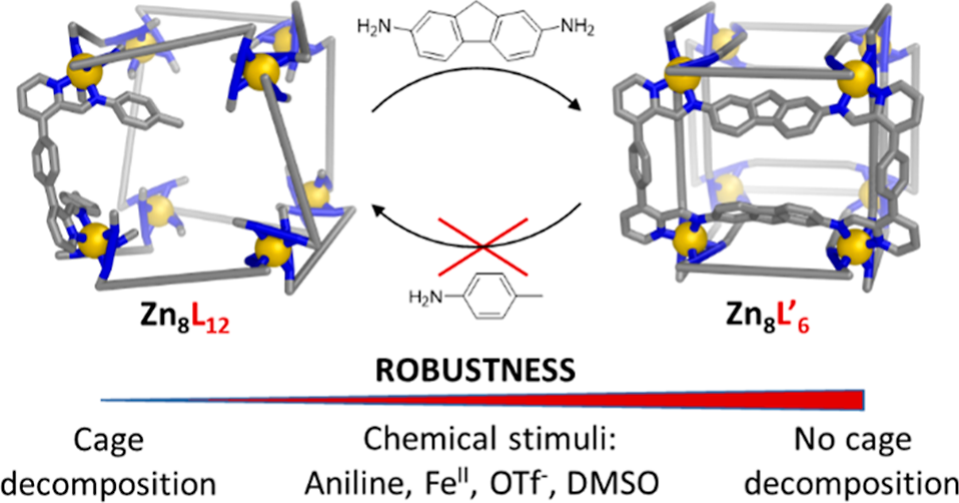

A key feature of coordination cages is the dynamic nature of their coordinative bonds, which facilitates the synthesis of complex polyhedral structures and their post-assembly modification. However, this dynamic nature can limit cage stability. Increasing cage robustness is important for real-world use cases. Here we introduce a double-bridging strategy to increase cage stability, where designed pairs of bifunctional subcomponents combine to generate rectangular tetratopic ligands within pseudo-cubic Zn_8_L_6_ cages. These cages withstand transmetalation, the addition of competing ligands, and nucleophilic imines, under conditions where their single-bridged congeners decompose. Our approach not only increases the stability and robustness of the cages while maintaining their polyhedral structure, but also enables the incorporation of additional functional units in proximity to the cavity. The double-bridging strategy also facilitates the synthesis of larger cages, which are inaccessible as single-bridged congeners.

## Introduction

Molecular capsules have proved useful in applications that include separation and purification,^1,2^ sensing,^3,4^ storage of reactive compounds,^5−7^ and (photo)catalysis.^8−12^ The functionality of these capsules in solution complements the solid-state applications of porous networks, such as metal–organic frameworks.^13,14^ Subcomponent self-assembly, where ligands and capsule assemble from smaller subunits during the same overall process, has enabled the rapid preparation of structurally complex polyhedral capsules with little synthetic effort.^15−19^ Such capsules can be tailored through subcomponent exchange or other post-assembly modifications.^20−24^ The dynamic nature of the coordination bonds that hold these structures together provides advantages in their synthesis and applications, but these dynamic linkages also limit capsule stability. For example, competing ligands can degrade cages by extracting their metal ions. The labile imine bonds formed within ligands during subcomponent self-assembly are also susceptible to acid- or base-catalyzed hydrolysis.^25^ This property can limit the application potential of coordination cages in real-world scenarios, which can require low cage concentrations or high stability in complex solutions with potentially interfering species, such as ligands and metal ions.

One approach to increasing cage stability is to use metal–ligand combinations with intrinsically stable coordination bonds. Metals in high oxidation states, such as Zr^IV^ or Co^III^, or 4d and especially 5d metals such as Pt^II^, thus generate more stable cages.^26−30^ However, the slow dynamic exchange of coordination bonds can impede cage formation, as less stable structures become kinetically trapped, requiring strategies such as post-assembly oxidation, dynamic covalent approach, or harsh reaction conditions.^31−35^ Most strategies for stabilizing coordination bonds are based on matching the softness/hardness of the metal and ligand donor,^36^ optimization of the coordination geometry,^37,38^ and enhancing the Lewis acidity/basicity of the metal ion/ligand.^39,40^ These strategies can also provide control over dynamic ligand or subcomponent exchange, which is often based on the introduction of labile bonds such as imines.^20,25,40−47^ Another well-investigated approach to stabilize coordination cages is based on strategic entropy gain, for example by using high-topicity ligands, leading to preorganization.^48−50^ For instance, capping cages increases the density of connections that bind them together, increasing their stability.^51−54^

In this work, we demonstrate the stabilization of pyridyl-imine-based Zn_8_L_6_ coordination cages through double-bridging their edges. These cages were constructed from a dialdehyde subcomponent that provides a novel vertex coordination geometry upon imine condensation. This arrangement contrasts with other pyridyl-imine-based cages^55−57^ because of the unique orientations of the aniline units within the product structure. This geometry positions the aniline residues of these imines so that cross-linking between ligands is possible when certain dianilines are added. The resulting double-bridged ligands thus chelate four zinc ions instead of two—their topicity increases from 2 to 4 going from the cubic to the pseudo-cubic framework. This increase in topicity increases the robustness of the cage, enhancing its application potential. The double-bridging approach also enabled the assembly of a larger pseudo-cubic cage, which could not be obtained as the single-bridged version.

## Results and Discussion

### Self-Assembly and Characterization of Single- and Double-Bridged Cages

**Figure 1:**
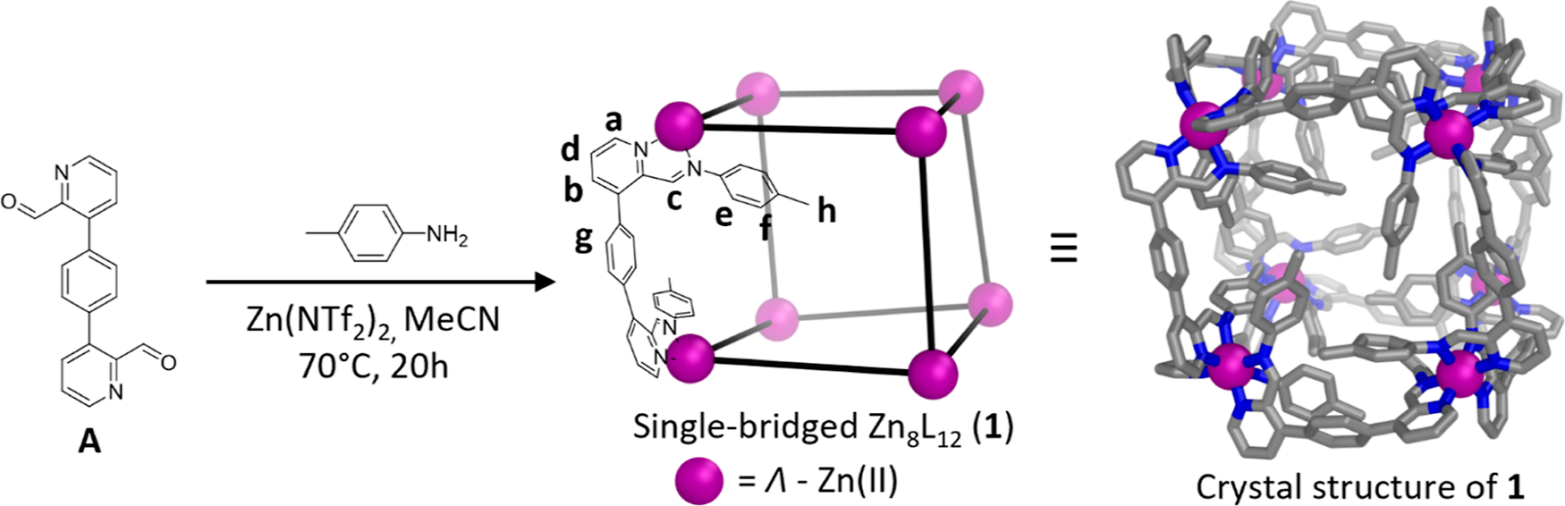
Subcomponent self-assembly of **A**, *p*-toluidine, and Zn(NTf_2_)_2_, to form *O*-symmetric Zn_8_L_12_ cube **1**. Protons are assigned according to 1D and 2D NMR spectra (Figures S11–S16). Only the Λ_8_ enantiomer is shown. Hydrogen atoms and counteranions are omitted for clarity.

Subcomponent **A** (Figure 1) was synthesized via Pd-catalyzed Suzuki–Miyaura cross-coupling as detailed in the Supporting Information (Figures S1–S3, pages 2–4 in the SI). Heating subcomponent **A** (1.5 equiv), *p*-toluidine (3.0 equiv), and zinc(II) bis(trifluoromethylsulfonyl)imide (Zn(NTf_2_)_2_, 1 equiv) in acetonitrile gave cubic [Zn_8_L_12_]^16+^ cage **1**. The ^1^H NMR spectrum of **1** was consistent with a highly symmetrical species, as only one set of ligand signals was obtained (Figures S11, S12). Further NMR experiments (Figures S13–S16) enabled the assignment of the peaks as shown in Figures 1 and S11. While the pyridyl, imine and methyl protons gave sharp peaks in the ^1^H NMR spectrum, the signals of the phenylene protons e, f and g were broad and overlapping. Upon increasing the temperature, the signals become sharper, consistent with restricted rotational motion of the phenylene groups at room temperature (Figure S17). The nuclear overhauser effect spectroscopy (NOESY) NMR spectrum of **1** indicated through-space interactions between protons h, e and c, confirming that the tolyl groups point toward each other on the faces of the cube (Figures S18, S19). This arrangement contrasts with other pyridyl-imine-based cages, whose vertices bear externally oriented substituents (Figure S20).^15,56,57^

Crystals of **1** suitable for single-crystal X-ray diffraction were obtained by vapor diffusion of benzene into an acetonitrile solution of **1** containing excess KSbF_6_ (see Figures 1 and S71, with details given on SI pages 43–45). As shown in Figure 1, the crystal structure obtained was consistent with the solution-state configuration of the tolyl substituents observed by NOESY, with each tolyl group at 90° to its nearest neighbors on the respective cage face. Cage **1** thus exhibits *O* point symmetry, with all metal ions within a cage exhibiting the same Λ- or Δ-handedness, and both cage enantiomers present in the crystal.

Diffusion ordered spectroscopy (DOSY) NMR spectra verified the formation of a single species with a solvodynamic radius of 18.4 Å (Figure S21). The radius exceeds the value of 14.1 Å based upon the crystal structure (Figure S71). As the experimental radius remained unchanged upon concentration increase from 0.2 to 0.8 mM, we infer that aggregation is not responsible for the larger-than-expected radius observed in solution (Figure S22). The increased solvodynamic radius might be a consequence of interactions between **1** and counteranions or solvent molecules. In contrast to previously reported pyridyl-imine cages, the charged metal centers of **1** are more exposed to the external environment, with less shielding by the ligands.

In the ^19^F NMR spectrum of **1**, two signals at −78.6 and −80.4 ppm were observed, consistent with the binding of a Tf_2_N^–^ anion in slow exchange on the NMR time scale (Figure S23). Heteronuclear Overhauser enhancement spectroscopy (HOESY) experiments confirmed ^1^H–^19^F through-space interactions between the encapsulated Tf_2_N^–^ and the tolyl substituents at the faces of cage **1** (Figure S24). We were not able to prepare **1** from Zn(OTf)_2_ or Zn(BF_4_)_2_ under similar reaction conditions, suggesting that the encapsulated Tf_2_N^–^ anion exercised a stabilizing effect. CH–π interactions between neighboring *p*-tolyl groups may also serve to stabilize the framework of **1**, as we were unable to synthesize cage **1** with aniline in place of *p*-toluidine under the same reaction conditions.

High resolution electrospray ionization mass spectrometry (ESI-MS) confirmed the presence of cubic cage **1** in solution (Figures S25, S26). However, cage fragmentation could only be prevented by applying very mild MS conditions, consistent with low stability of **1** in the mass spectrometer. Even under mild MS conditions, many ions attributable to fragments of **1** were observed (Figure S25).

**Figure 2:**
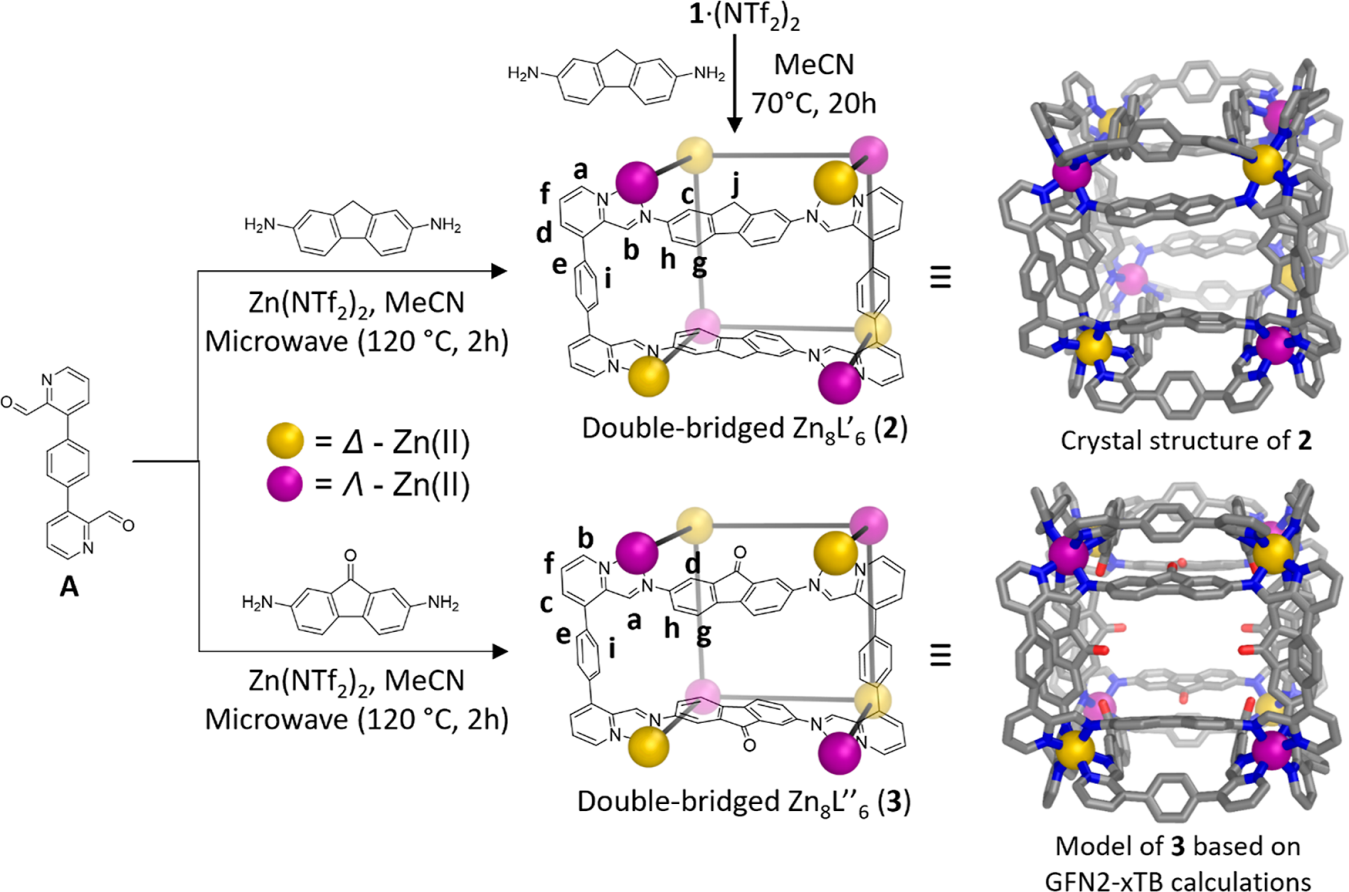
Self-assembly of double-bridged zinc(II) cages based on subcomponent **A**. (Top) Self-assembly of subcomponents **A**, 2,7-diaminofluorene, and Zn(NTf_2_)_2_, forming achiral double-bridged Zn_8_L′_6_ cage **2** (illustrated as a schematic model and the crystal structure of *T*_h_-symmetric **2**•(PF_6_)_16_). Cage **2** was also formed through reaction of **1** with 2,7-diaminofluorene. (Bottom) Self-assembly of subcomponents **A** and 2,7-diaminofluorenone with Zn(NTf_2_)_2_, forming achiral double-bridged Zn_8_L″_6_**3** (illustrated as a schematic model and a model based on GFN2-xTB calculations). Proton assignments are derived from NMR spectra (Figures S27−S33, S44−S48). Hydrogen atoms and counteranions are omitted for clarity.

The novel vertex motif of **1**, which orients its tolyl substituents parallel to the cage faces, suggested the possibility of double-bridging the cage faces by linking them with dianilines. The reaction of subcomponent **A** (1.5 equiv) and 2,7-diaminofluorene (1.5 equiv) with Zn(NTf_2_)_2_ (1.1 equiv) in acetonitrile in a microwave reactor produced double-bridged [Zn_8_L′_6_]^16+^ pseudo-cube **2** (Figure 2 top). NMR spectra of **2** confirmed the formation of a single high-symmetry species and enabled assignment of its ^1^H NMR spectrum (Figures S27–S33). HR-ESI-MS (Figures S41–S43) measurements confirmed its composition.

The double-bridging in cage **2** fundamentally alters its stereochemistry. In contrast to singly bridged **1**, the extra ligand bridges in **2** enforce a 1:1 mixture of Δ and Λ handed metal centers, in which every zinc(II) center has nearest neighbors of the opposite handedness, as shown in Figure 2. Single crystals of **2** suitable for X-ray diffraction analysis were obtained by vapor diffusion of diethyl ether into an acetonitrile solution of **2** containing excess TBA^+^PF_6_^–^ (see Figures 2 and S72, with details given on SI pages 43–46). The crystal structure confirmed the alternating Λ- and Δ-handedness of the metal centers, resulting in achiral *T*_h_ symmetry.

In the NOESY NMR spectrum of **2**, a through-space interaction was observed between the protons b and h, but not between c and h (Figures S34, S35). These observations confirm the locked state of the fluorene unit, whose bent geometry prevents free rotation. Furthermore, CH–π interactions between the fluorene unit and the phenyl unit prevent free rotation of the phenyl unit and result in splitting of the aromatic protons e and i. As proton i is pointing toward the fluorene unit, it is strongly shielded by its ring current, leading to a pronounced upfield shift with Δδ = 5 ppm. Upon temperature increase, the increased rotational freedom results in broadening of the two proton signals e and i and shifts them closer to each other (Figure S36). Furthermore, even upon temperature increase to 368 K in deuterated nitromethane solution, convergence into a single sharp signal was not observed (Figure S37). These observations indicate that the solution structure of **2** is isomorphous to the crystal structure.

DOSY NMR gave results consistent with the presence of a single species with a solvodynamic radius of 19.7 Å (Figure S38). As with **1**, the solvodynamic radius was concentration-independent and exceeds the radius of 14.1 Å determined from the crystal structure (Figures S39, S72). The solution and solid-state radii of **1** and **2** are nearly identical, confirming that double-bridging impacted the cage framework symmetry, but not its size. In contrast to **1**, only one Tf_2_N^–^ signal, which is slightly shifted compared to a TBA^+^Tf_2_N^–^ control sample, was observed in the ^19^F NMR spectrum of **2** (Figure S40), indicating fast exchange of this anion between bulk solution and the cage cavity.

As the fluorene CH_2_ protons point toward the cage window and cavity, we inferred that alteration at the CH_2_ position of the fluorene unit might enable endohedral functionalization, thus changing the size and properties of the cage cavity and windows. We therefore prepared the derivative 2,7-diamino-9-fluorenone (Figures S4, S5, pages 4, 5 in the SI), and combined it with subcomponent **A** and Zn(NTf_2_)_2_ in acetonitrile in a microwave reactor (Figure 2 bottom). The formation of double-bridged [Zn_8_L″_6_]^16+^ pseudo-cube **3** was confirmed by NMR and ESI-MS experiments (Figures S44–S56).

We were not able to obtain crystals of **3** suitable for single-crystal X-ray diffraction, despite numerous attempts. We therefore optimized its structure using GFN2-xTB calculations, which produced a geometry very similar to the X-ray structure of cage **2**, as is evident when the two are overlaid (Figure S57). The calculated structure suggests that the carbonyl oxygen significantly impacts the size and polarity of the cage windows and cavity (Figures 2 and S57).

**Figure 3:**
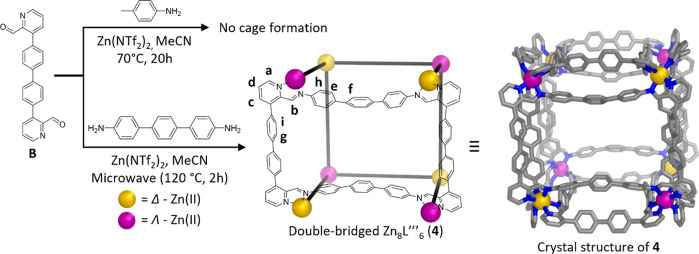
Self-assembly of double-bridged zinc(II) cages based on subcomponent **B**. (Top) No cage formation was observed upon reaction of the subcomponents **B** and *p*-toluidine with Zn(NTf_2_)_2_ under similar conditions. (Bottom) The self-assembly of subcomponents **B** and 4,4″-diamino-*p*-terphenyl with Zn(NTf_2_)_2_, forming *T*_h_-symmetric double-bridged Zn_8_L‴_6_ cage **4**, illustrated as a schematic model and view of the crystal structure of the (tetrakis(4-fluorophenyl)borate) salt. Proton assignments are derived from NMR spectra (Figures S58–S62 in the Supporting Information). Hydrogen atoms and counteranions are omitted for clarity.

To confirm the transferability of the double-bridging strategy to other cages, we prepared extended subcomponent **B** (Figures 3, S6–S10, pages 6–9 in the SI). A larger single-bridged pseudo-cube similar to **1** was not obtained when **B** and *p*-toluidine were mixed with Zn(NTf_2_)_2_ under the same conditions used for the formation of **1**. However, the reaction of subcomponent **B** (1.5 equiv), 4,4″-diamino-*p*-terphenyl (1.5 equiv), and zinc(II) bis(trifluoromethylsulfonyl)imide (Zn(NTf_2_)_2_, 1.1 equiv) in acetonitrile in a microwave reactor resulted in the formation of the extended double-bridged [Zn_8_L‴_6_](NTf_2_)_16_ pseudo-cube **4** (Figure 3), as confirmed by HR-ESI-MS (Figures S67–S70), single-crystal X-ray diffraction, and NMR, as detailed below.

Single crystals of **4** were obtained by vapor diffusion of benzene into an acetonitrile solution of **4** containing excess sodium (tetrakis(4-fluorophenyl)borate). The X-ray structure of **4** confirmed the formation of a *T*_h_ symmetric cage (see Figures 3 and S73, with details given on SI pages 43–47).

Solution NMR characterization (Figures S58–S62) gave results consistent with the X-ray structure. We infer the mutual proximity of the aromatic hydrogens of the double-bridged edges to result in restricted rotational freedom in solution, which led to a broadening of the proton signals in the ^1^H NMR spectrum. This proximity was confirmed by NOESY measurements (Figure S63). At elevated temperatures, increased rotational freedom resulted in a sharpening of the phenylene proton signals (Figure S64). DOSY NMR measurements confirmed the presence of a single species with a solvodynamic radius of 22.9 Å (Figure S65). As with the DOSY measurements of **1**, **2**, and **3**, the experimentally determined solvodynamic radius exceeded the radius of 17.6 Å determined from the crystal structure of **4** (Figure S73). Here too we attribute this observation to an extended solvent/anion shell, due to metal charge exposure to the external environment. As with **2** and **3**, only one Tf_2_N^–^ signal was observed in the ^19^F NMR spectrum of **4**, consistent with a poorly enclosed cage cavity (Figure S66).

### Stability and Robustness Investigations

**Figure 4:**
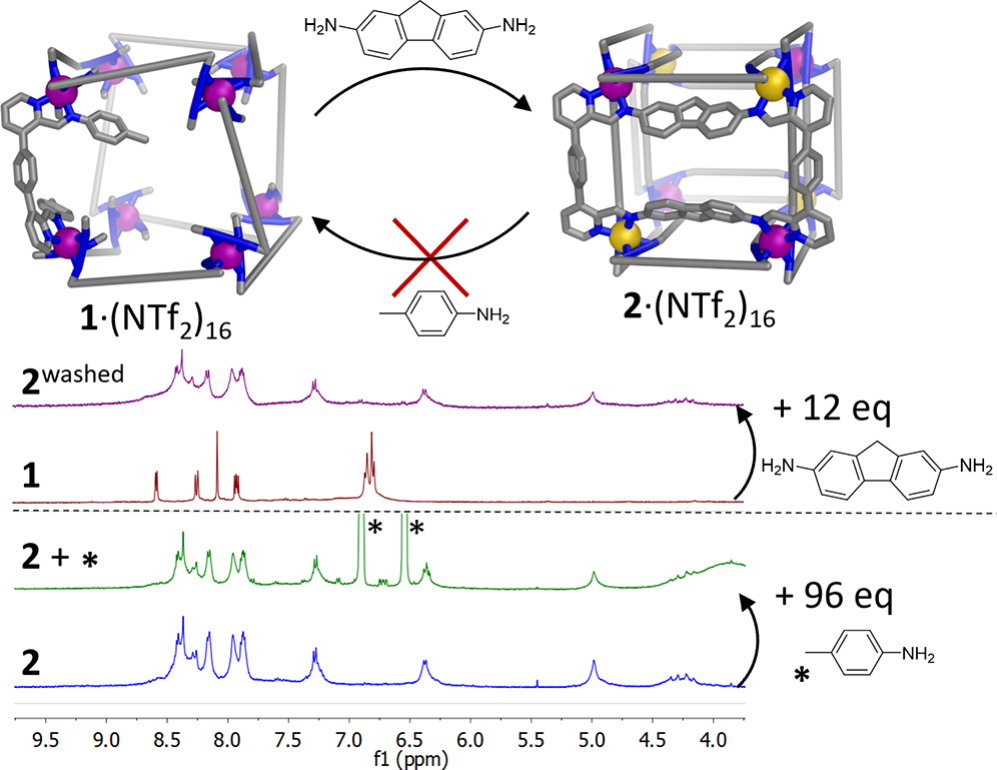
Comparison of the stabilities of cages **1** and **2**. **1** converted into **2** upon addition of 2,7-diaminofluorene (12 equiv), whereas conversion of **2** to **1** was not observed even upon addition of 200 equiv *p*-toluidine. After conversion from **1** to **2**, the sample was washed with Et_2_O to remove free *p*-toluidine and minor impurities. The spectrum before washing is given in Figure S74 in the Supporting Information.

Double-bridging leads to an increase in ligand topicity. This increase should stabilize a coordination cage due to a higher density of linkages holding the structure together, requiring more bonds to be broken in order to disrupt the structure.^58^ In contrast to previous cases,^43^ the novel vertex geometry generated by dialdehydes **A** and **B** facilitates vertex bridging without disrupting the polyhedral framework of the metal–organic cage. To gauge the effects of double-bridging on cage stability, the conversion from **1** to **2** was investigated experimentally and computationally. Upon addition of 12 equiv 2,7-diaminofluorene to cage **1** and heating at 70 °C over 20 h, complete conversion from **1** to **2** was observed (Figures 4 and S74). In contrast, the conversion of **2** to **1** was not observed even upon addition of 200 equiv of *p*-toluidine and prolonged heating (Figures 4 and S75). These observations indicate that cage **2** is thermodynamically favored over cage **1**. As the conversion from **1** to **2** results in a change in electronic structure, this process could be followed by UV–vis spectroscopy (Figures S76, S77). While increasing the temperature increased both reaction rates and conversion, substitution did not follow straightforward first or second order rate laws (Figure S77, right). Furthermore, addition of a smaller amount of 2,7-diaminofluorene resulted in a mixture of cage **1** and **2**, and the observation of undefined intermediates (Figure S78).

To investigate the thermodynamic driving force for the conversion of **1** to **2** in detail and gain a deeper understanding of the effect of double-bridging on the stability of the cages, semiempirical quantum mechanical calculations were performed (structures based on GFN2-xTB calculations are given in Figures S79, S80, with details given on SI pages 51–53). The optimized structures were very similar to the crystal structures of **1** and **2**. Calculations at both the GFN2-xTB and DFT level indicated that **1** is enthalpically favored over **2**. Nevertheless, the entropic contribution outweighs the enthalpic term, leading to a driving force for the conversion from **1** to **2** of Δ*G* = −30.39 kcal mol^–1^ (Δ*H* = 101.40 kcal mol^–1^), *T*Δ*S* = 131.70 kcal mol^–1^ (r2SCAN-3c//GFN2-xTB).

Cage **2** is also more rigid than **1**, as confirmed by molecular dynamics (MD) simulations (details on SI pages 53–55) using a classical force-field, where cage **1** was found to collapse. The cage framework was maintained only when intramolecular noncovalent interactions were better described using the GFN2-xTB approach. In contrast, **2** remained rigid in all the simulations. Molecular flexibility is qualitatively correlated with atomic movement over a given time interval, as indicated by larger integrated atomic displacement, a high average Cartesian coordinate root-mean-square deviation, or low fluctuation in total energy during MD simulation. For all the metrics considered, **2** consistently exhibited greater rigidity than **1** (Table S4, Figure S82).

We next sought to investigate whether the stability increase through double-bridging also translates to greater robustness against chemical stimuli. Cages **1** and **2** were exposed to various such stimuli chosen for their relevance to real-world applications. Upon addition of 12 equiv of aniline, decomposition of cage **1** was observed, while cage **2** remained intact even following the addition of 100 equiv of aniline (Figures S83, S84). The effect of salt addition was then investigated through addition of TBA^+^TfO^–^. While cage **1** decomposed upon addition of 20 equiv of this salt, cage **2** remained intact in the presence of 30 equiv of TBA^+^TfO^–^ (Figures S85, S86). Cage resistance to transmetalation was investigated upon addition of Fe(NTf_2_)_2_, as the iron(II) congeners of **1** and **2** were not obtained by direct self-assembly. Addition of 8 equiv Fe(NTf_2_)_2_ to a solution of cage **1** resulted in an immediate color change to produce a purple solution, consistent with the formation of low-spin iron(II) pyridyl-imine complexes.^59^ Decomposition of **1** was further confirmed by ^1^H NMR (Figure S87). In contrast, following the addition of 24 equiv of Fe(NTf_2_)_2_ to a solution of **2**, no color change was observed, and the ^1^H NMR spectrum confirmed that cage **2** remained intact (Figure S88). Cage tolerance toward added ligands was investigated by the addition of neat deuterated DMSO, which can form [Zn(DMSO)_6_]^2+^ with zinc(II).^60^ As in other cases,^61,62^ the low stability of **1** resulted in full decomposition at 7 vol % DMSO, while **2** stayed intact even in 33 vol % DMSO (Figures S89, S90). Furthermore, cage **2** exhibited a higher thermal stability in the solid state compared to cage **1** (Figures S91, S92). We conclude that the greater stability of **2** compared to **1** is based on increased topicity, and thus preorganization, of the tetratopic ligands of **2**.

### Host–Guest Chemistry

The host–guest chemistry of all cages was investigated by the addition of 10 equiv of an anionic guest—BF_4_^−^, PF_6_^−^, TfO^−^, BPh_4_^F−^ (tetrakis(4-fluorophenyl)borate), and BPh_4_^CF3−^ (tetrakis[3,5-bis(trifluoromethyl)phenyl]-borate)—to a solution of the host. Even though cage **1** exhibited decomposition upon addition of larger amounts of salts, addition of 10 equiv of one of these anions did not result in decomposition (Figure S93). While the addition of the largest anionic guest BPh_4_^CF3–^ did not result in any shifts of the signals, the addition of BPh_4_^F–^ resulted in small shifts of all proton signals. In contrast, the addition of the smaller anionic guests BF_4_^–^, PF_6_^–^, or TfO^–^ only affected the signals of protons e and f, hinting at localization of interactions of the small anions at the cage faces (Figure S94). Similar interactions were also observed for NTf_2_^–^ (Figure S24).

Cage **2** bound all of the tested anionic guests except for BPh_4_^CF3–^ (Figure S95). The addition of 10 equiv of one of the small anions BF_4_^–^, PF_6_^–^, or TfO^–^ resulted in shifts of the ^1^H NMR signals of protons c and j, which point toward the cage cavity. We thus conclude that these anions with van der Waals volumes between 53.2 to 84.3 Å^3^ bind within the 353.9 Å^3^ cavity of **2** (Figure S96).^63^ In contrast, the addition of BPh_4_^F–^, with a van der Waals volume of 358.2 Å^3^, results in shifts of protons g and h, consistent with exterior binding of the anion at the cage windows. The presence of two binding sites that are selective for specific guests permits the simultaneous binding of BF_4_^–^ and BPh_4_^F–^ in the interior and exterior binding sites, respectively. This behavior is reflected in shifts of both the c/j and g/h protons upon simultaneous addition of both guests. Detailed investigation of the host–guest binding by NMR titration experiments, including determination of the binding constants, proved challenging due to the limited solubility of cage **2** following addition of BF_4_^–^, PF_6_^–^, or TfO^–^, which resulted in precipitation of the cage upon anion exchange. Furthermore, the lack of a suitable chromophore within **2** prevented the determination of host–guest isotherms and the associated binding constants by UV–vis titration measurements.

Host–guest experiments with congener **3** indicated that only the addition of BPh_4_^F–^ resulted in NMR shifts of protons *g* and *h*, indicating exterior binding on the cage window, as with cage **2** (Figure S97). The addition of BF_4_^–^, PF_6_^–^, or TfO^–^ did not lead to significant NMR shifts, indicating a lack of interaction. This result indicates that the incorporation of a carbonyl oxygen prevents encapsulation of small anionic guests, despite the similarity of the framework of **3** to that of **2** (Figure S98). Cage **4** exhibited weak interactions between BPh_4_^F–^ and the edges of the pseudo-cube, as evidenced by small NMR shifts of protons e and f (Figure S99). We attribute the lack of guest binding to the porous architecture of cage **4** (Figure S100).

## Conclusion

Our approach—the double-bridging strategy—facilitates the formation of highly robust self-assembled zinc(II) metal–organic cages. This strategy is based on a novel dialdehyde subcomponent that produces a vertex motif in which substituents point toward the cage faces, enabling covalent bridging to increase ligand topicity. WAlthough the architecture is not affected by double-bridging, the cage chirality is transformed from point group *O* to achiral *T*_h_. Our strategy permits the introduction of functional groups in proximity to the cage cavity, influencing host–guest binding. The topicity increase through double-bridging enhances the cage stability sufficiently to enable the preparation of a large double-bridged cage that could not be obtained as its single-bridged congener. Double-bridged metal–organic cages did not decompose upon treatment with chemical stimuli that destroyed their single-bridged congeners, withstanding transmetalation, the addition of competing ligands and anilines. This thermodynamic and kinetic stability is crucial for the application of metal–organic cages in real-world scenarios, for instance in the purification of industrially relevant molecules out of complex reaction mixtures.^2,64^ The application of the double-bridging strategy in other metal–organic cage systems thus appears very promising, potentially creating new use cases for these systems by increasing their robustness, while simultaneously offering a new route to endofunctionalisation.

## Supporting Information Available

(1) General Information (2) Synthesis and Characterization of Subcomponents (3) Subcomponent Self-Assembly (4) Single-crystal X-ray Diffraction (5) Conversion from **1** to **2** (6) Robustness Investigations of **1** and **2** (7) Host–Guest Experiments (PDF)

## Abbreviations

NMR: nuclear magnetic resonance
NOESY: nuclear overhauser effect spectroscopy
DOSY: diffusion ordered spectroscopy
HOESY: heteronuclear overhauser enhancement spectroscopy
HR-ESI-MS: high resolution electrospray ionization mass spectrometry
DFT: density functional theory
MD: molecular dynamics
H: enthalpy
T: temperature
S: entropy
equiv: equivalents
DMSO: dimethyl sulfoxide
TBA^+^: tetrabutylammonium
Tf_2_N^–^: (trifluoromethylsulfonyl)imide
OTf^–^: trifluoromethanesulfonate
BPh_4_^F–^: (tetrakis(4-fluorophenyl)borate)
BPh_4_^CF3–^: (tetrakis[3,5-bis(trifluoromethyl)phenyl]-borate)
