# Supplementary material for: Double-Bridging Increases the Stability of Zinc(II) Metal–Organic Cages
Source: J Am Chem Soc. 2024 Nov 4;146(45):30958–65. doi: 10.1021/jacs.4c09742 (PMC11565643; doi:10.1021/jacs.4c09742)
Supplement: Supplementary file 1 — ja4c09742_si_001.pdf [file ja4c09742_si_001.pdf]

Supporting Information

Double-Bridging Increases the Stability of Zinc(II) Metal-Organic Cages

Hannah Kurz, C. P. Paula Teeuwen, Tanya K. Ronson, Jack B. Hoffman, Philipp Pracht, David J. Wales, and Jonathan R. Nitschke*

Experimental Section

1. General Information 1
2. Synthesis and Characterisation of Subcomponents 2
Subcomponent **A** 2
2,7-Diamino-9-fluorenone 4
4,4'-Biphenyldiboronic acid pinacol ester 6
Subcomponent **B** 7
3. Subcomponent Self-Assembly 9
1·$(NTf_2)_{16}$ 9
2·$(NTf_2)_{16}$ 18
3·$(NTf_2)_{16}$ 28
4·$(NTf_2)_{16}$ 36
4. Single-crystal X-ray Diffraction 43
Specific refinement details for **1**·$(SbF_6)_{16}$ [+ solvent] 44
Specific refinement details for **2**·$(PF_6)_{16}$ [+ solvent] 45
Specific refinement details for **4**·$(BPh_4^F)_{16}$ [+ solvent] 46
5. Conversion from **1** to **2** 47
NMR and UV-vis studies 47
Computational Studies 50
6. Robustness Investigations of **1** and **2** 55
7. Host-Guest Experiments 61
8. Literature 65

1. General Information

Unless otherwise stated, all starting materials, reagents, and solvents were purchased from commercial suppliers and used without further purification. Air or water sensitive reactions were carried out under N_2 atmosphere. Self-assembly reactions were either performed in CD_3CN that was stored over molsieve or freshly distilled MeCN. For the self-assembly of **2**, **3** and **4**, a microwave reactor (Discover SP, CEM) was used. Centrifugation was performed at 8800 rpm with a 5804/5804R benchtop centrifuge. After the reaction of all cages, the acetonitrile reaction mixtures were filtered

through a glass fibre plug. After concentrating the solvent, the cages were precipitated by addition of Et_2O, centrifuged and washed two times with Et_2O.

NMR spectra were recorded using the following instruments: Bruker 400 MHz Avance III HD Smart Probe (routine ^{1}H NMR, ^{19}F NMR, ^{1}H DOSY, ^{1}H-^{19}F HOESY), Bruker 500 MHz Avance III HD Smart Probe (VT-NMR), Bruker 500 MHz Avance III DCH Cryoprobe (full characterisation: ^{1}H, ^{13}C, COSY, HSQC, HMBC, NOESY, ROESY). Chemical shifts (δ) are given in parts per million (ppm) from low to high field and referenced using the residual solvent signal (^{1}H, ^{13}C). ^{19}F NMR spectra were referenced to the standard hexafluorobenzene located in a small capillary (-164.9 ppm, CD_3CN). Coupling constants (J) are given in Hertz (Hz). The multiplicity is reported using the following abbreviations: singlet (s), doublet (d), broad (br).

High-resolution electrospray ionization mass spectrometry (HR-ESI-MS) were recorded either on a Waters Synapt G2-Si spectrometer infused from a Harvard syringe pump or on a Waters Xevo G2-S bench top QTOF.

UV-Vis measurements were performed on a Cary 5000 UV-Vis-NIR spectrophotometer from Agilent Technologies. The temperature was adjusted by a dual cell peltier accessory by Cary. A 1 mm path-length cuvette was used. Melting points were determined with an IA9100 Electrothermal instrument.

2. Synthesis and Characterisation of Subcomponents

Subcomponent **A**

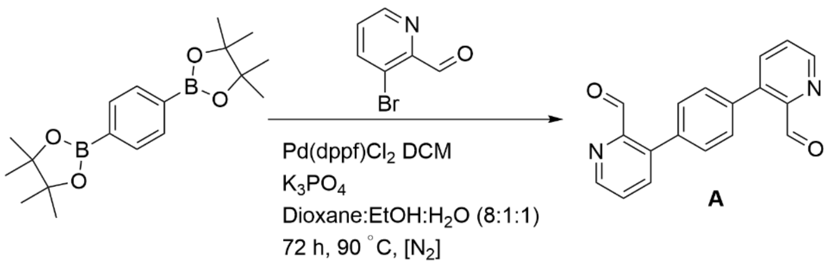

1,4-Benzenediboronic acid bis(pinacol) ester (0.25 g, 0.76 mmol, 1 eq), 3-bromo-2-formylpyridine (0.38 g, 2.05 mmol, 2.7 eq), $Pd(dppf)Cl_2{\cdot}DCM$ (62 mg, 0.076 mmol, 0.1 eq), and K_3PO_4 (0.56 g, 2.65 mmol, 3.5 eq) were heated in 25 mL of a degassed solvent mixture (dioxane:EtOH:H_2O (8:1:1)) for 48 h at 90°C under nitrogen. The black suspension was filtered hot over celite and the solvent was removed in vacuo. The crude solid was extracted with DCM and washed 3 times with 10 mL water and one time with 10 mL brine. After drying the organic phase over $MgSO_4$, the solvent was removed in vacuo and the brown precipitate was washed several times with ether. The residue was purified by column chromatography (DCM:MeOH 95:5) yielding a light brown solid (0.14 g, 4.86 mmol, 64%).

^{1}H-NMR (500 MHz, $CDCl_3$, 298 K): δ = 10.17 (s, 1H, a), 8.88 (dd, ^3J = 4.6 Hz, ^4J = 1.55 Hz, 1H, b); 7.88 (dd, ^3J = 7.8 Hz, ^4J = 1.55 Hz, 1H, c); 7.60 (dd, ^3J = 7.8 Hz, ^4J = 4.6 Hz, 1H, d); 7.49 (s, 2H, e). **^{13}C-NMR** (126 MHz, $CDCl_3$, 298 K): δ = 192.0, 149.7, 149.3, 139.7, 139.6, 136.9, 129.7, 126.9. **ESI-HRMS**: Found m/z = 289.0988 $[M+H]^+$, C18H12N2O2+H calculated m/z = 289.0977. **Melting point**: 207°C.

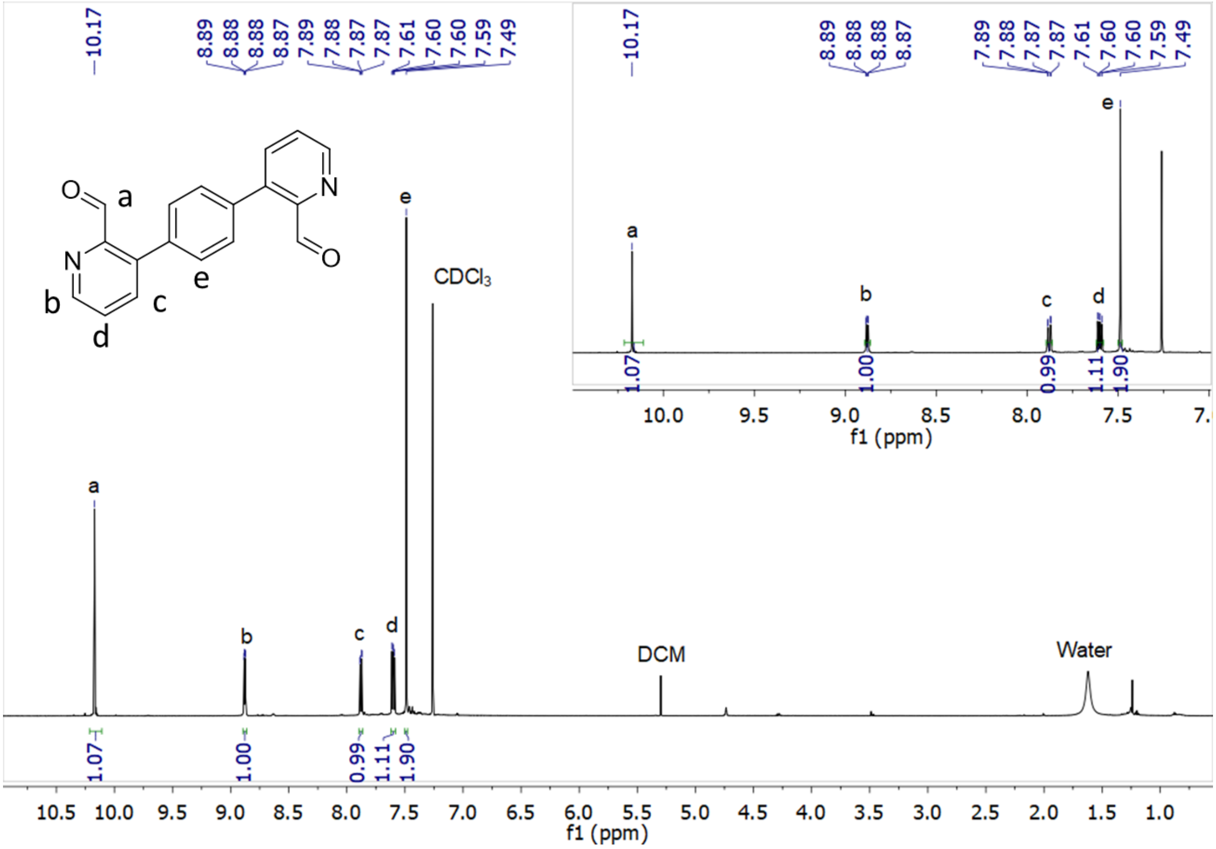

Figure S1: ^{1}H NMR spectrum of subcomponent A (500 MHz, $CDCl_3$, 298 K).

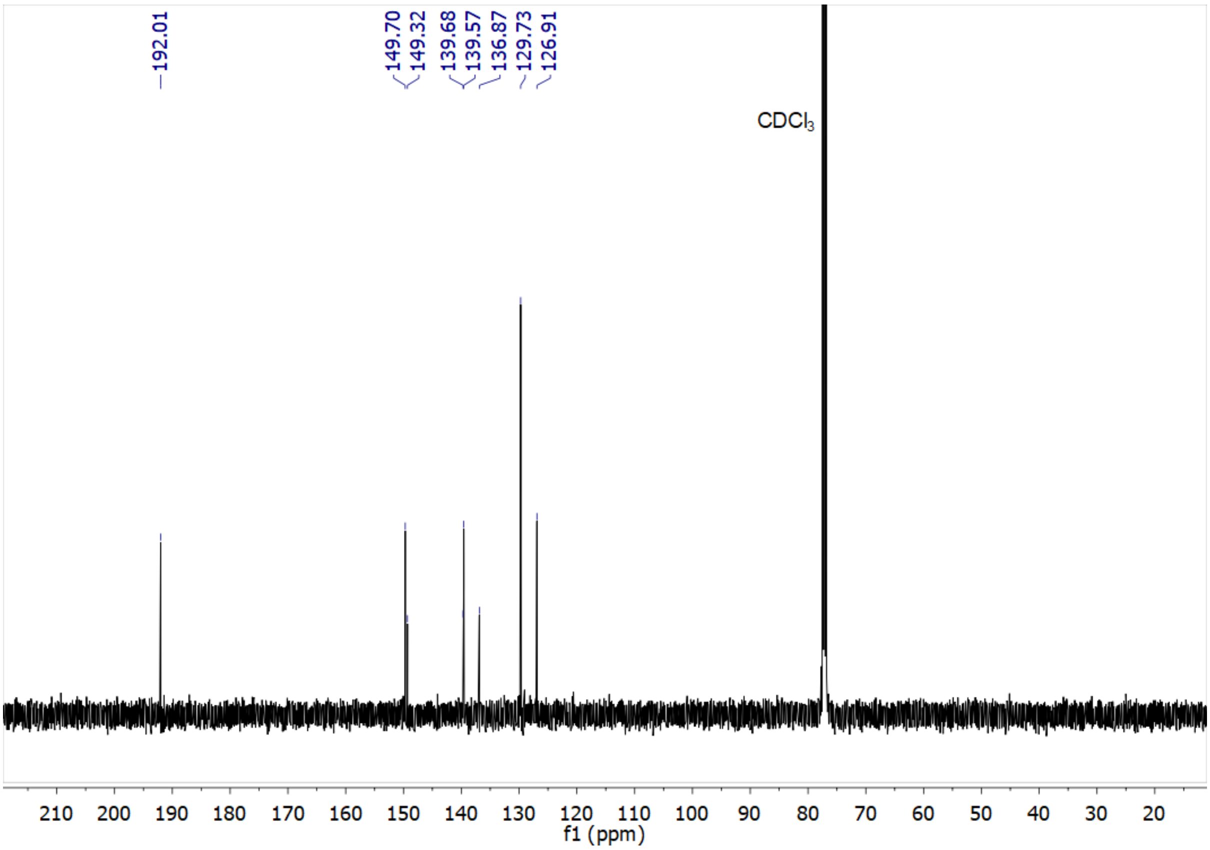

Figure S2: ^{13}C NMR spectrum of subcomponent A (126 MHz, $CDCl_3$, 298 K).

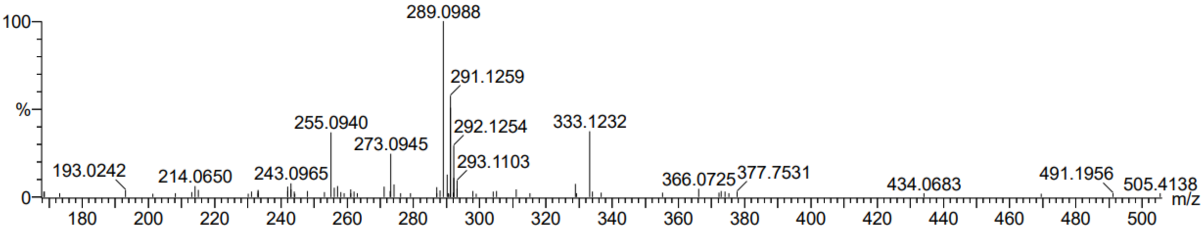

*Figure S3: High resolution ESI-MS spectrum of subcomponent **A**.*

<u>2,7-Diamino-9-fluorenone</u>

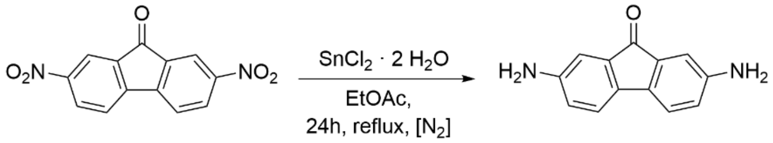

2,7-Dinitro-9-fluorenone was synthesized *via* a modified literature procedure.[1] 2,7-Dinitro-9-fluorenone (0.20 g, 0.74 mmol, 1 eq) and tin(II) chloride dihydrate (1.17 g, 5.18 mmol, 7 eq) were heated in 10 mL degassed dry ethyl acetate for 24 h under reflux and nitrogen. After cooling, the reaction mixture was poured over 10 g of ice and neutralised with a saturated NaHCO3 aqueous solution to pH 8. Extract six times with 10 mL EtoAc and dry combined organic phase over MgSO4. The product was obtained as a dark purple solid (0.11 g, 0.54 mmol, 73%) after removal of the solvent in vacuo.

^{1}H-NMR (500 MHz, CD_3CN, 298 K): δ = 7.14 (d, ^{3}J = 7.9 Hz, 2H, a); 6.78 (d, ^{3}J = 2.3 Hz, 2H, b); 6.67 (dd, ^{3}J = 2.3 Hz, ^{3}J = 7.9 Hz, 2H, c); 4.29 (s, 4H, d). **^{13}C-NMR** (126 MHz, CD_3CN, 298 K): δ = 149.1, 136.7, 136.1, 121.4, 120.6, 119.0, 111.3. **ESI-HRMS**: Found m/z = 211.0857 $[M+H]^+$, C13H10N2O+H calculated m/z = 211.0866.

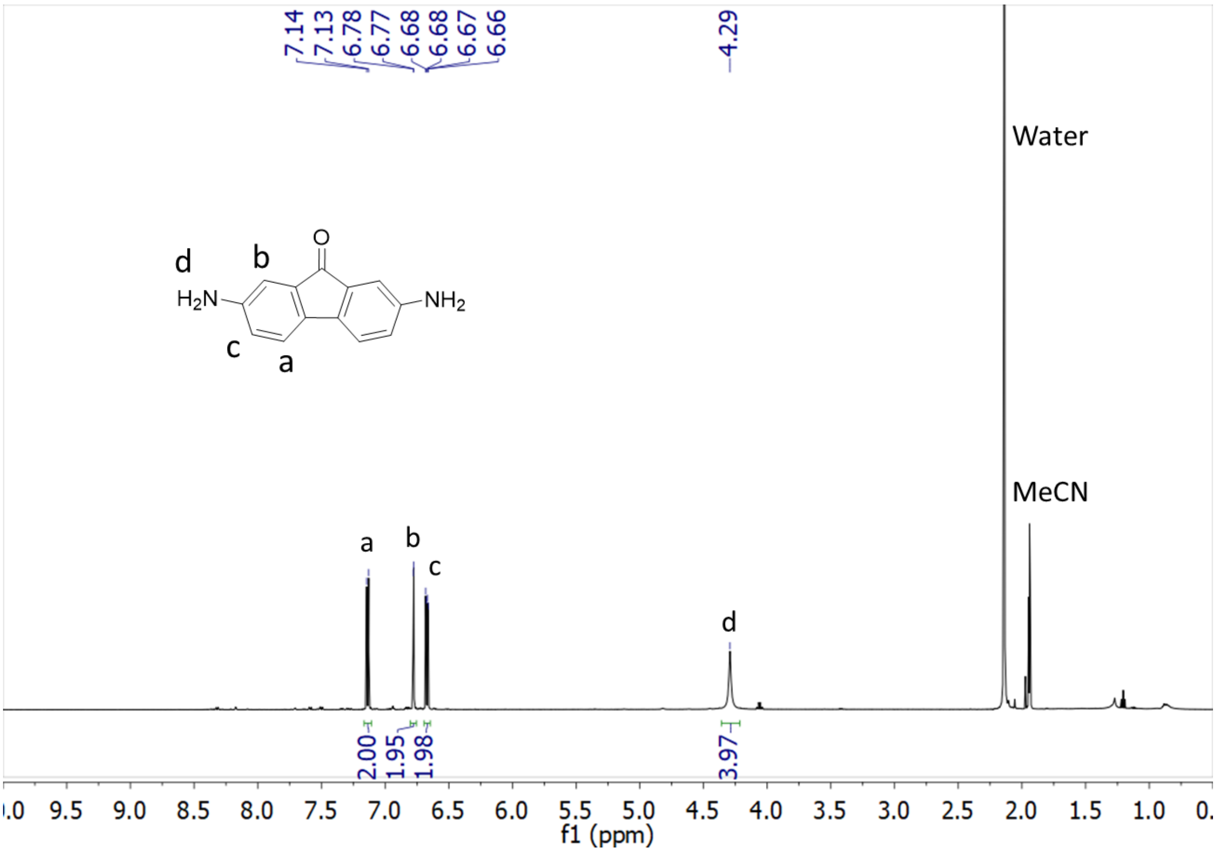

Figure S4: ^{1}H NMR spectrum of 2,7-diamino-9-fluorenone (500 MHz, CD_3CN, 298 K).

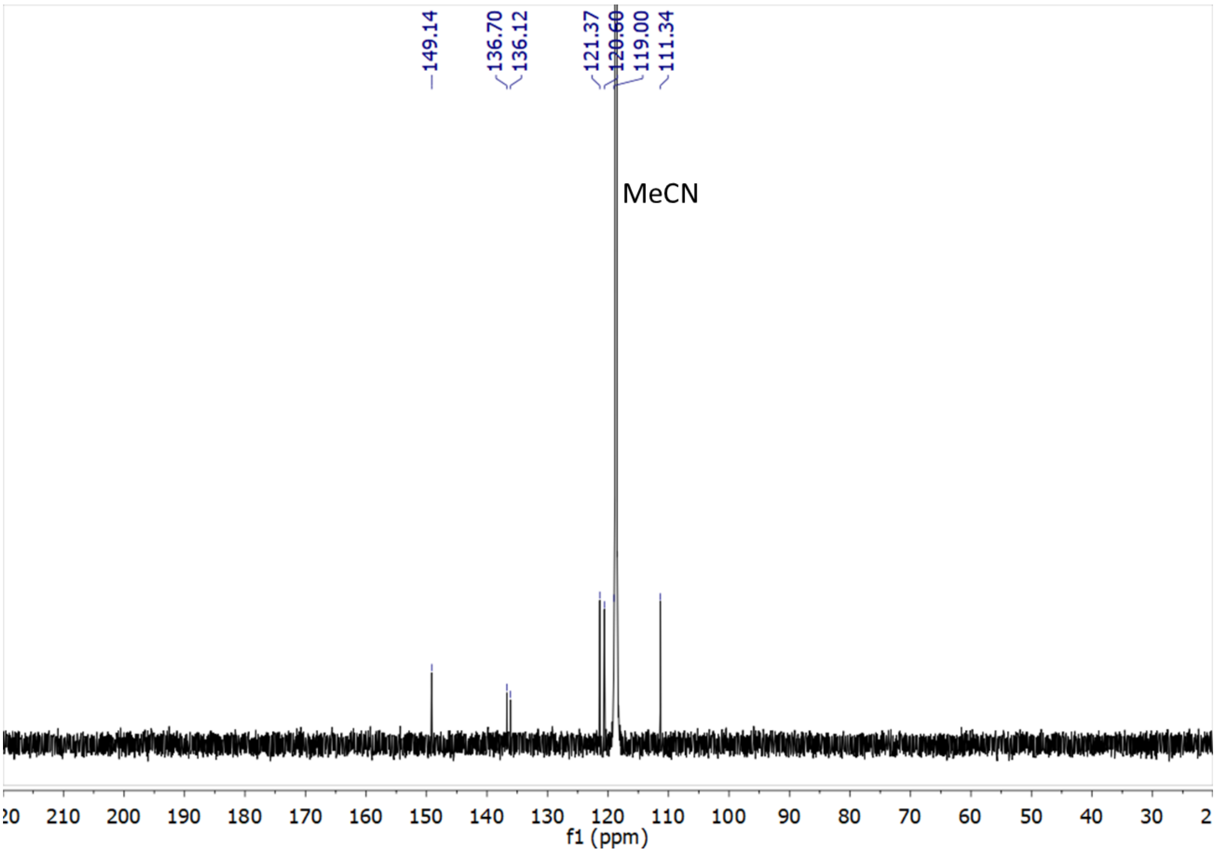

Figure S5: ^{13}C NMR spectrum of 2,7-diamino-9-fluorenone (126 MHz, CD_3CN, 298 K).

4,4'-Biphenyldiboronic acid pinacol ester

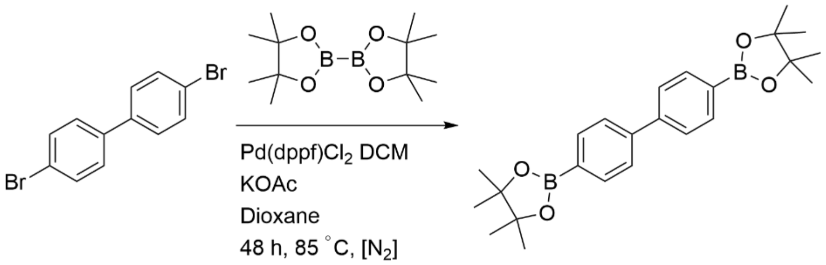

4,4'-Biphenyldiboronic acid pinacol ester was synthesized *via* a modified literature procedure.[2] 4,4'-Dibromobiphenyl (0.50 g, 1.60 mmol, 1 eq), bis(pinacolato)diboron (0.90 g, 3.53 mmol, 2.2 eq), Pd(dppf)Cl_2·DCM (65 mg, 0.080 mmol, 0.05 eq), and potassium acetate (0.47 g, 4.81 mmol, 3 eq) were heated in 10 mL degassed dioxane for 48 h at 85°C under nitrogen. The black suspension was filtered hot over celite and the solvent was removed in vacuo. The crude solid was extracted with DCM and washed 3 times with 10 mL water and one time with 10 mL brine. After drying the organic phase over $MgSO_4$, the solvent was removed in vacuo and the light brown precipitate was washed several times with ether. The residue was purified by column chromatography (DCM) yielding a white solid (0.55 g, 1.35 mmol, 85%).

^{1}H-NMR (500 MHz, $CDCl_3$, 298 K): δ = 7.88 (d, ^3J = 8.3 Hz, 1H, a); 7.63 (d, ^3J = 8.2 Hz, 1H, b); 1.36 (s, 6H, c). **^{13}C-NMR** (126 MHz, $CDCl_3$, 298 K): δ = 143.8, 135.4, 126.7, 84.0, 25.0. **ESI-HRMS**: Found m/z = 407.2580 $[M+H]^+$, C24H32B2O4+H calculated m/z = 407.2565.

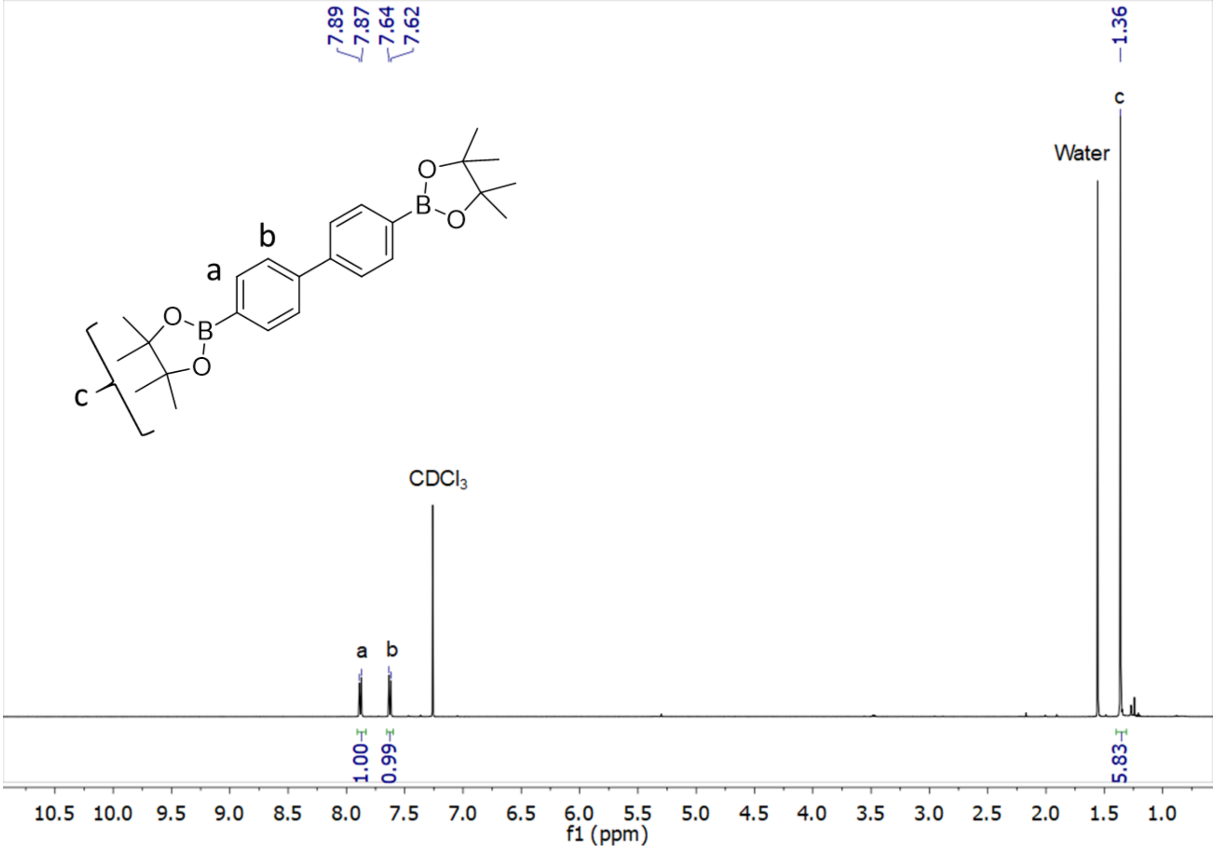

Figure S6: ^{1}H NMR spectrum of 4,4'-biphenyldiboronic acid pinacol ester (500 MHz, $CDCl_3$, 298 K).

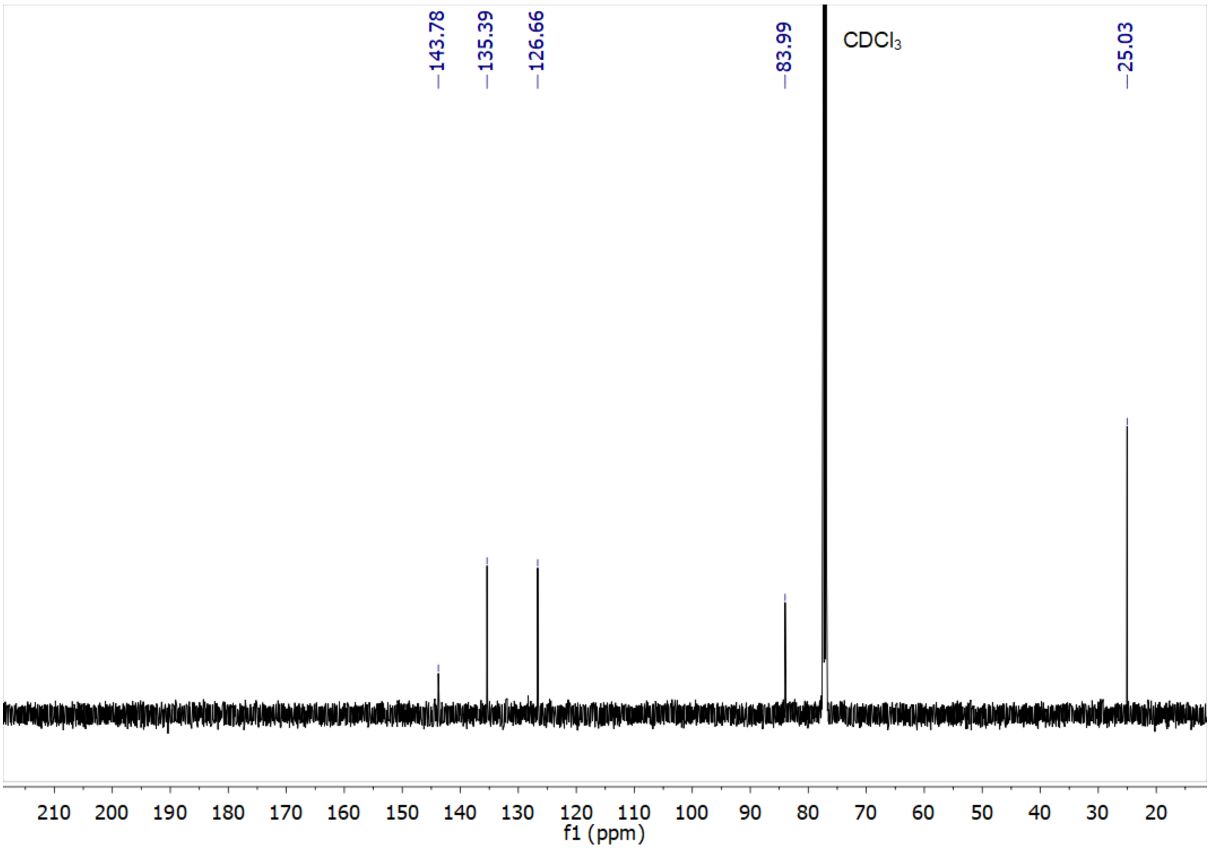

Figure S7: ^{13}C NMR spectrum of 4,4'-biphenyldiboronic acid pinacol ester (126 MHz, $CDCl_3$, 298 K).

Subcomponent **B**

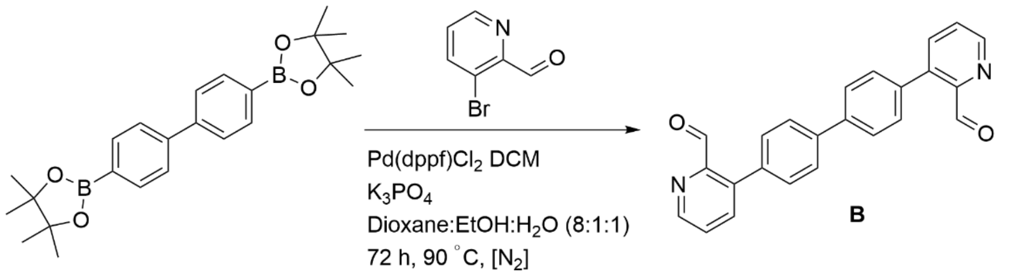

4,4'-Biphenyldiboronic acid pinacol ester (0.20 g, 0.49 mmol, 1 eq), 3-bromo-2-formylpyridine (0.25 g, 1.33 mmol, 2.7 eq), Pd(dppf)Cl_2·DCM (40 mg, 0.049 mmol, 0.1 eq), and K_3PO_4 (0.37 g, 1.72 mmol, 3.5 eq) were heated in 20 mL of a degassed solvent mixture (dioxane:EtOH:H_2O (8:1:1)) for 48 h at 90°C under nitrogen. The black suspension was filtered hot over celite and the solvent was removed in vacuo. The crude solid was extracted with DCM and washed 3 times with 10 mL water and one time with 10 mL brine. After drying the organic phase over $MgSO_4$, the solvent was removed in vacuo and the brown precipitate was washed several times with ether yielding a light brown solid (0.14 g, 3.84 mmol, 78%).

^{1}H-NMR (500 MHz, $CDCl_3$, 298 K): δ = 10.17 (s, 1H, a); 8.87 (dd, ^3J = 4.6 Hz, ^4J = 1.6 Hz, 1H, b); 7.87 (dd, ^3J = 7.8, ^4J = 1.5 Hz, 1H, c); 7.78 (d, ^3J = 8.3 Hz, 2H, d); 7.59 (dd, ^3J = 4.6 Hz, ^3J = 7.8 Hz, 1H, e); 7.50 (d, ^3J = 8.3 Hz, 2H, f). **^{13}C-NMR** (126 MHz, $CDCl_3$, 298 K): δ = 191.9, 149.6, 149.3, 140.6, 140.3, 139.5, 135.8, 130.2, 127.5, 126.9. **ESI-HRMS**: Found m/z = 365.1287 $[M+H]^+$, C24H16N2O2+H calculated m/z = 365.1290. **Melting point**: 190°C.

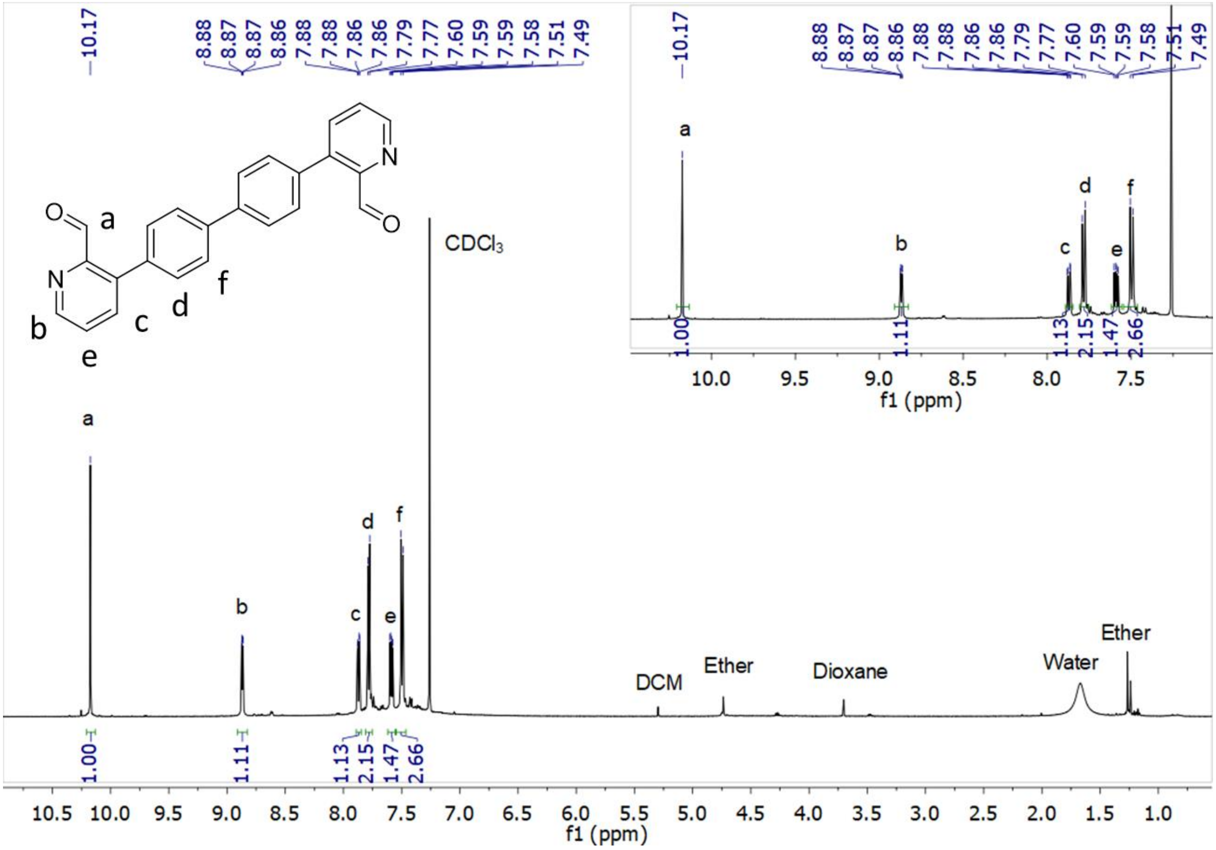

Figure S8: ^{1}H NMR spectrum of subcomponent B (500 MHz, $CDCl_3$, 298 K).

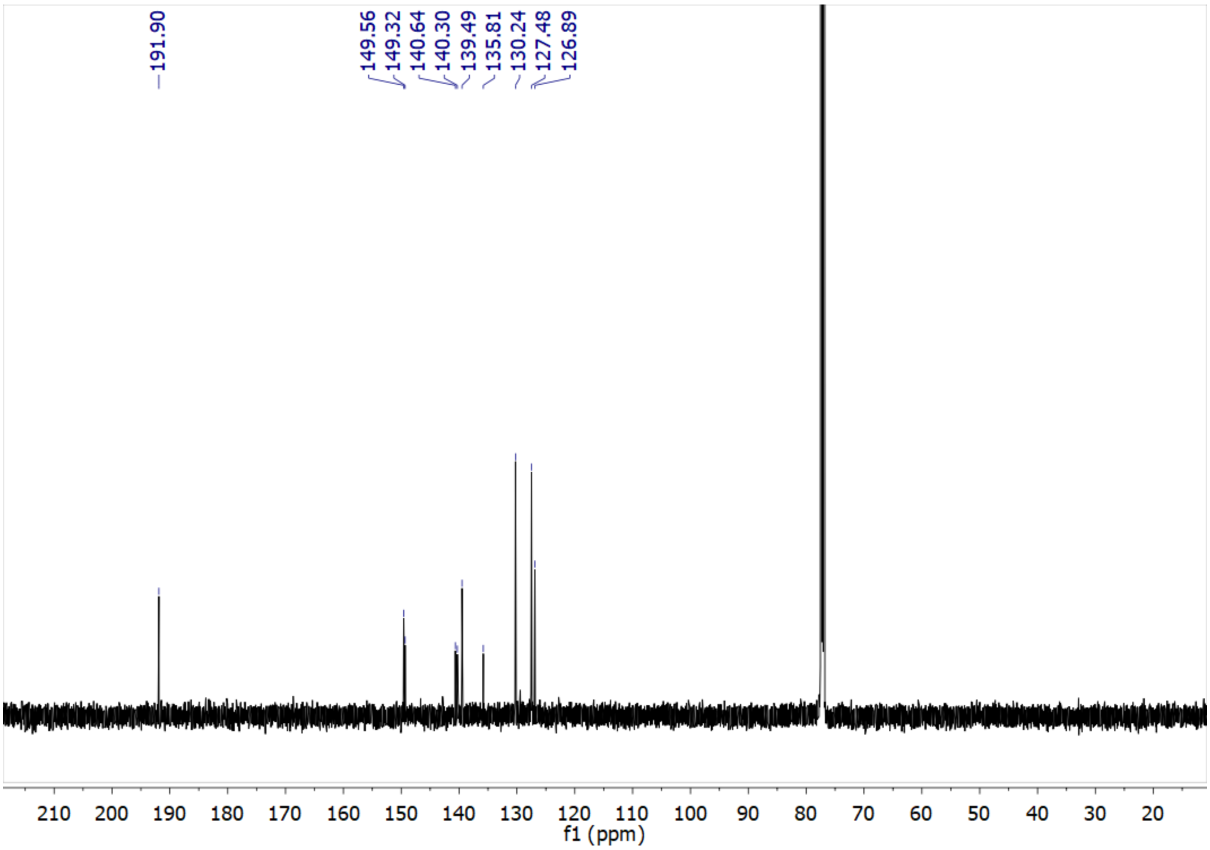

Figure S9: ^{13}C NMR spectrum of subcomponent B (126 MHz, $CDCl_3$, 298 K).

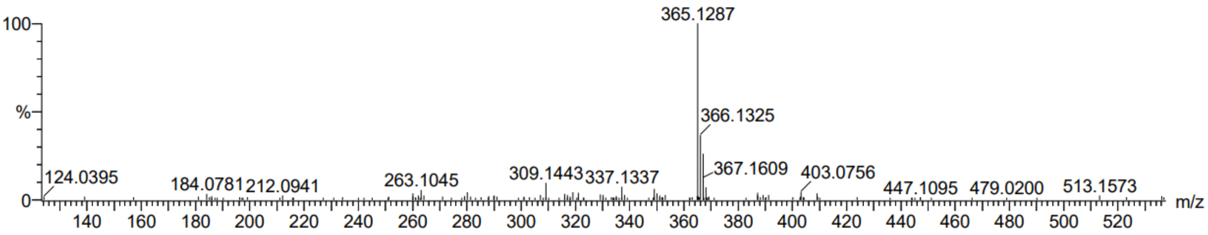

*Figure S10: High resolution ESI-MS spectrum of subcomponent **B**.*

3. Subcomponent Self-Assembly

1·$(NTf_2)_{16}$

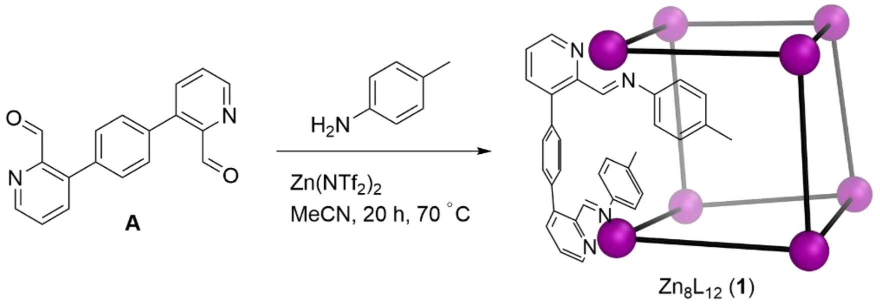

Subcomponent **A** (10.0 mg, 34.7 µmol, 1.5 eq), *p*-toluidine (7.4 mg, 69.4 µmol, 3.0 eq), and $Zn(NTf_2)_2$ (14.5 mg, 23.1 µmol, 1.0 eq) were heated in 2 mL freshly distilled MeCN for 20 h at 70°C. After cooling, the reaction mixture was filtered over a glass fibre plug, concentrated to 0.5 mL and precipitated with 15 mL Et_2O. The grey precipitate was collected via centrifugation, washed two times with 15 mL Et_2O, and dried in vacuo. **1**·$(NTf_2)_{16}$ was obtained as a grey solid (m = 25.8 mg, 84%).

^{1}H-NMR (700 MHz, CD_3CN, 298 K): δ = 8.58 (d, ^3J = 2.8 Hz, 1H, a); 8.24 (d, ^3J = 4.6 Hz, 1H, b); 8.07 (s, 1H, c); 7.92 (dd, ^3J = 4.6 Hz, ^3J = 2.9 Hz, 1H, d); 6.85 (d, ^3J = 3.6 Hz, 2H, e); 6.79 (d, ^3J = 4.6 Hz, 2H, f); 6.96-6.72 (broad d, 2H, g); 0.93 (s, 3H, h). **^{13}C-NMR** (176 MHz, CD_3CN, 298 K): δ = 162.6, 149.8, 145.0, 144.9, 143.7, 141.2, 140.4, 135.7, 131.2, 130.9, 130.8, 122.5, 120.9 (q, *J* = 322.1 Hz, NTf_2), 20.26. **^{19}F-NMR** (376 MHz, CD_3CN, 298 K): δ = -78.6, -80.4. **ESI-HRMS**: ([**1**·$(NTf_2)_{16}$] = (C32H26N4)12Zn8(C2F6NO4S2)16 m/z = 1487.2 [**1**·$(NTf_2)_{10}$]$^{6+}$ (calc. 1487.3), 1840.6 [**1**·$(NTf_2)_{11}$]$^{5+}$ (calc. 1840.7), 2370.7 [**1**·$(NTf_2)_{12}$]$^{4+}$ (calc. 2371.0).

Reaction of subcomponent **A**, *p*-toluidine, and $Zn(NTf_2)_2$ via microwave heating at 120°C for 2h also resulted in the formation of cage **1**. Reaction of subcomponent **A**, *p*-toluidine, and other metal salts ($Zn(BF_4)_2$, $Fe(NTf_2)_2$) under similar conditions did not result in the formation of the respective cages. Reaction of subcomponent **A**, *p*-toluidine, and $Zn(OTf)_2$ under similar conditions results in the formation of a small amount of the respective cage along with impurities. Reaction of subcomponent **A**, aniline, and $Zn(NTf_2)_2$ under similar conditions did not result in the formation of the respective cage. Reaction of subcomponent **B**, *p*-toluidine, and $Zn(NTf_2)_2$ under similar conditions did not result in the formation of the respective cage.

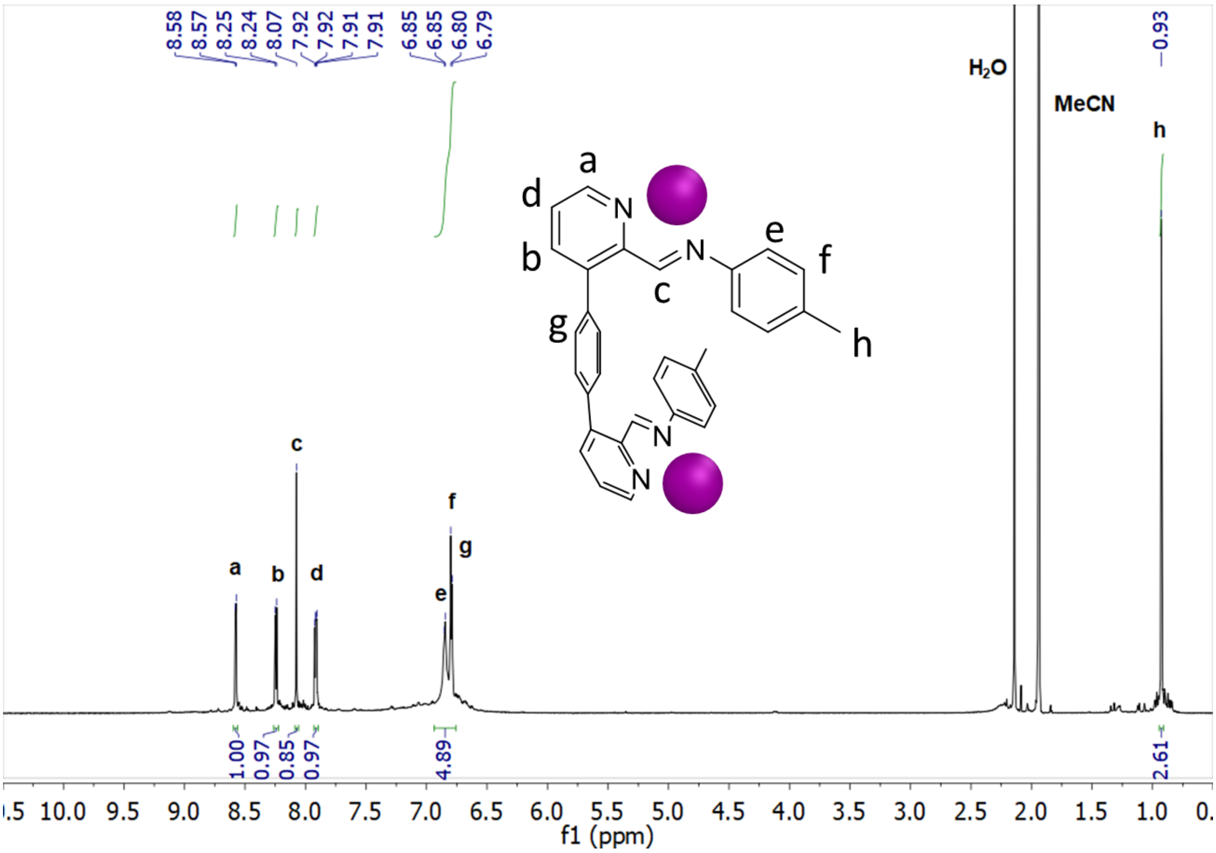

*Figure S11: ^{1}H NMR spectrum of **1**·(NTf2)16 (700 MHz, CD_3CN, 298 K).*

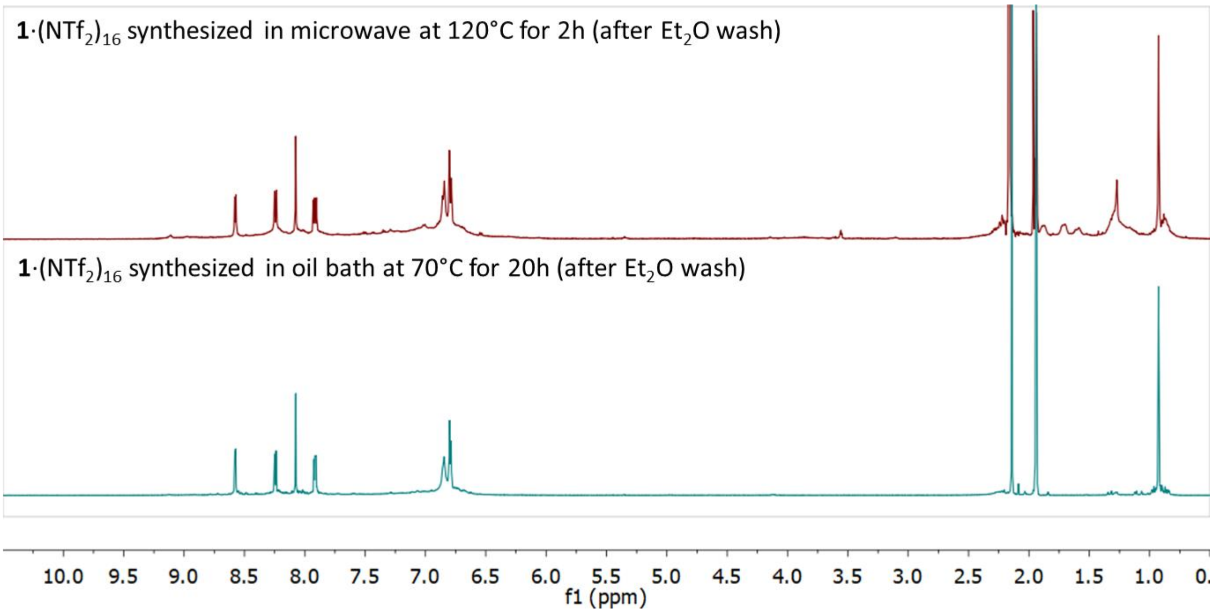

*Figure S12: ^{1}H NMR spectra of **1**·(NTf2)16 synthesized via oil bath heating (bottom) and microwave heating (top) (500 MHz, CD_3CN, 298 K).*

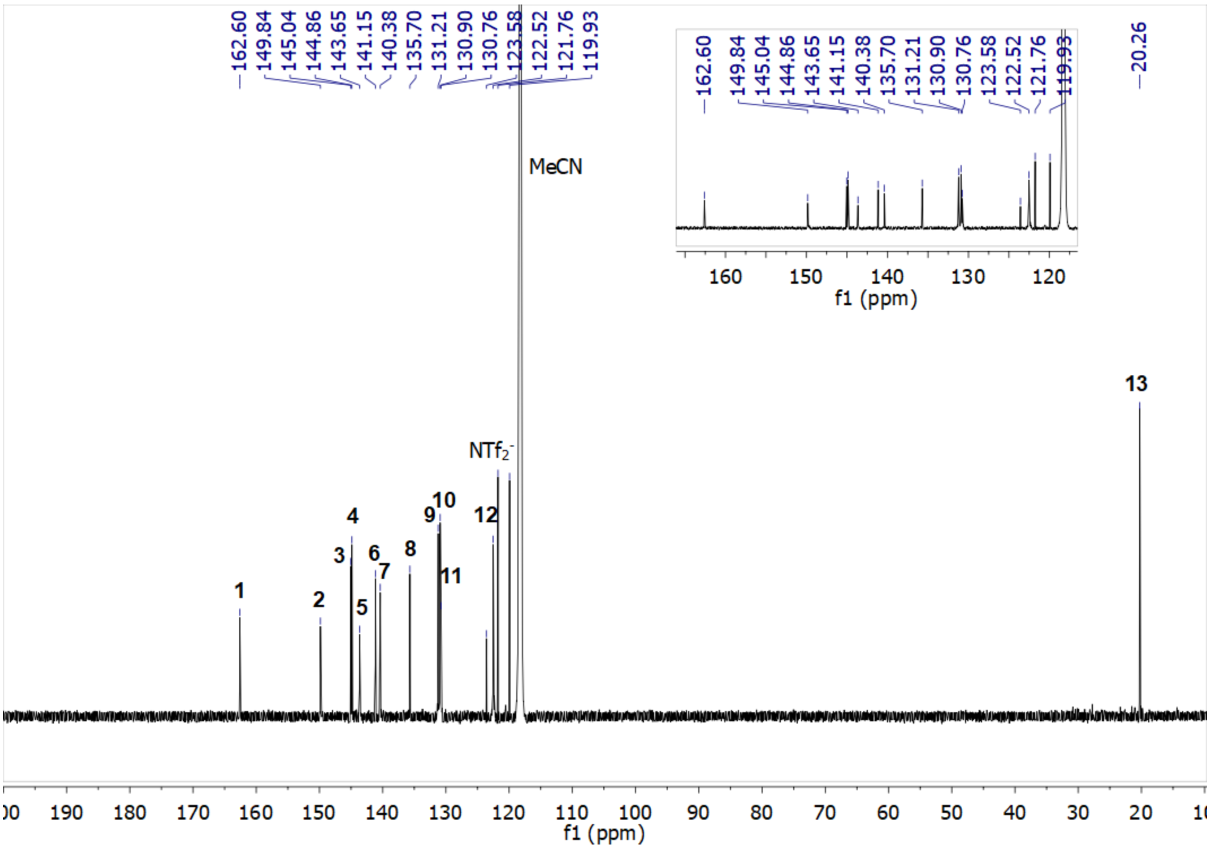

*Figure S13: ^{13}C NMR spectrum of **1**·$(NTf_2)_{16}$ (176 MHz, CD_3CN, 298 K).*

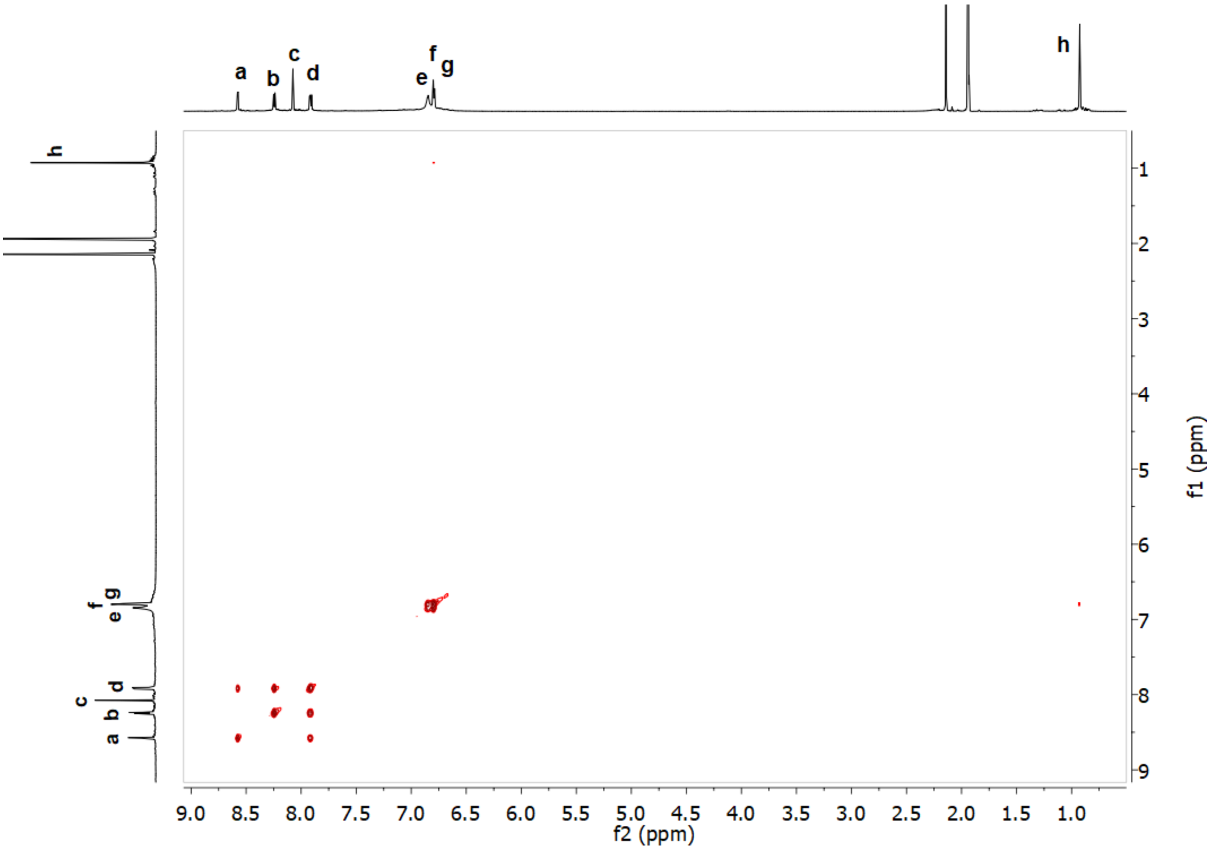

*Figure S14: ^{1}H-^{1}H DQF-COSY spectrum of **1**·$(NTf_2)_{16}$ (700 MHz, CD_3CN, 298 K).*

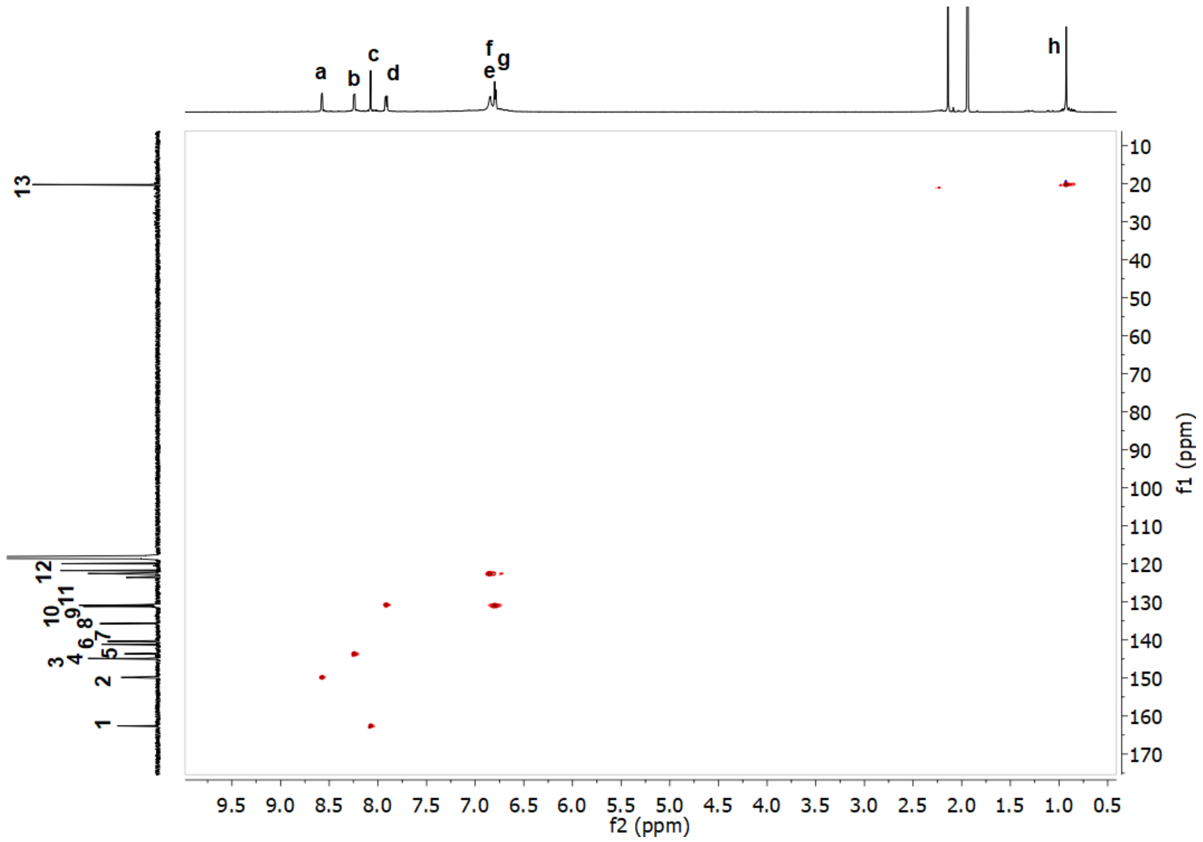

*Figure S15: ^{1}H-^{13}C HSQC spectrum of **1**·$(NTf_2)_{16}$ (700 MHz, 176 MHz, CD_3CN, 298 K).*

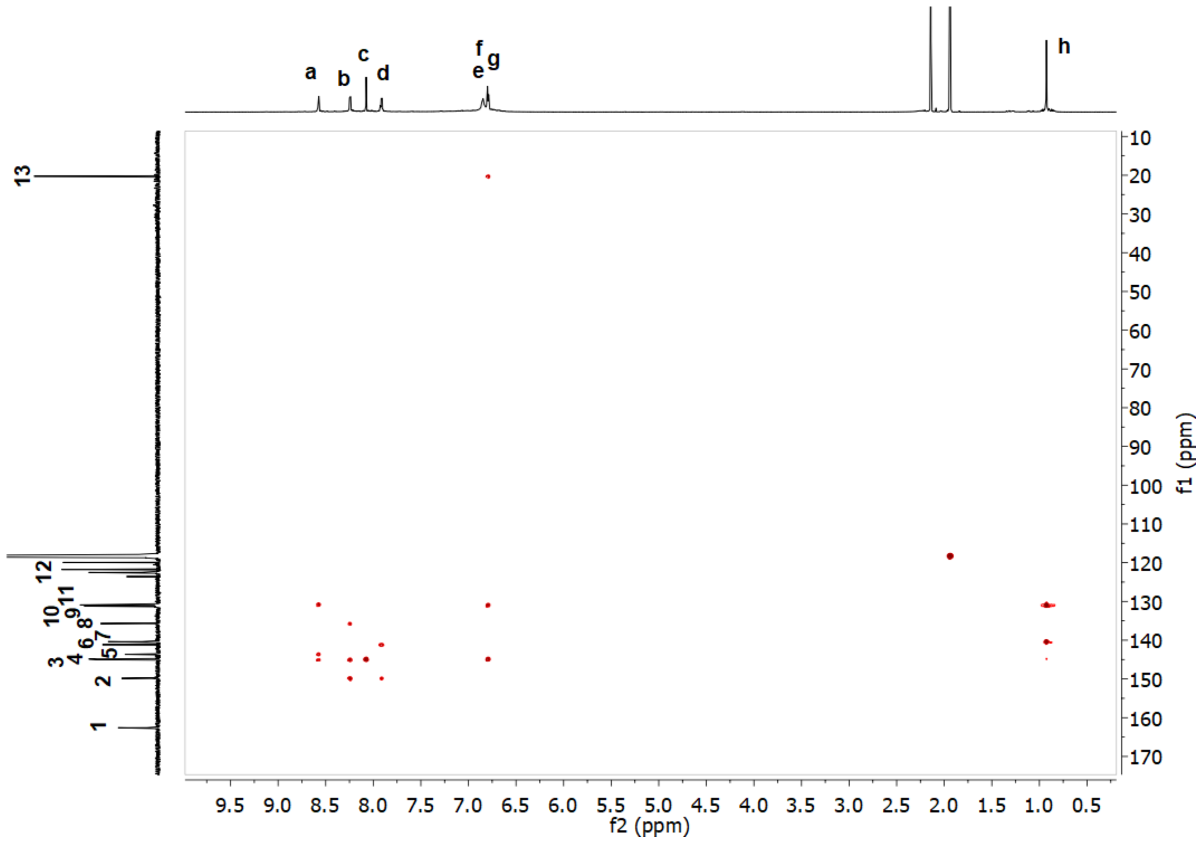

*Figure S16: ^{1}H-^{13}C HMBC spectrum of **1**·$(NTf_2)_{16}$ (700 MHz, 176 MHz, CD_3CN, 298 K).*

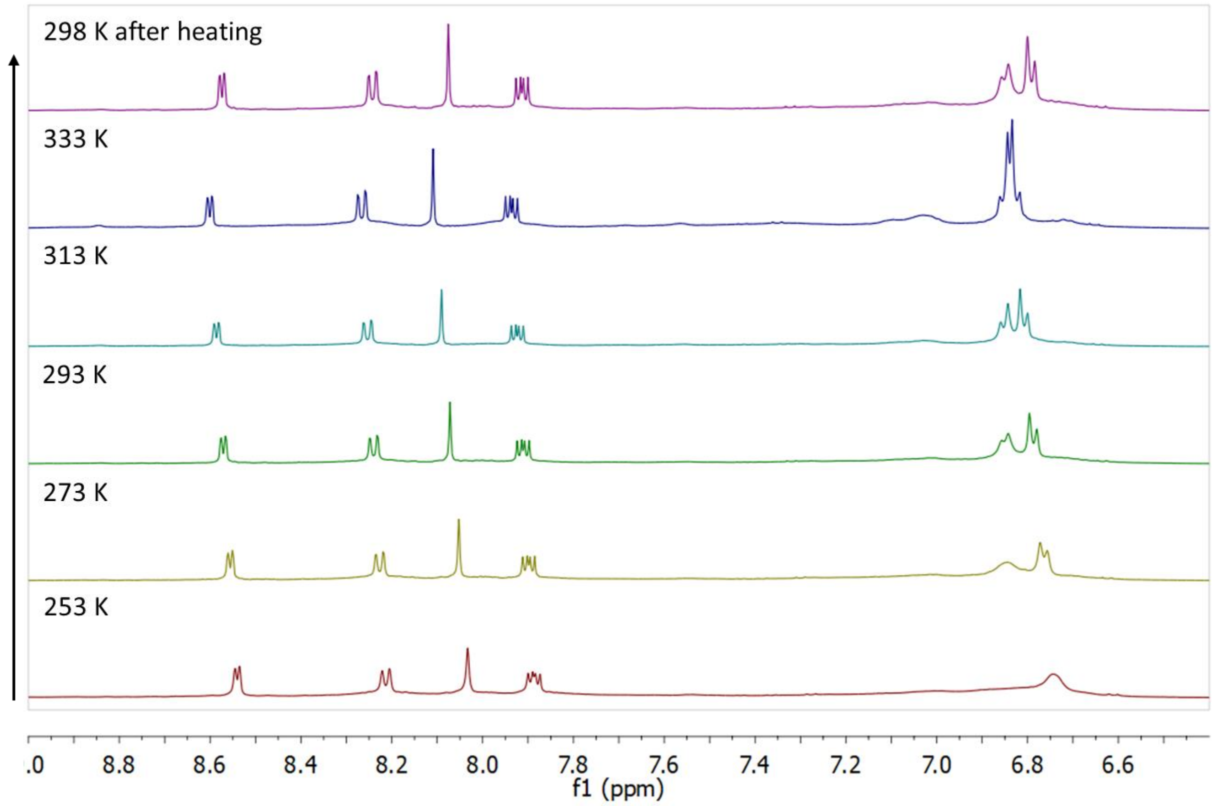

*Figure S17: Variable-temperature ^{1}H NMR spectra of **1**·(NTf$_2$)$_{16}$ (500 MHz, CD$_3$CN). A control measurement at 298 K after heating confirmed that heating did not affect the cage structure.*

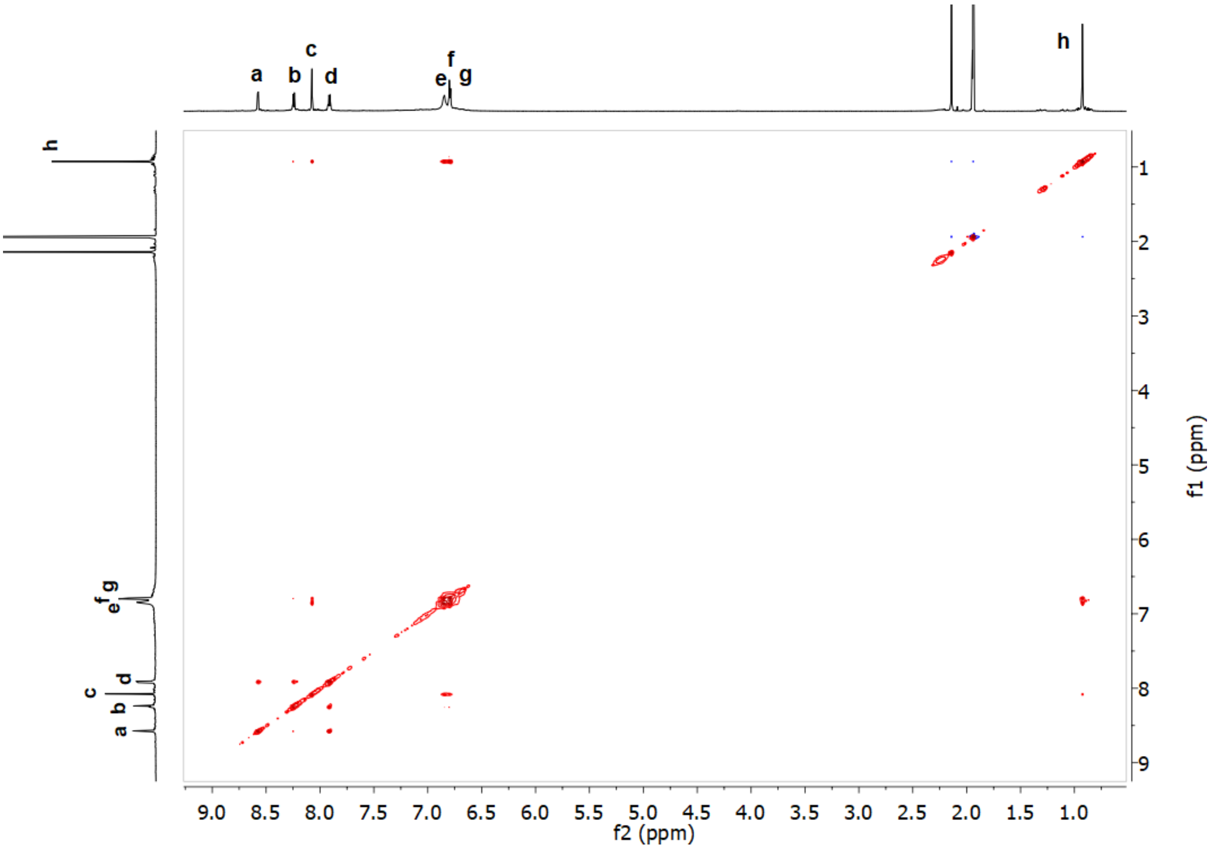

*Figure S18: ^{1}H-^{1}H NOESY spectrum of **1**·(NTf$_2$)$_{16}$ (700 MHz, CD$_3$CN, 298 K).*

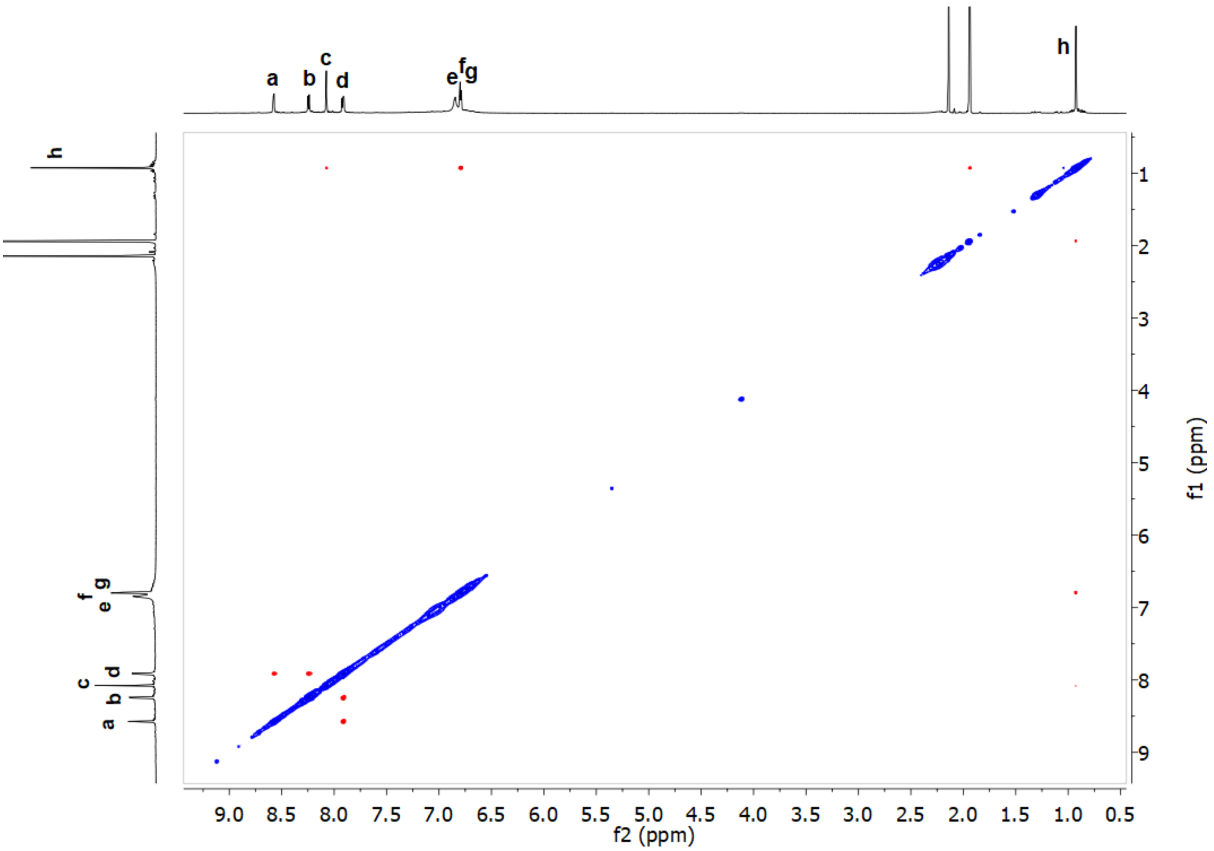

*Figure S19: ^{1}H-^{1}H ROESY spectrum of **1**·$(NTf_2)_{16}$ (700 MHz, CD_3CN, 298 K).*

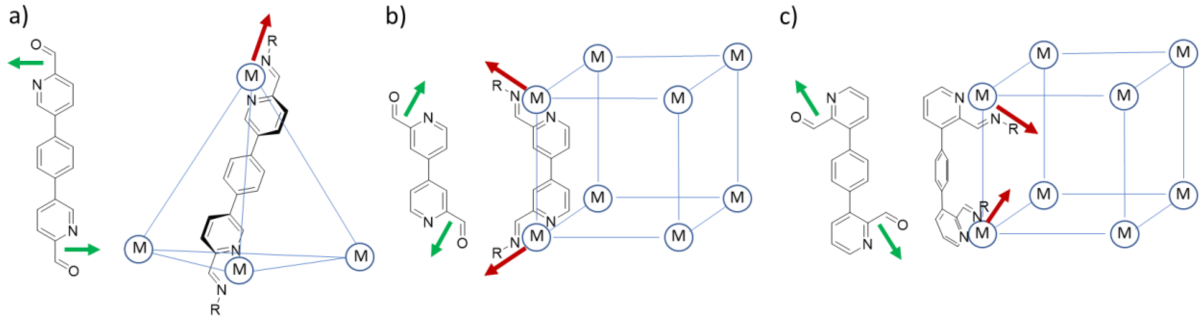

Figure S20: Effect of nitrogen and aldehyde position on the coordinative vector (green arrow) and the substituent orientation (red arrow). Typical aldehyde dialdehyde subcomponents and the respective imine cages are given in a) and b).[3,4] The new subcomponent motif is shown in c) leading to an altered coordinative vector and substituents pointing towards the faces.

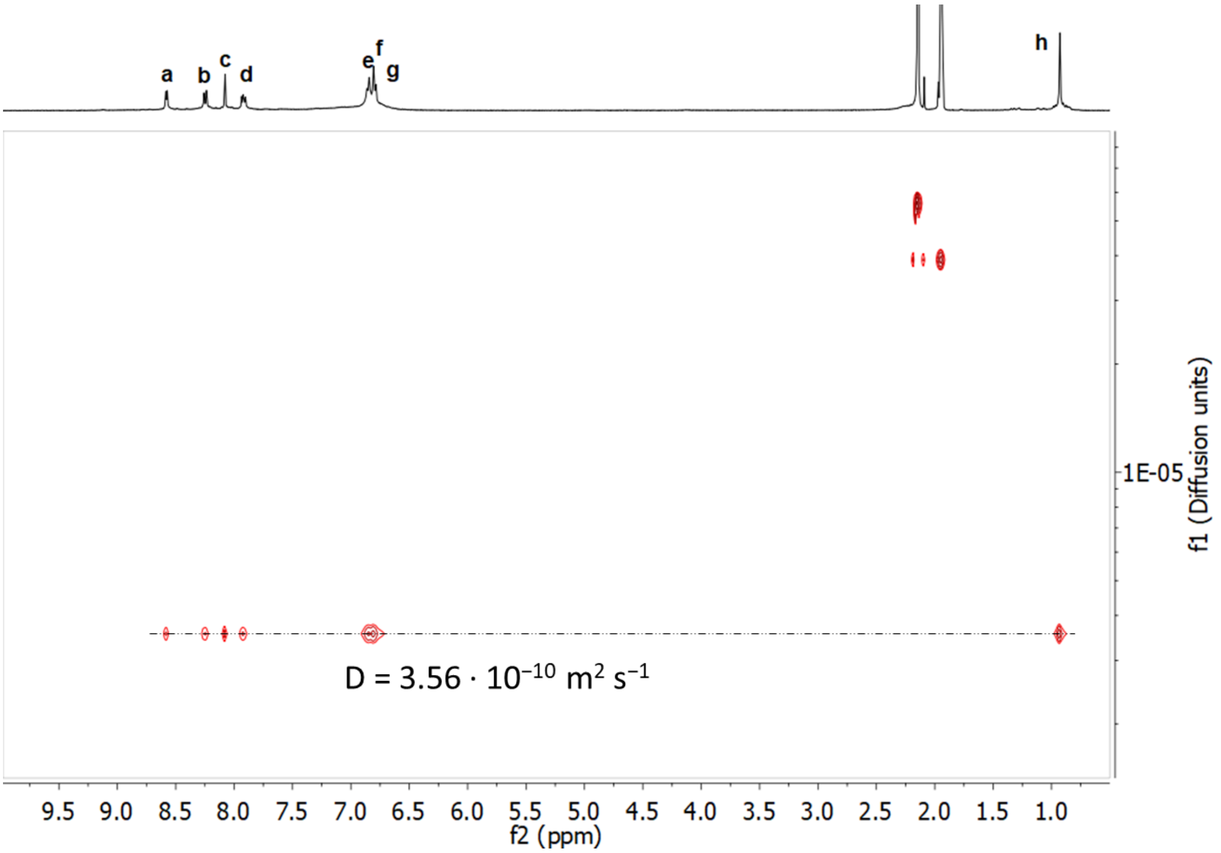

*Figure S21: ^{1}H DOSY spectrum of **1**·$(NTf_2)_{16}$ (400 MHz, CD_3CN, 298 K).*

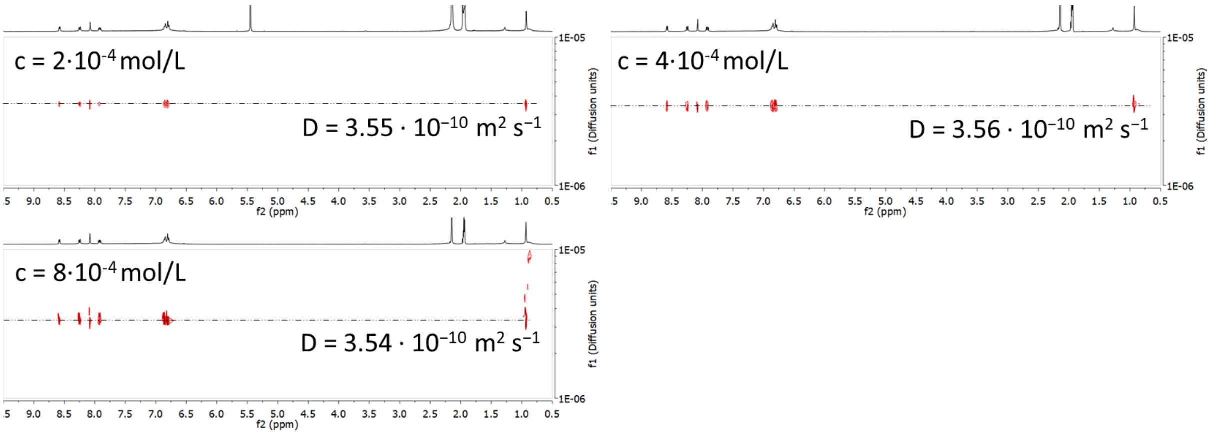

*Figure S22: ^{1}H DOSY spectrum of **1**·$(NTf_2)_{16}$ at different concentrations (0.2 to 0.8 mM) (400 MHz, CD_3CN, 298 K).*

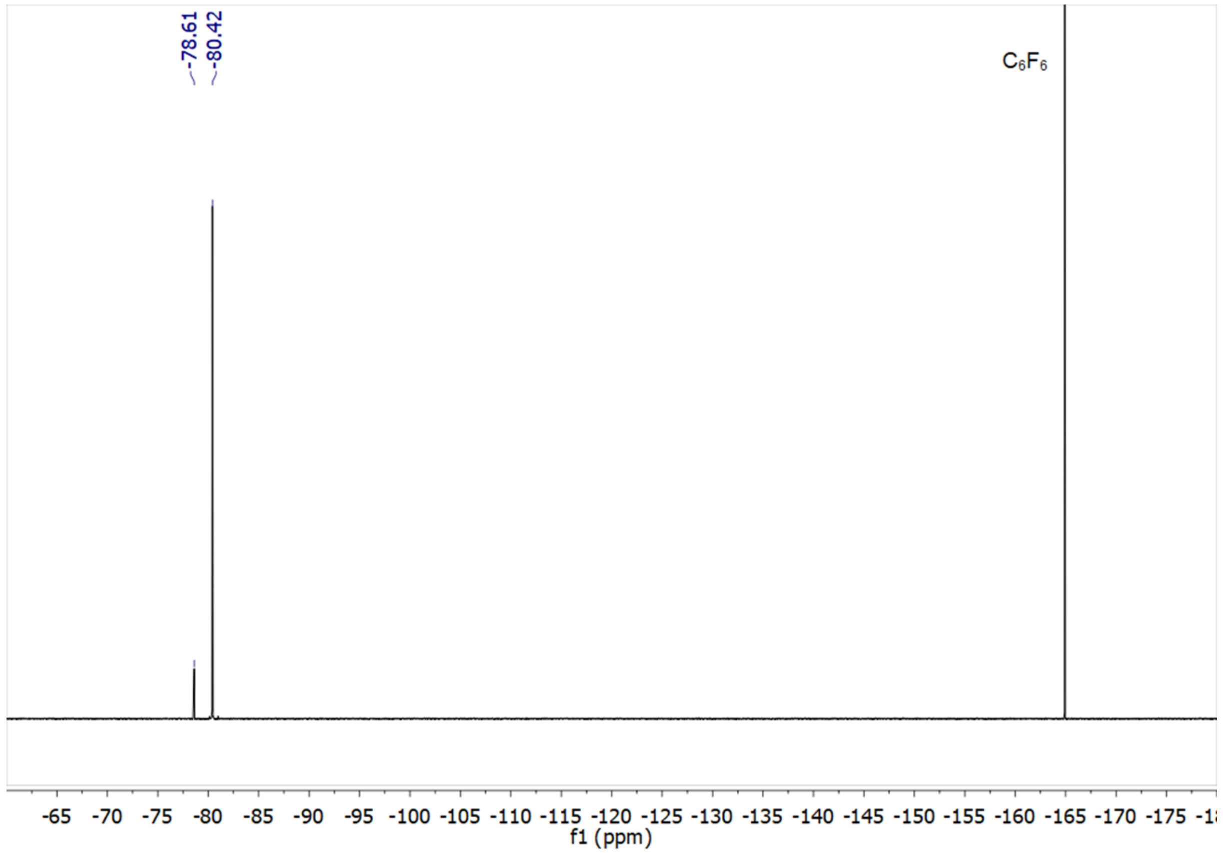

*Figure S23: ^{19}F NMR spectrum of **1**·$(NTf_2)_{16}$ (376 MHz, CD_3CN, 298 K). Please note that T1 differs notably between the signal at -78.61 ppm (T1 = 0.70 s) and -80.42 (T1 = 1.68 s).*

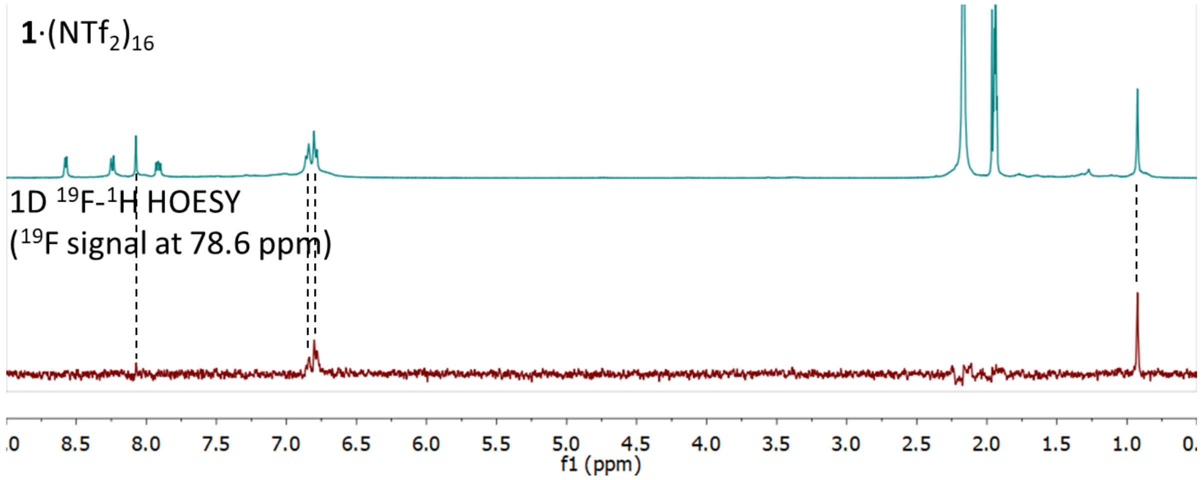

*Figure S24: 1D ^{19}F-^{1}H NMR spectrum of **1**·$(NTf_2)_{16}$ with ^{19}F signal at 78.6 ppm (slow exchange) (376 MHz, CD_3CN, 298 K).*

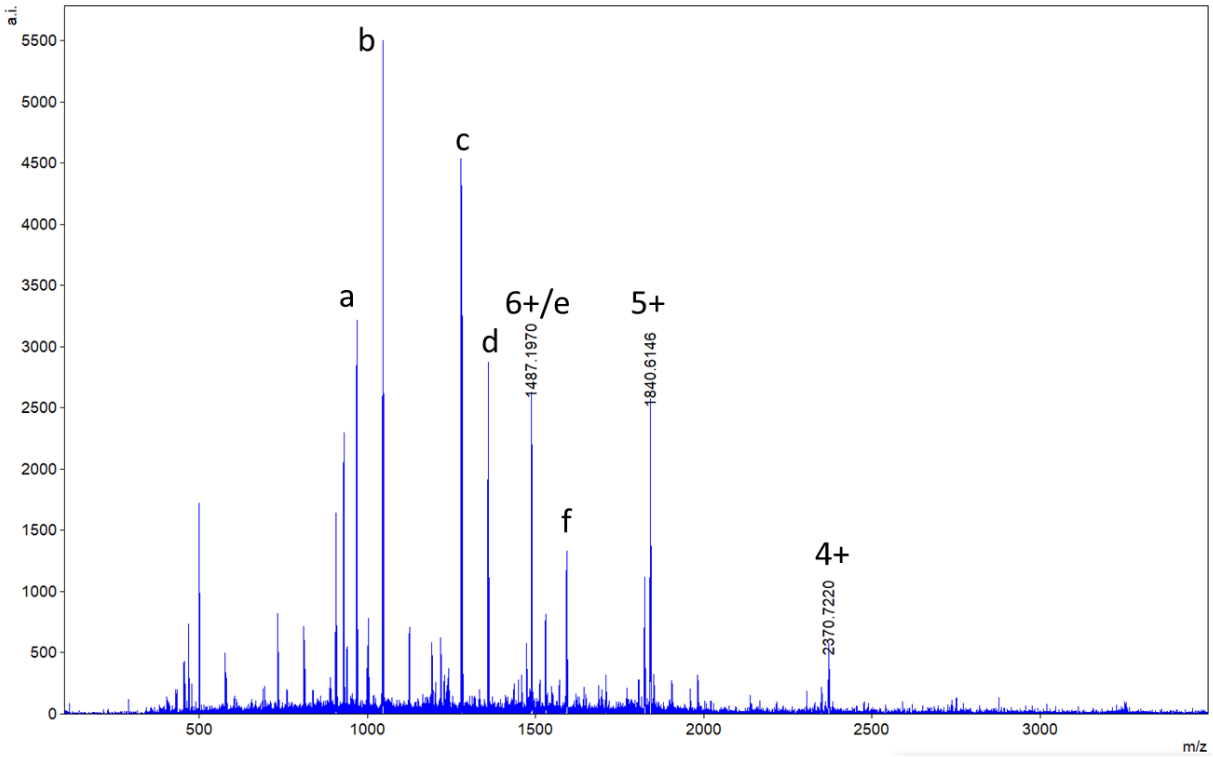

*Figure S25: High resolution ESI-MS spectrum of **1**·$(NTf_2)_{16}$. Please note, that the following fragments have been observed: a) $[Zn_3L_4{\cdot}(NTf_2)_3]^{3+}$, b) $[Zn_2L_3{\cdot}(NTf_2)_2]^{2+}$, c) $[Zn_1L_2{\cdot}(NTf_2)_1]^{1+}$, d) $[Zn_3L_3{\cdot}(NTf_2)_2]^{2+}$, e) $[Zn_4L_6{\cdot}(NTf_2)_5]^{3+}$, f) $[Zn_3L_4{\cdot}(NTf_2)_4]^{2+}$.*

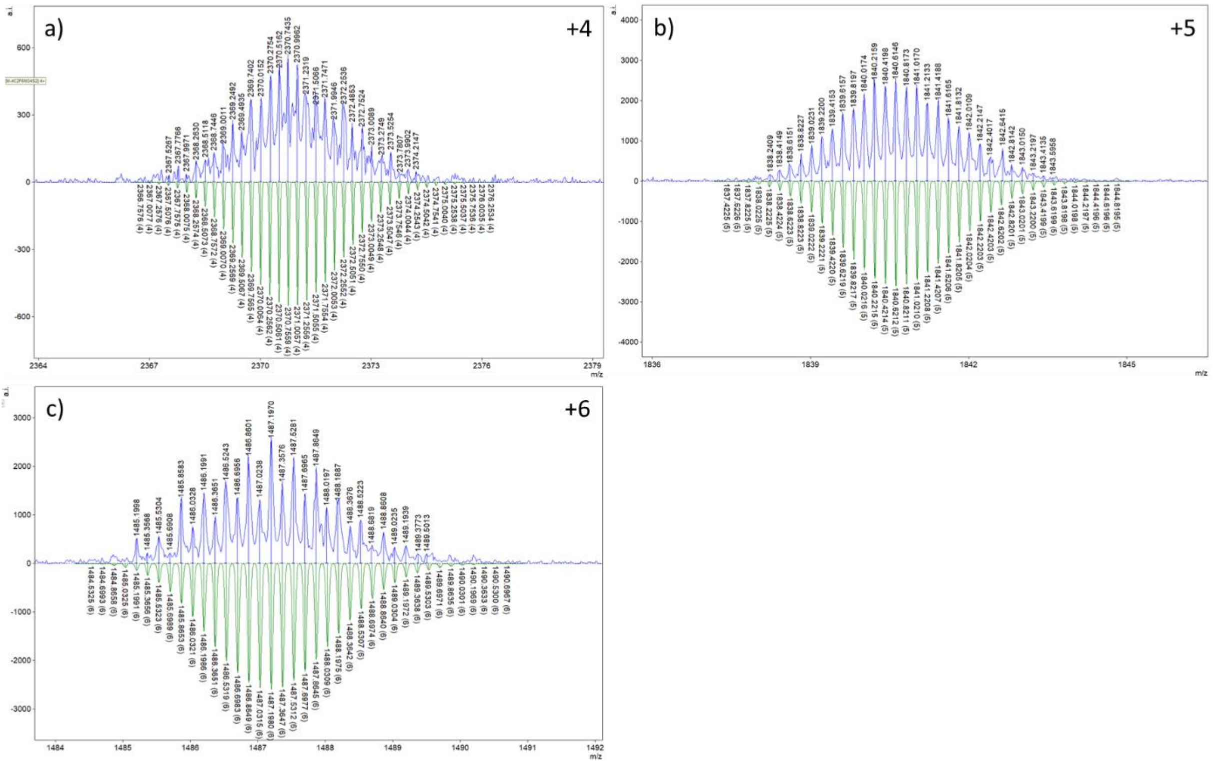

*Figure S26: Zoom into high resolution ESI-MS signals of **1**·$(NTf_2)_{16}$. Experimental (blue) and calculated (green) signals for a) $[\mathbf{1}{\cdot}(NTf_2)_{12}]^{4+}$ b) $[\mathbf{1}{\cdot}(NTf_2)_{11}]^{5+}$ and c) $[\mathbf{1}{\cdot}(NTf_2)_{10}]^{6+}$. Please note the overlap of the $[\mathbf{1}{\cdot}(NTf_2)_{10}]^{6+}$ signal with the $[Zn_4L_6{\cdot}(NTf_2)_5]^{3+}$ fragment.*

$\mathbf{2}\cdot(NTf_2)_{16}$

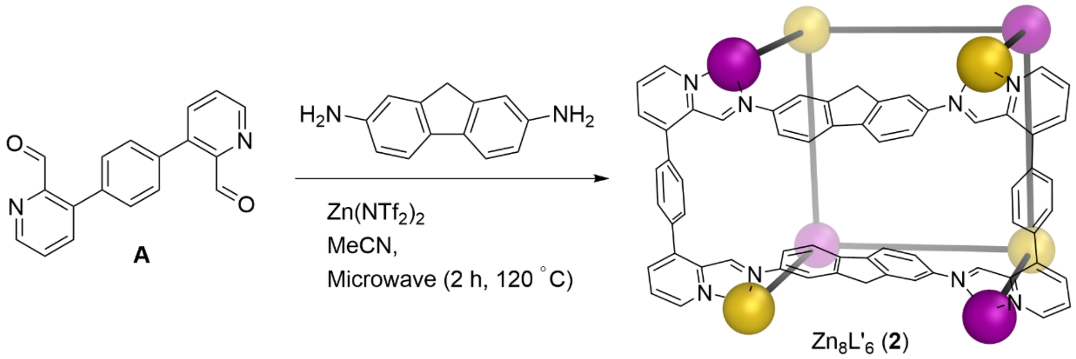

Subcomponent **A** (15.0 mg, 52.0 µmol, 1.5 eq), 2,7-diaminofluorene (10.2 mg, 52.0 µmol, 1.5 eq), and $Zn(NTf_2)_2$ (23.9 mg, 38.2 µmol, 1.1 eq) were added to in 5 mL freshly distilled MeCN in a microwave reactor and heated for 2 h at 120°C. After cooling, the reaction mixture was filtered over a glass fibre plug, concentrated to 1 mL and precipitated with 50 mL Et_2O. The red precipitate was collected via centrifugation, washed two times with 50 mL Et_2O, and dried in vacuo. $\mathbf{2}\cdot(NTf_2)_{16}$ was obtained as a red solid (m = 38.0 mg, 84%).

^{1}H-NMR (500 MHz, CD_3CN, 298 K): δ = 8.41 (broad d, 1H, a); 8.37 (s, 1H, b); 8.26 (d, 1H, c); 8.15 (broad d, 1H, d); 7.96 (broad s, 1H, e); 7.87 (broad dd, 1H, f); 7.28 (broad d, ^3J = 7.6 Hz, 1H, g); 6.37 (broad d, ^3J = 7.3 Hz, 1H, h); 4.98 (broad s, 1H, i) ; 4.31, 4.19 (2 broad d, ^2J = 22.0 Hz, 1H, j). Please note that the broadness of the peaks did not allow determination of the coupling constants for most peaks.**^{13}C-NMR** (126 MHz, CD_3CN, 298 K): δ = 163.6, 148.3, 147.6, 146.1, 144.3, 144.1, 142.8, 142.6, 135.8, 133.9, 130.4, 129.7, 124.2, 122.5, 121.3, 120.8 (q, J = 321.3 Hz, NTf_2), 38.11. **^{19}F-NMR** (376 MHz, CD_3CN, 298 K): δ = -80.4. **ESI-HRMS**: ([$\mathbf{2}\cdot(NTf_2)_{16}$] = (C62H40N8)6Zn8(C2F6NO4S2)16 m/z = 874.0 [$\mathbf{2}\cdot(NTf_2)_7$]$^{9+}$ (calc. 874.0), 1018.2 [$\mathbf{2}\cdot(NTf_2)_8$]$^{8+}$ (calc. 1018.3), 1203.8 [$\mathbf{2}\cdot(NTf_2)_{19}$]$^{7+}$ (calc. 1203.8), 1451.1 [$\mathbf{2}\cdot(NTf_2)_{10}$]$^{6+}$ (calc. 1451.1), 1797.5 [$\mathbf{2}\cdot(NTf_2)_{11}$]$^{5+}$ (calc. 1797.4).

Reaction of subcomponent **A**, 2,7-diaminofluorene, and $Zn(NTf_2)_2$ via oil bath heating at 70°C for 24 h/72 h also resulted in the formation of cage **2**, although the signals in the ^{1}H NMR spectra were slightly broader. Attempts to form a single-bridged Zn_8L_{12} cube from subcomponent **B** and other longer anilines, including 4-phenylaniline or 4-(4-methylphenyl)aniline), were unsuccessful. In some cases, sharp signals were observed in the NMR spectra of the reaction mixture, but high-resolution mass spectrometry only revealed fragments up to triangular Zn_3L_3 species, with no signals corresponding to a cubic structure. Furthermore, DOSY experiments confirmed the formation of species with smaller hydrodynamic radii than the targeted cubic structures.

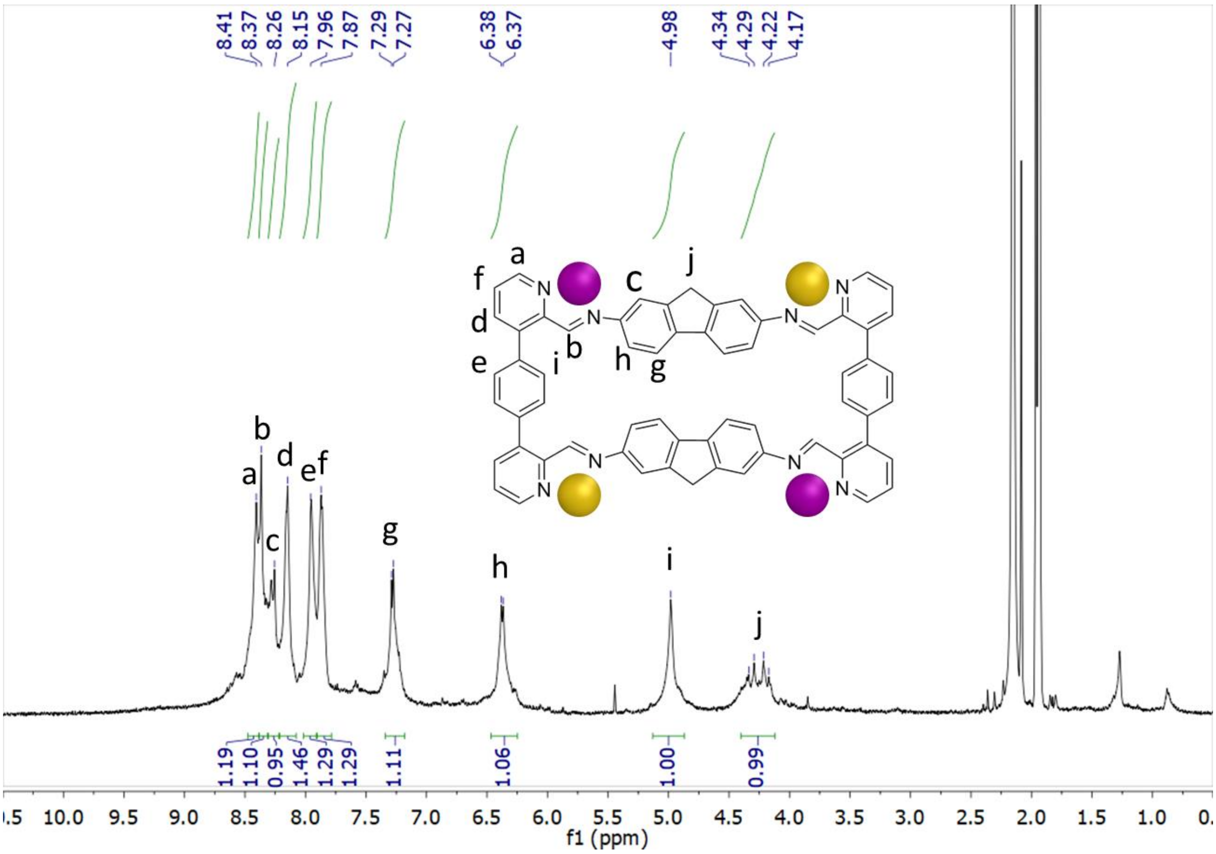

*Figure S27: ^{1}H NMR spectrum of **2**·(NTf$_2$)$_{16}$ (500 MHz, CD_3CN, 298 K). The proximity of the protons on the double-bridged edges of the fluorene and phenyl bridge results in a broadening of the proton signals due to limited rotational freedom.*

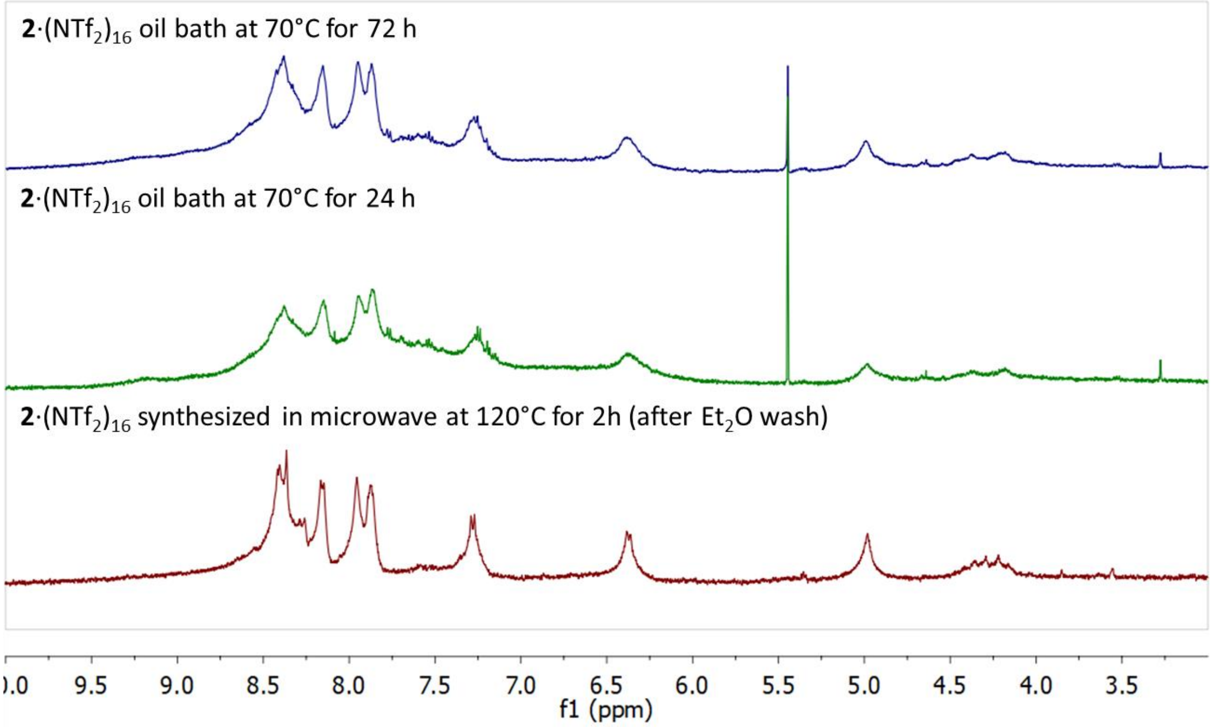

*Figure S28: ^{1}H NMR spectra of **2**·(NTf$_2$)$_{16}$ synthesized via microwave heating (bottom) and oil bath heating for 24 h (middle) and 72 h (top) (500 MHz, CD_3CN, 298 K).*

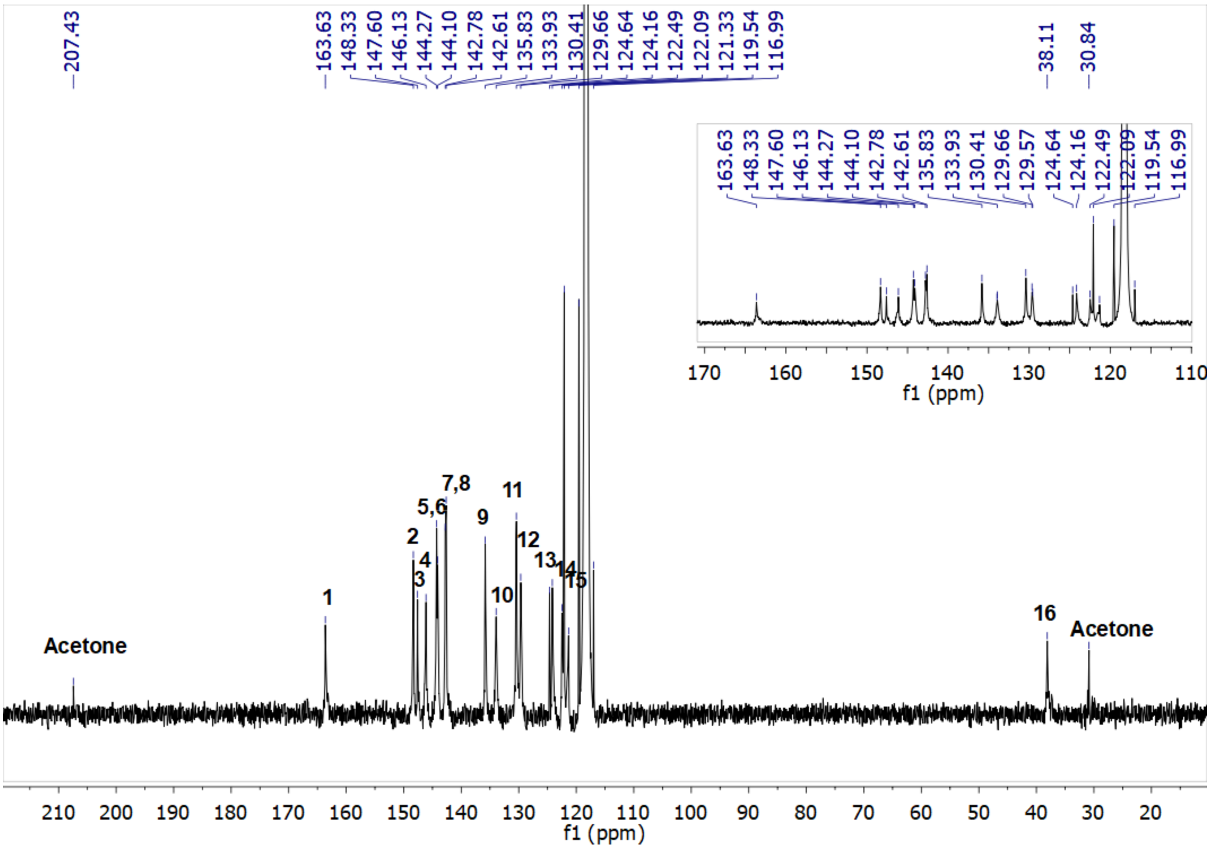

*Figure S29: ^{13}C UDEFT-NMR spectrum of **2**·$(NTf_2)_{16}$ (126 MHz, CD_3CN, 298 K).*

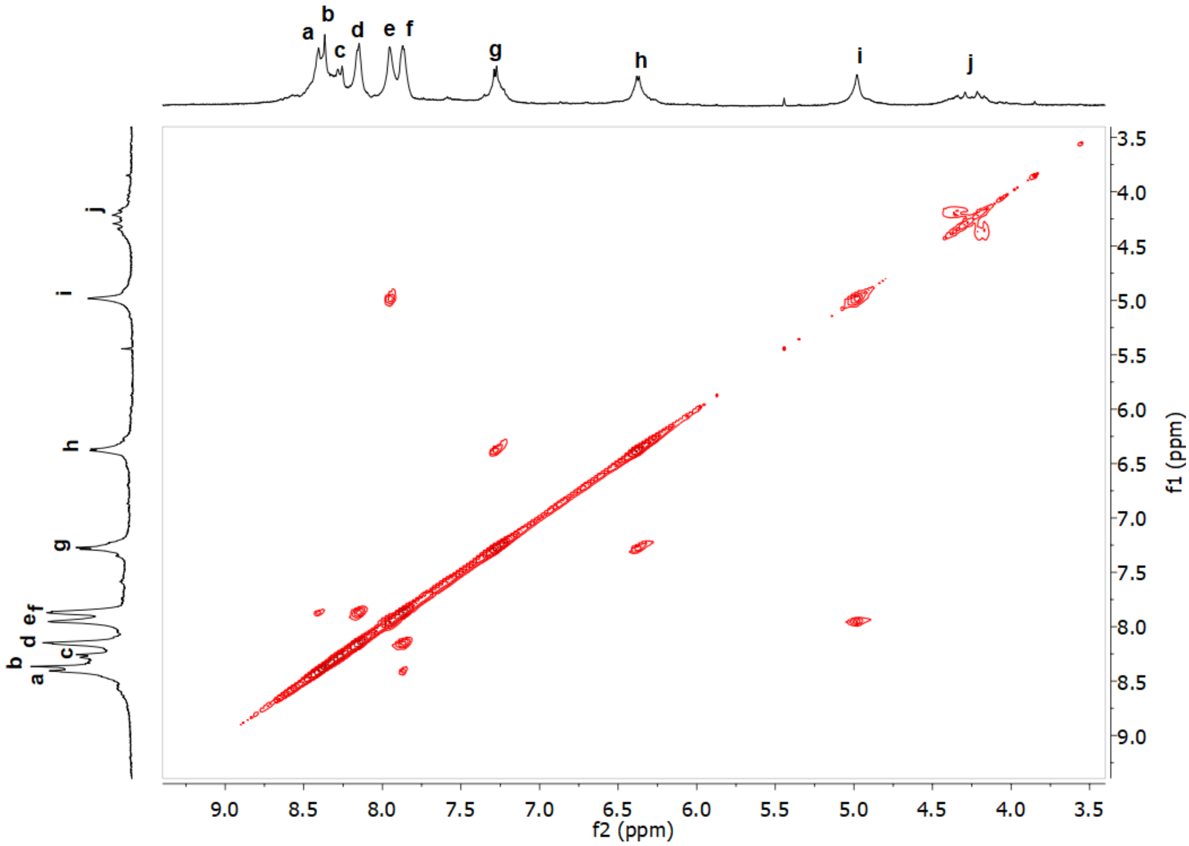

*Figure S30: ^1H-^1H Clip-COSY spectrum of **2**·$(NTf_2)_{16}$ (500 MHz, CD_3CN, 298 K).*

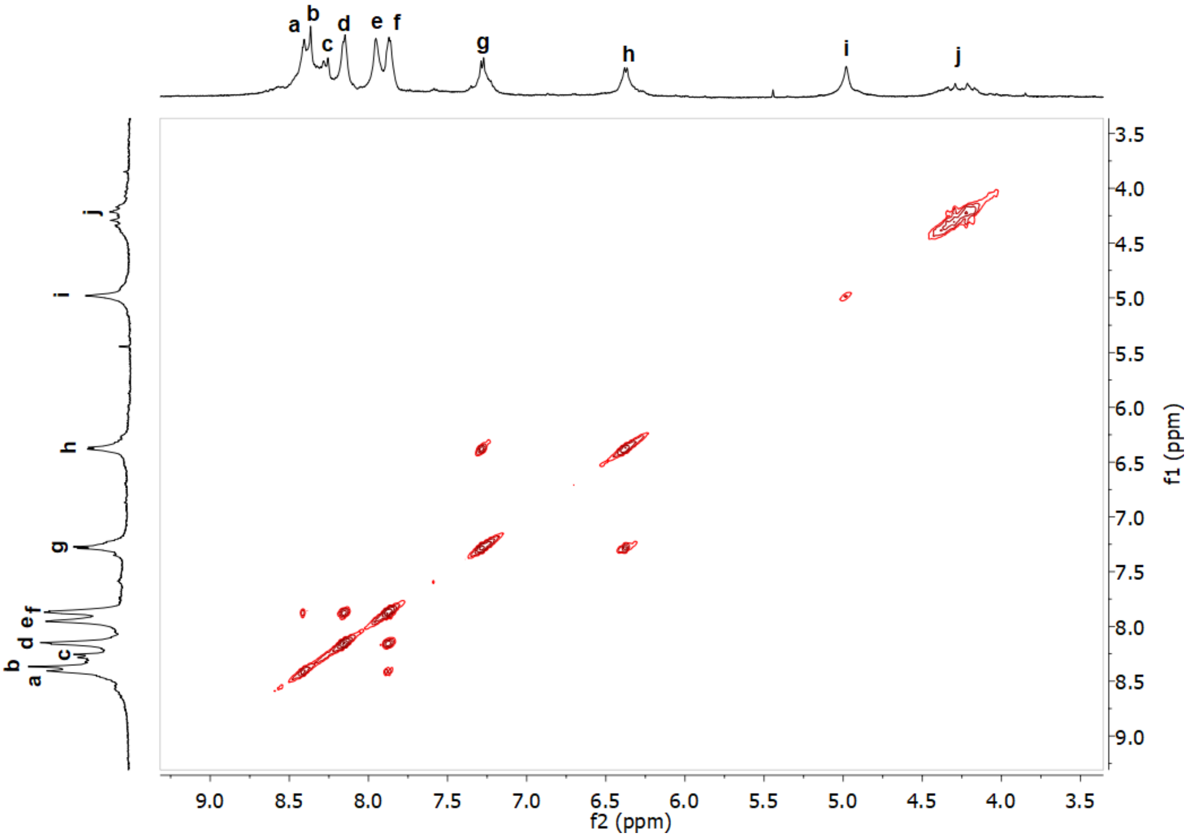

*Figure S31: ^{1}H-^{1}H COSY spectrum of **2**·$(NTf_2)_{16}$ (500 MHz, CD_3CN, 298 K).*

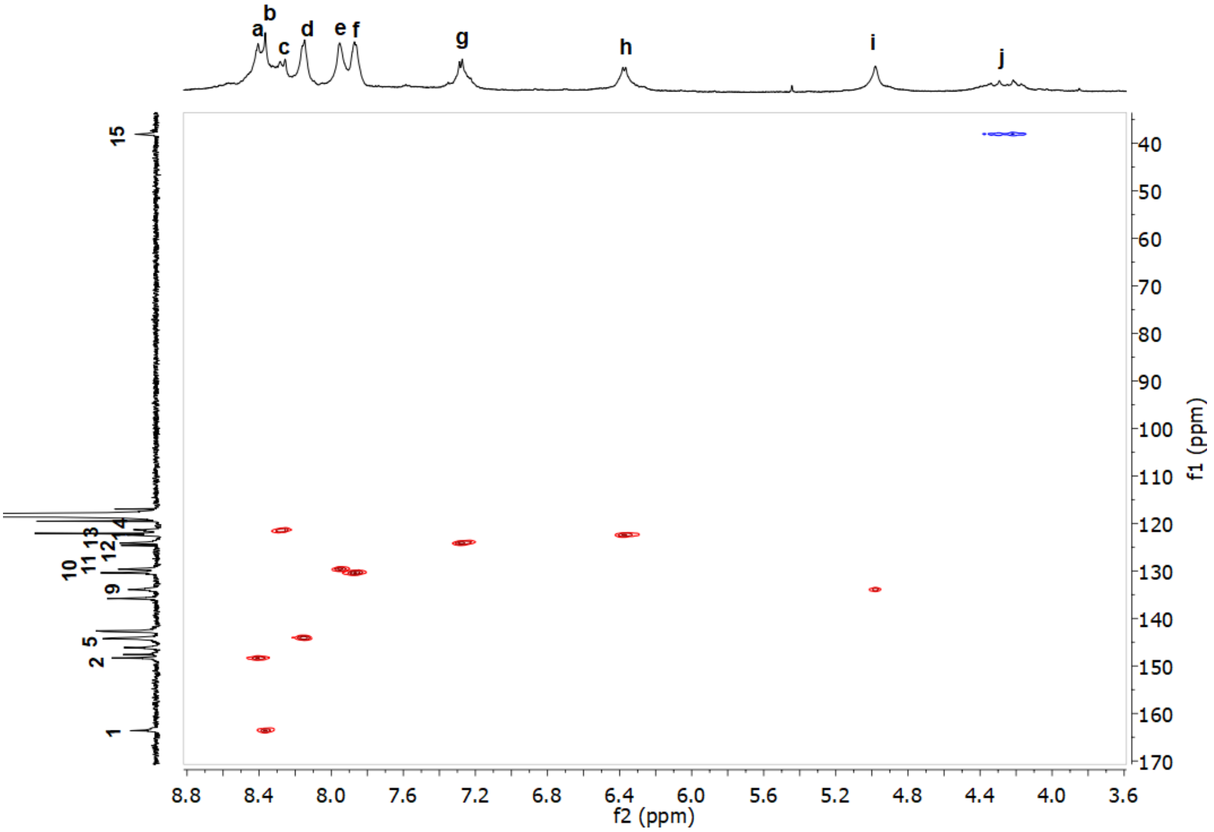

*Figure S32: ^{1}H-^{13}C HSQC spectrum of **2**·$(NTf_2)_{16}$ (500 MHz, 126 MHz, CD_3CN, 298 K).*

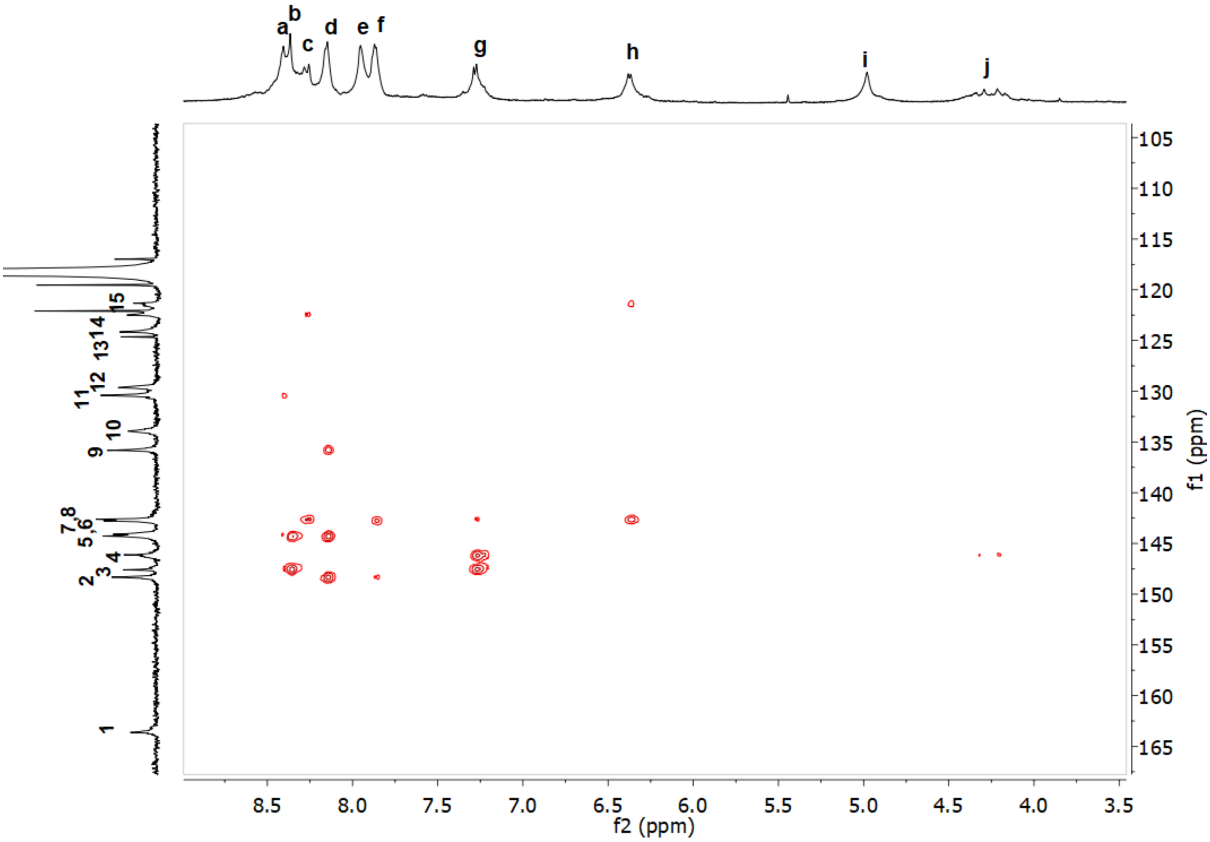

*Figure S33: ^{1}H-^{13}C HMBC spectrum of **2**·$(NTf_2)_{16}$ (500 MHz, 126 MHz, CD_3CN, 298 K).*

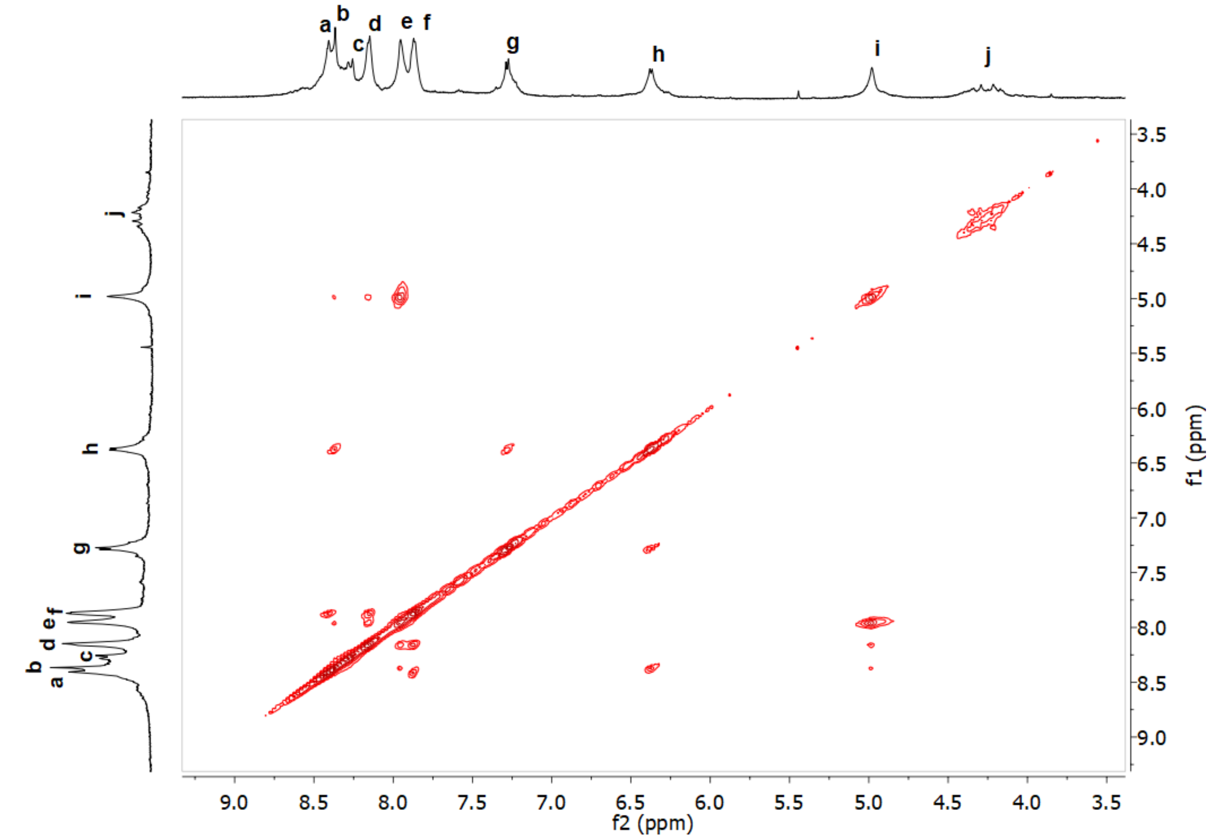

*Figure S34: ^{1}H-^{1}H NOESY spectrum of **2**·$(NTf_2)_{16}$ (500 MHz, CD_3CN, 298 K).*

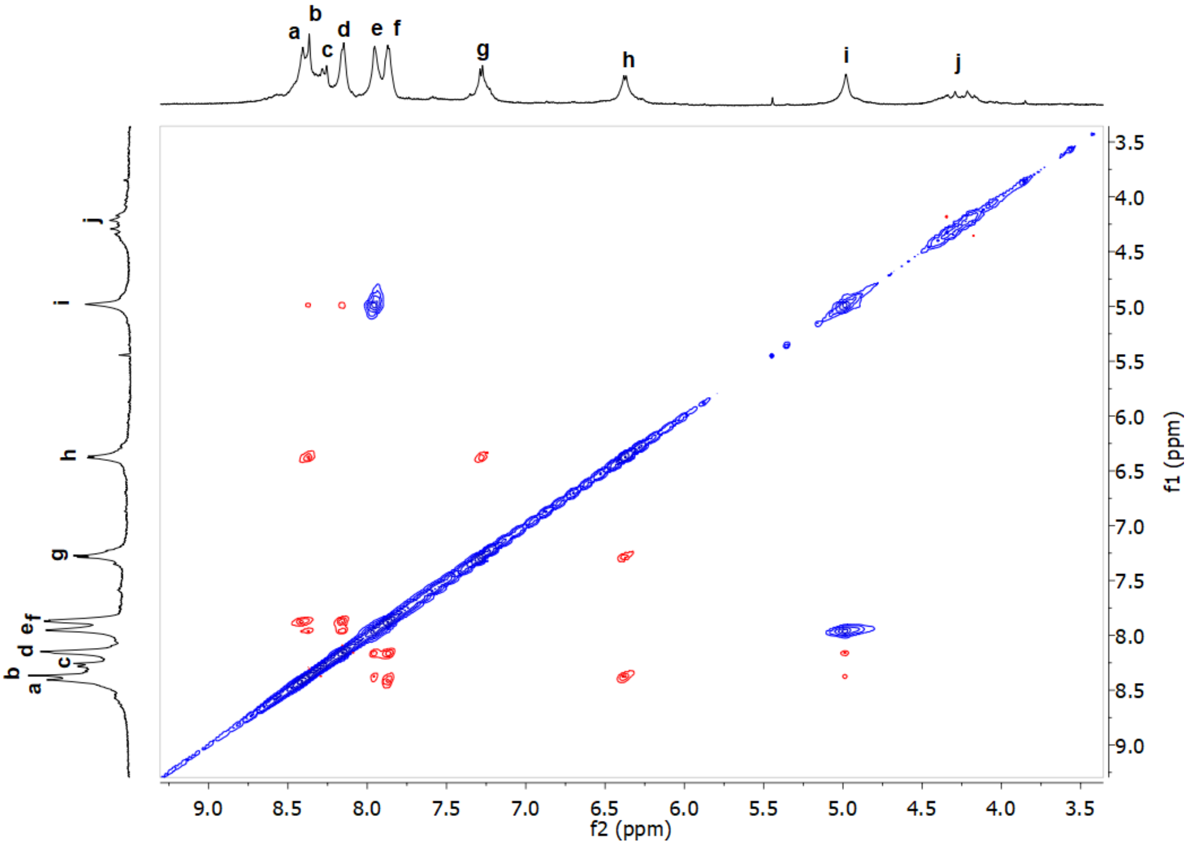

*Figure S35: 1H-1H ROESY spectrum of **2**·$(NTf_2)_{16}$ (500 MHz, CD_3CN, 298 K).*

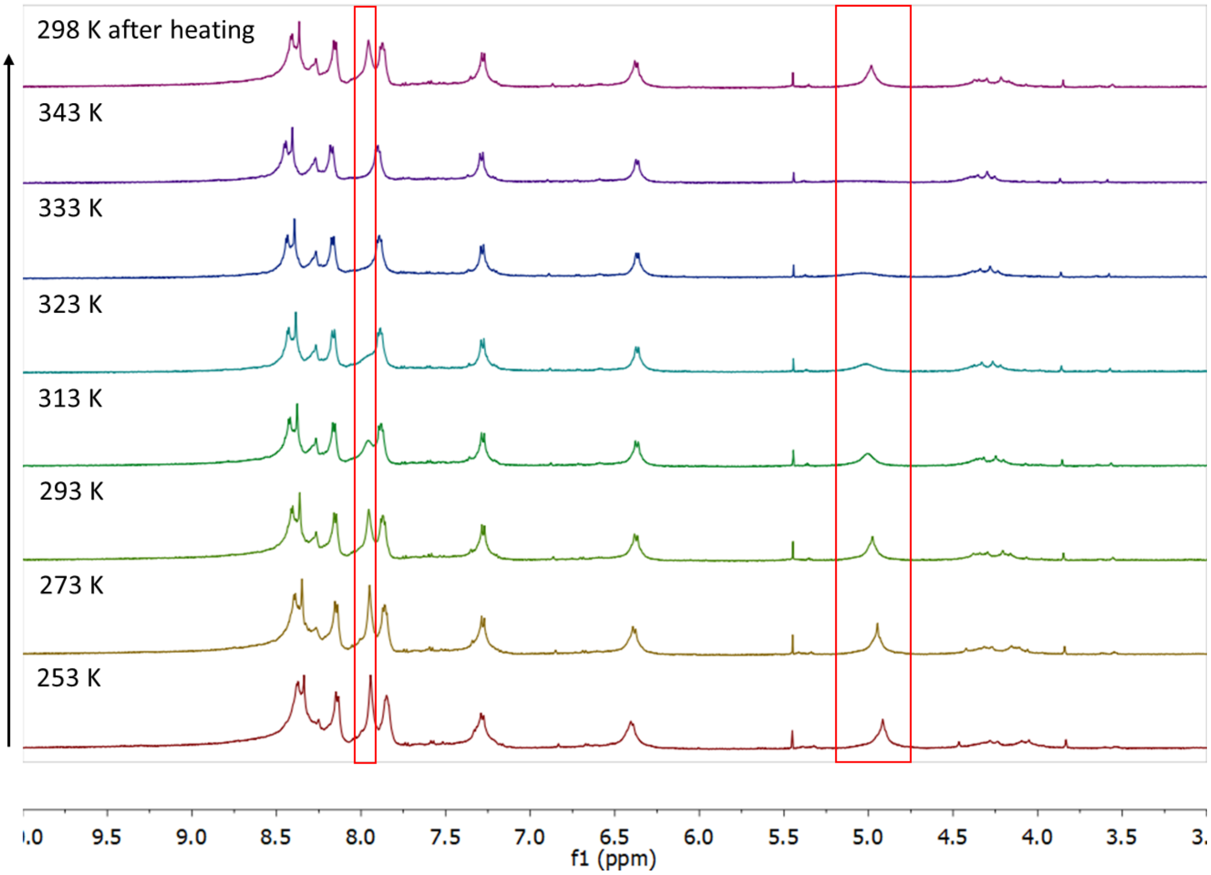

*Figure S36: Variable-temperature ^{1}H NMR spectra of **2**·$(NTf_2)_{16}$ (500 MHz, CD_3CN). Upon temperature increase, the increased rotational freedom results in significant broadening of the signals of protons e and i and a shift towards each other. However, even at 343 K coalescence was not observed. A control measurement at 298 K after heating showed the reappearance of protons e and i.*

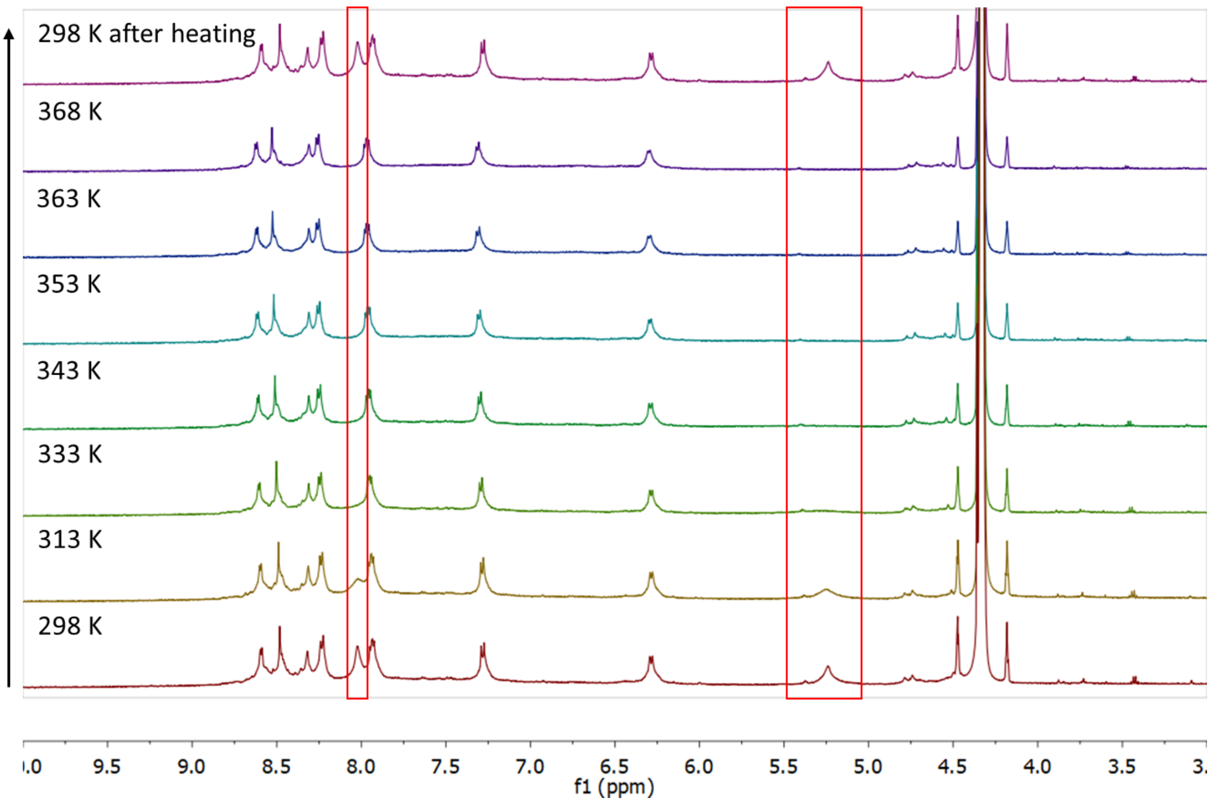

*Figure S37: Variable-temperature ^{1}H NMR spectra of **2**·$(NTf_2)_{16}$ (500 MHz, CD_3NO_2). Upon temperature increase, increased rotational freedom resulted in significant broadening of the signals of protons e and i and their shift towards each other. However, even at 368 K coalescence was not observed. A control measurement at 298 K after heating showed the reappearance of protons e and i.*

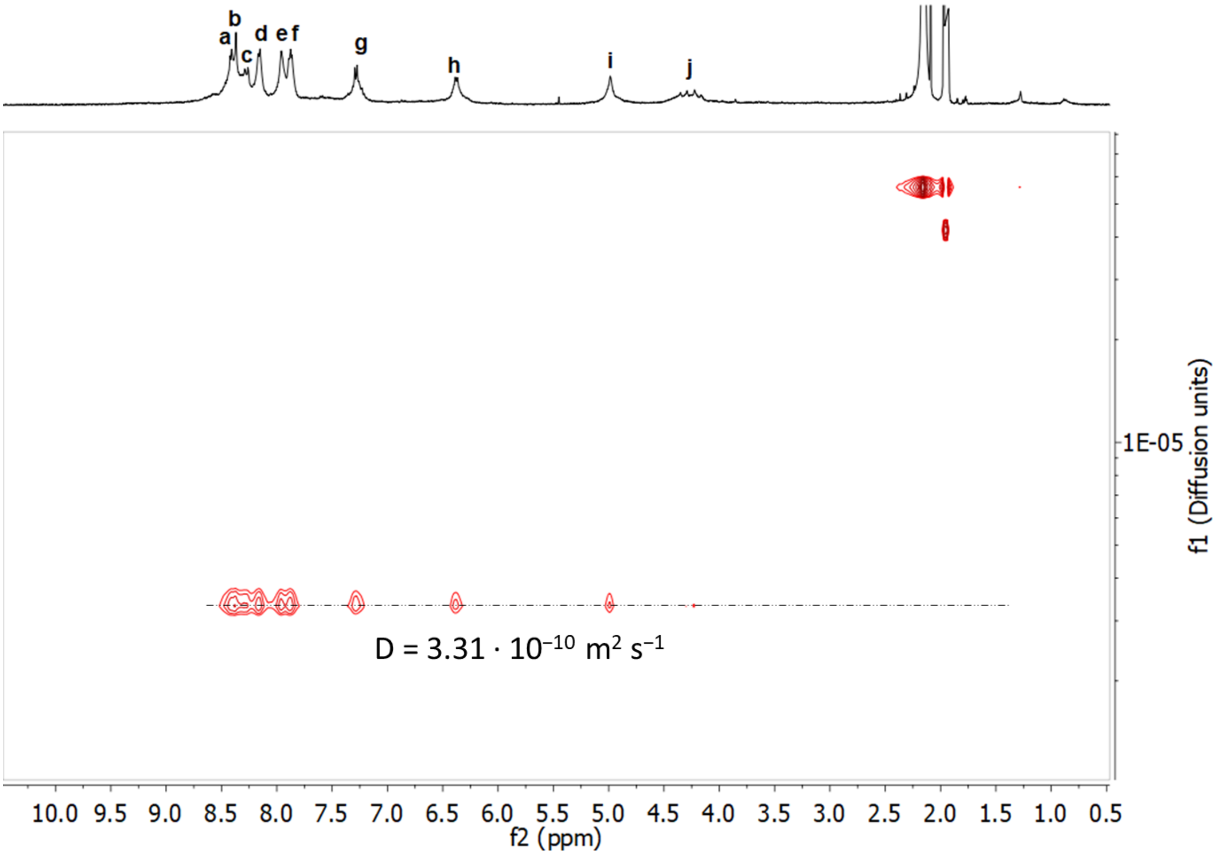

*Figure S38: ^{1}H DOSY spectrum of **2**·$(NTf_2)_{16}$ (400 MHz, CD_3CN, 298 K).*

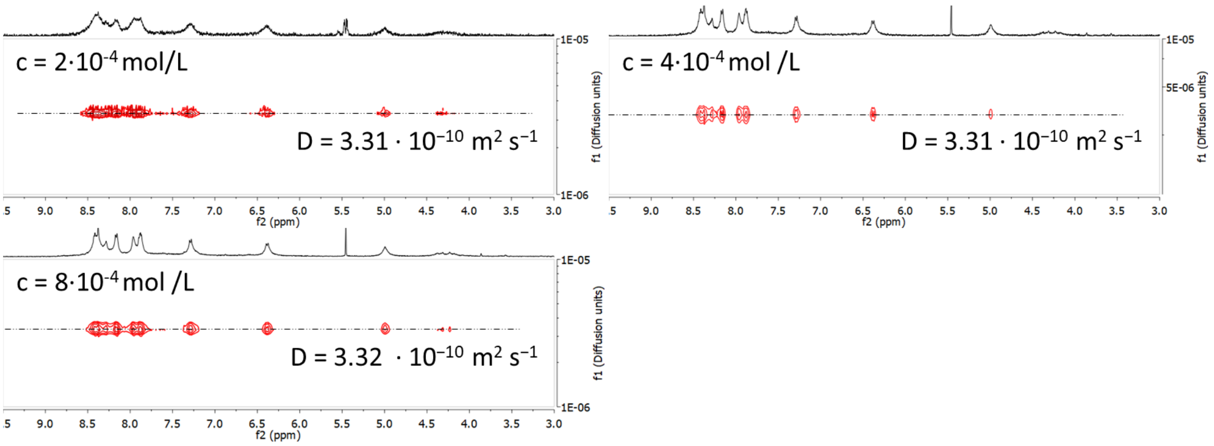

*Figure S39: ^{1}H DOSY spectrum of **2**·$(NTf_2)_{16}$ at different concentrations (400 MHz, CD_3CN, 298 K).*

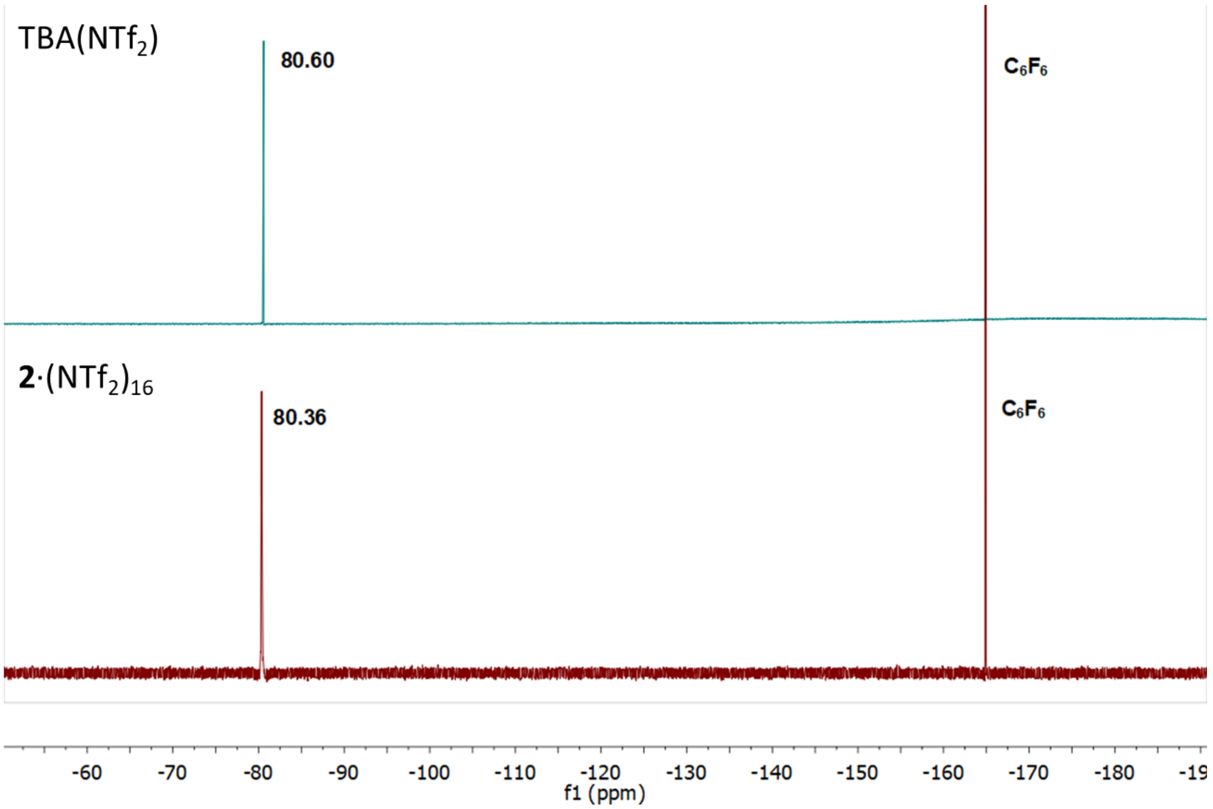

*Figure S40: ^{19}F NMR spectrum of **2**·(NTf$_2$)$_{16}$ and TBA(NTf$_2$) (376 MHz, CD$_3$CN, 298 K).*

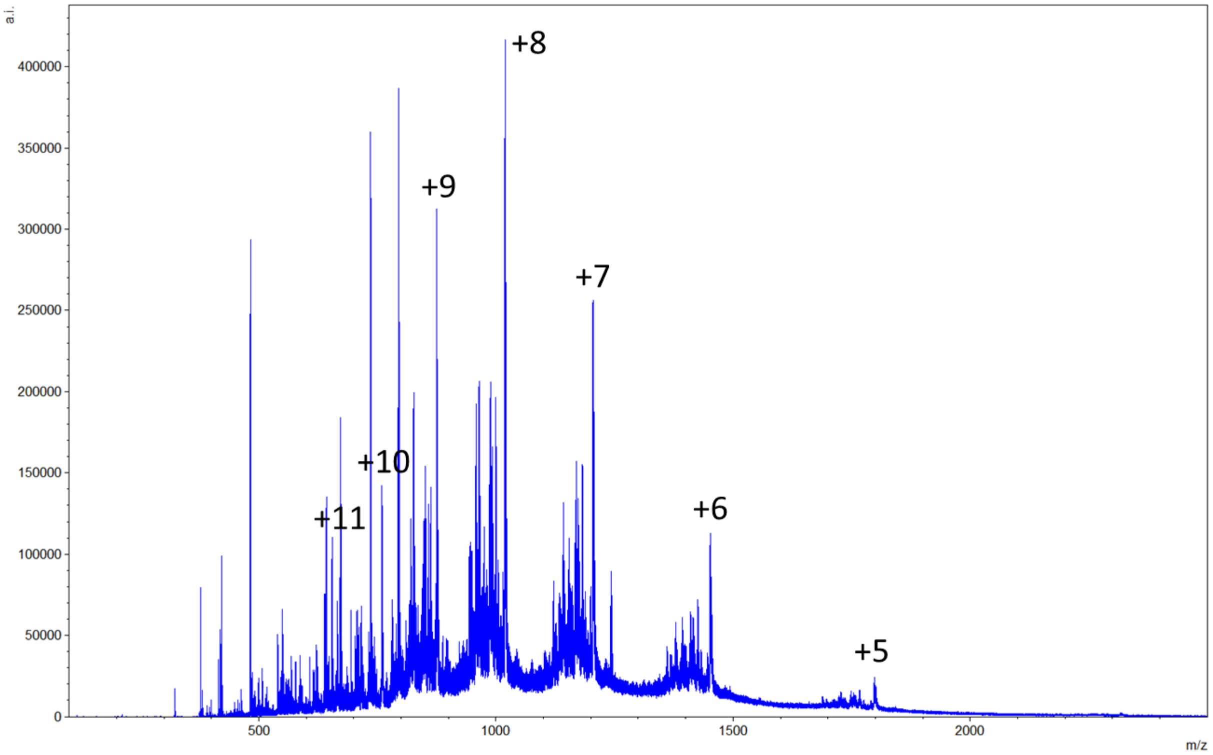

*Figure S41: High resolution ESI-MS spectrum of **2**·(NTf$_2$)$_{16}$.*

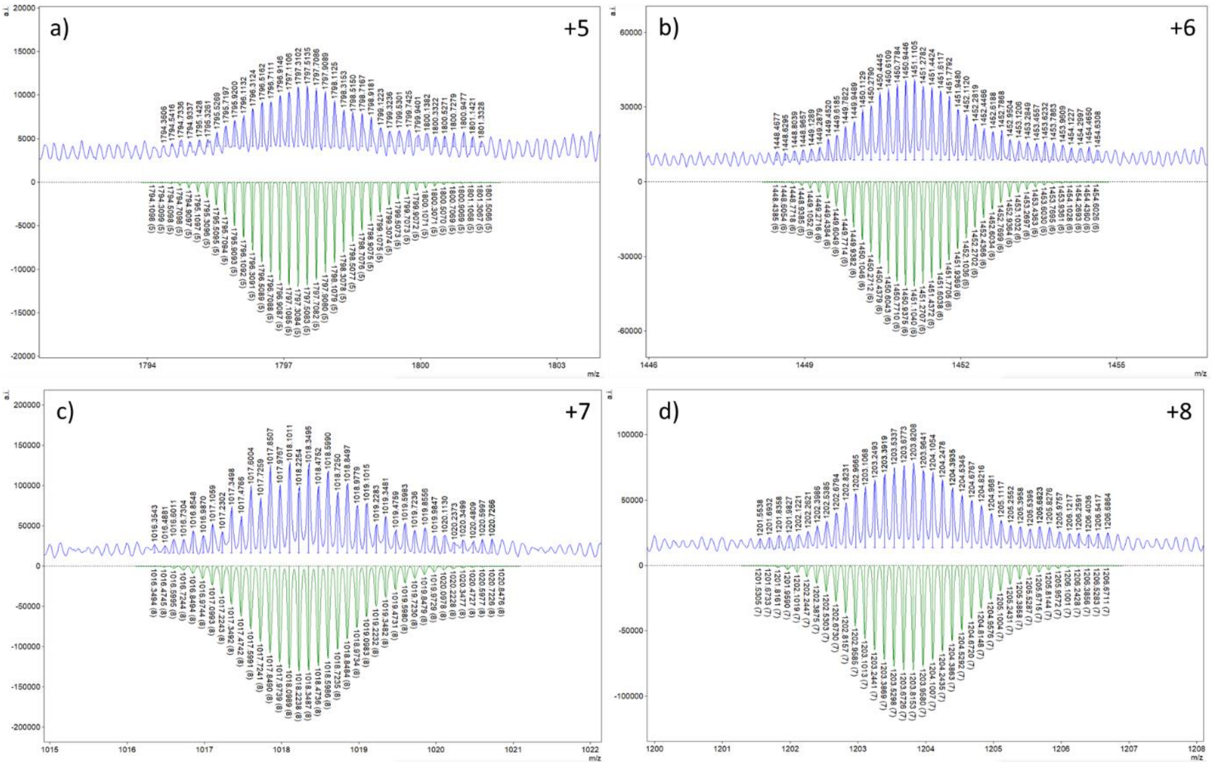

*Figure S42: Zoom into high resolution ESI-MS signals of **2**·(NTf$_2$)$_{16}$. Experimental (blue) and calculated (green) signals for a) [**2**·(NTf$_2$)$_{11}$]$^{5+}$ b) [**2**·(NTf$_2$)$_{10}$]$^{6+}$, c) [**2**·(NTf$_2$)$_9$]$^{7+}$, and d) [**2**·(NTf$_2$)$_8$]$^{8+}$.*

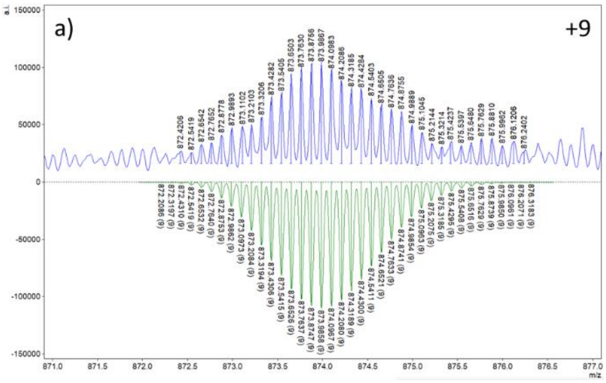

*Figure S43: Zoom into high resolution ESI-MS signals of **2**·(NTf$_2$)$_{16}$. Experimental (blue) and calculated (green) signals for a) [**2**·(NTf$_2$)$_7$]$^{9+}$.*

<u>**3**·(NTf$_2$)$_{16}$</u>

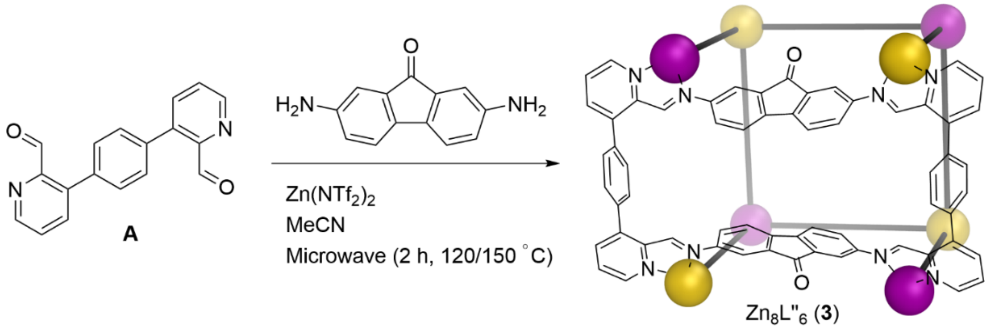

Subcomponent **A** (20.0 mg, 69.4 µmol, 1.5 eq), 2,7-diamino-9-fluorenone (14.6 mg, 69.4 µmol, 1.5 eq), and Zn(NTf$_2$)$_2$ (31.8 mg, 50.9 µmol, 1.1 eq) were added to 6 mL freshly distilled MeCN in a microwave

reactor and heated for 2 h at 120°C. After cooling, the reaction mixture was filtered over a glass fibre plug, concentrated to 1 mL and precipitated with 50 mL Et_2O. The red precipitate was collected via centrifugation, washed two times with 50 mL Et_2O, and dried in vacuo. **3**·$(NTf_2)_{16}$ was obtained as a red solid (m = 46.5 mg, 76%).

^{1}H-NMR (500 MHz, CD_3CN, 298 K): δ = 8.45 (s, 1H, a); 8.41 (d, ^3J = 4.6 Hz, 1H, b); 8.24 (d, ^3J = 7.2 Hz, 1H, c); 8.12 (s, 1H, d); 8.02 (s, 1H, e); 7.87 (dd, ^3J = 4.9 Hz, ^3J = 8.0 Hz, 1H, f); 7.27 (broad d, 1H, g); 6.68 (broad d, 1H, h); 5.39 (broad s, 1H, i). Please note that the broadness of the peaks did not allow determination of the coupling constants for g and h.**^{13}C-NMR** (126 MHz, CD_3CN, 298 K): δ = 190.8, 165.4, 148.8, 144.7, 144.4, 143.7, 136.5, 135.9, 134.5, 131.0, 130.7, 129.9, 127.4, 126.3, 119.2, 120.8 (q, *J* = 321.4 Hz, NTf_2). **^{19}F-NMR** (376 MHz, CD_3CN, 298 K): δ = -80.4. **ESI-HRMS**: ([**3**·$(NTf_2)_{16}$] = (C62H36N8O2)6Zn8(C2F6NO4S2)16 m/z = 679.4 [**3**·$(NTf_2)_5$]$^{11+}$ (calc. 679.4), 775.4 [**3**·$(NTf_2)_6$]$^{10+}$ (calc. 775.4), 892.6 [**3**·$(NTf_2)_7$]$^{9+}$ (calc. 892.7), 1039.2 [**3**·$(NTf_2)_8$]$^{8+}$ (calc. 1039.3), 1227.6 [**3**·$(NTf_2)_{19}$]$^{7+}$ (calc. 1227.8), 1479.1 [**3**·$(NTf_2)_{10}$]$^{6+}$ (calc. 1479.1), 1830.9 [**3**·$(NTf_2)_{11}$]$^{5+}$ (calc. 1831.0), 2358.5 [**3**·$(NTf_2)_{12}$]$^{4+}$ (calc. 2358.7).

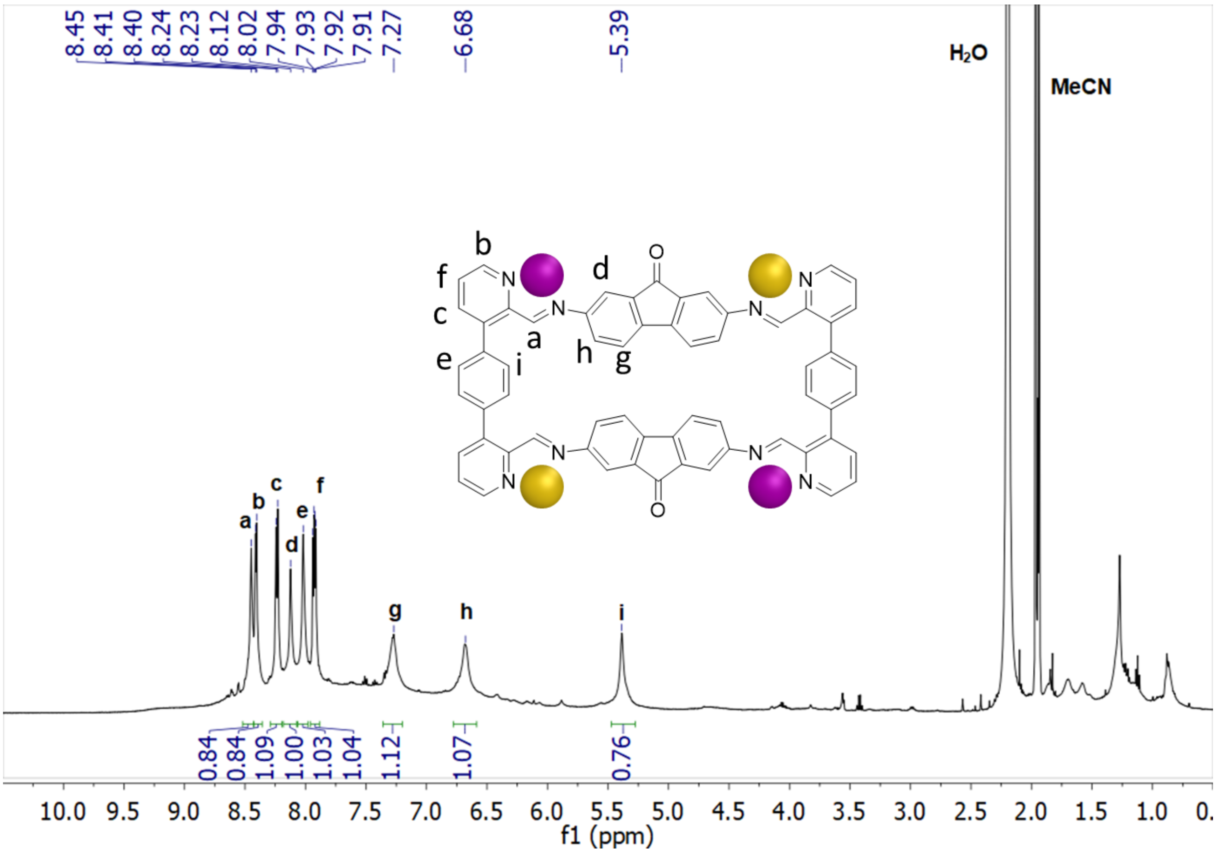

*Figure S44: ^{1}H NMR spectrum of **3**·$(NTf_2)_{16}$ (500 MHz, CD_3CN, 298 K).*

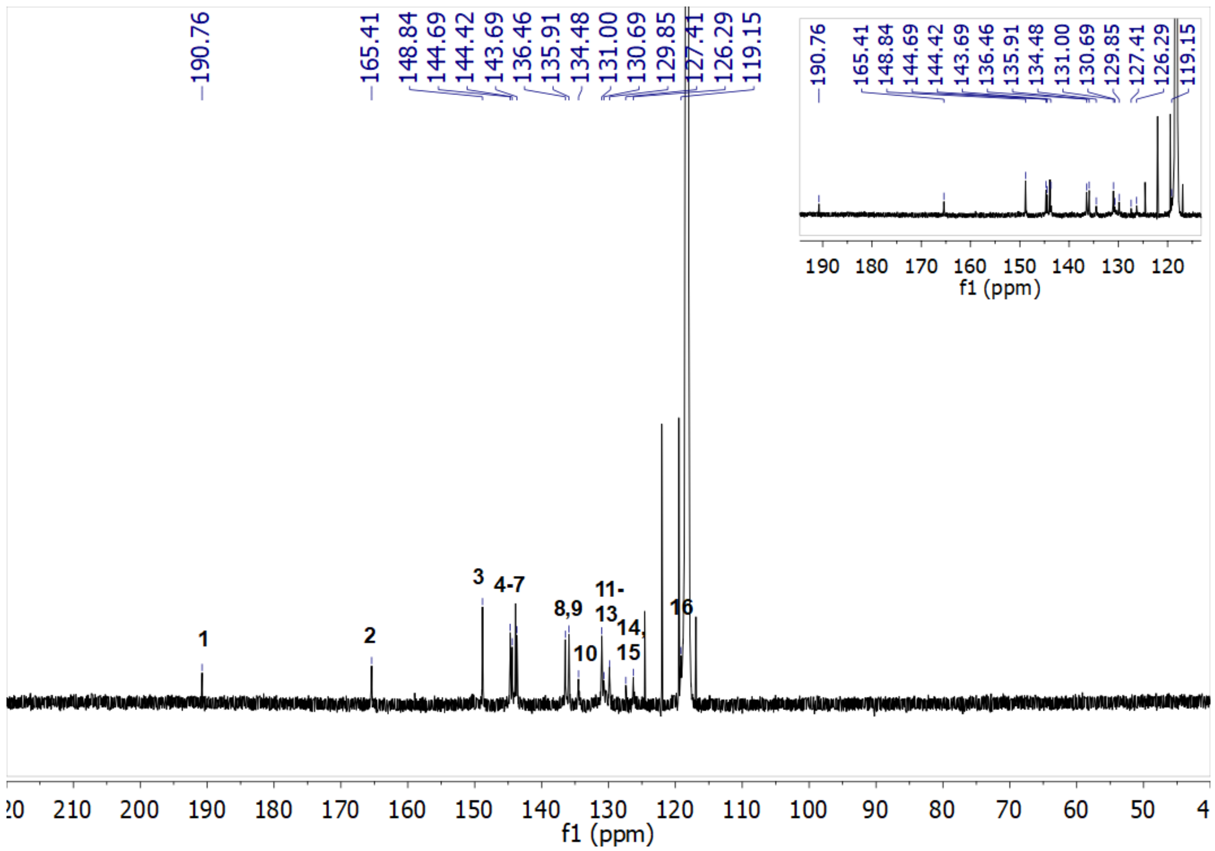

*Figure S45: ^{13}C NMR spectrum of **3**·(NTf$_2$)$_{16}$ (126 MHz, CD$_3$CN, 298 K).*

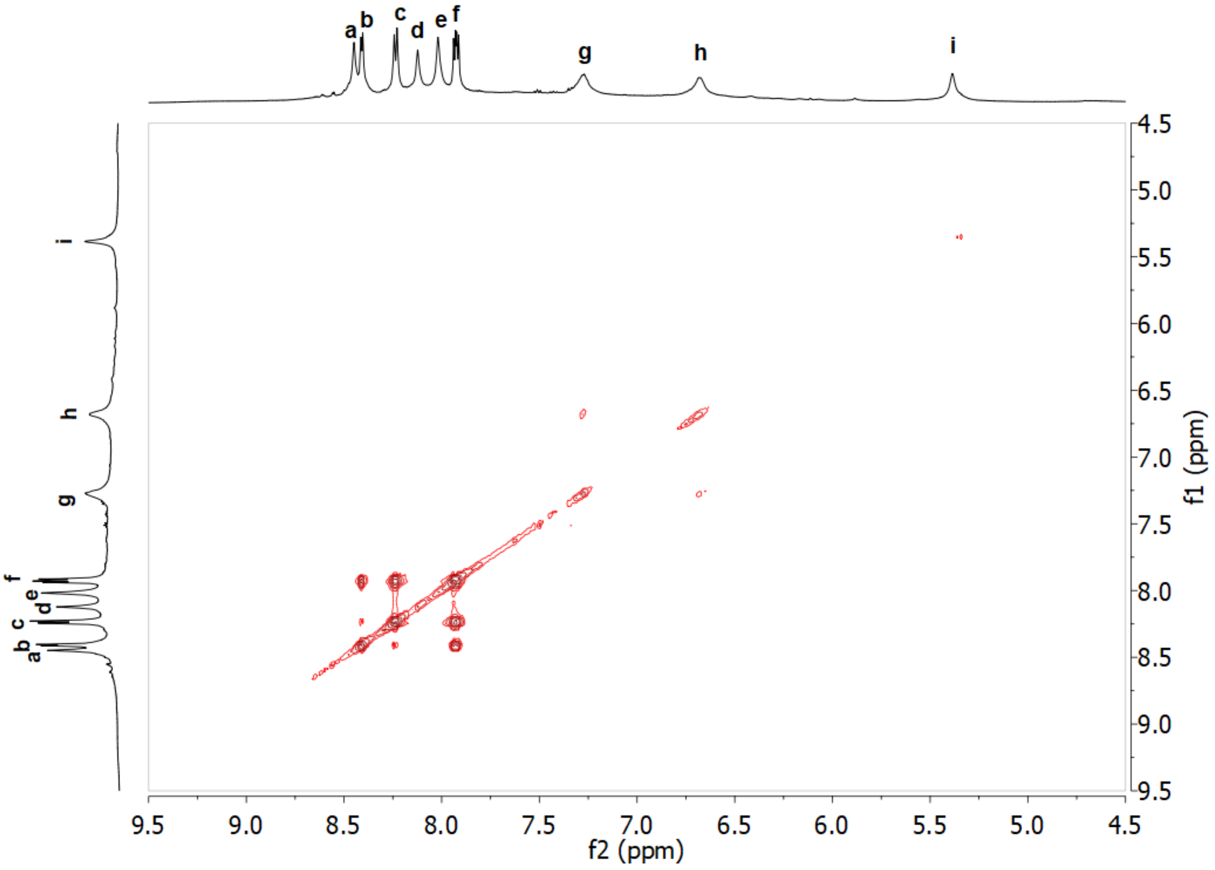

*Figure S46: ^{1}H-^{1}H COSY spectrum of **3**·(NTf$_2$)$_{16}$ (500 MHz, CD$_3$CN, 298 K).*

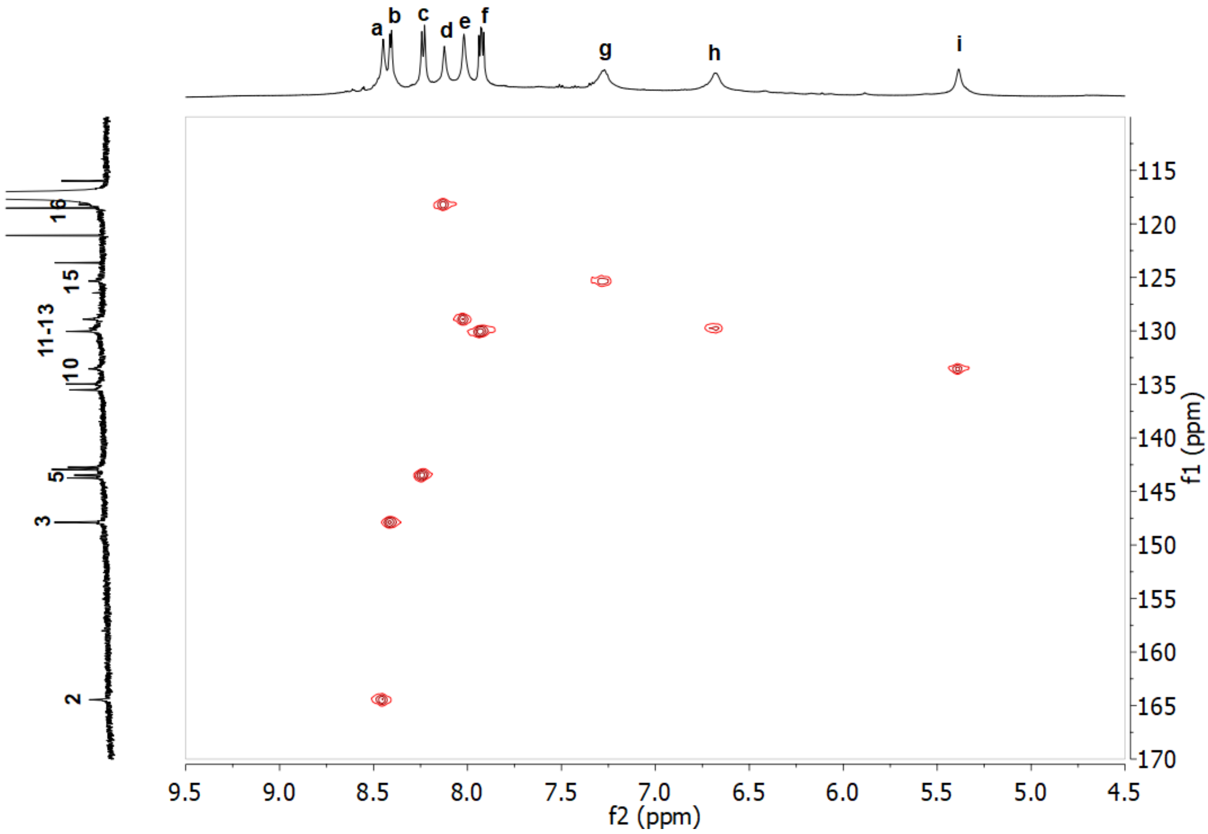

*Figure S47: ^{1}H-^{13}C HSQC spectrum of **3**·$(NTf_2)_{16}$ (500 MHz, 126 MHz, CD_3CN, 298 K).*

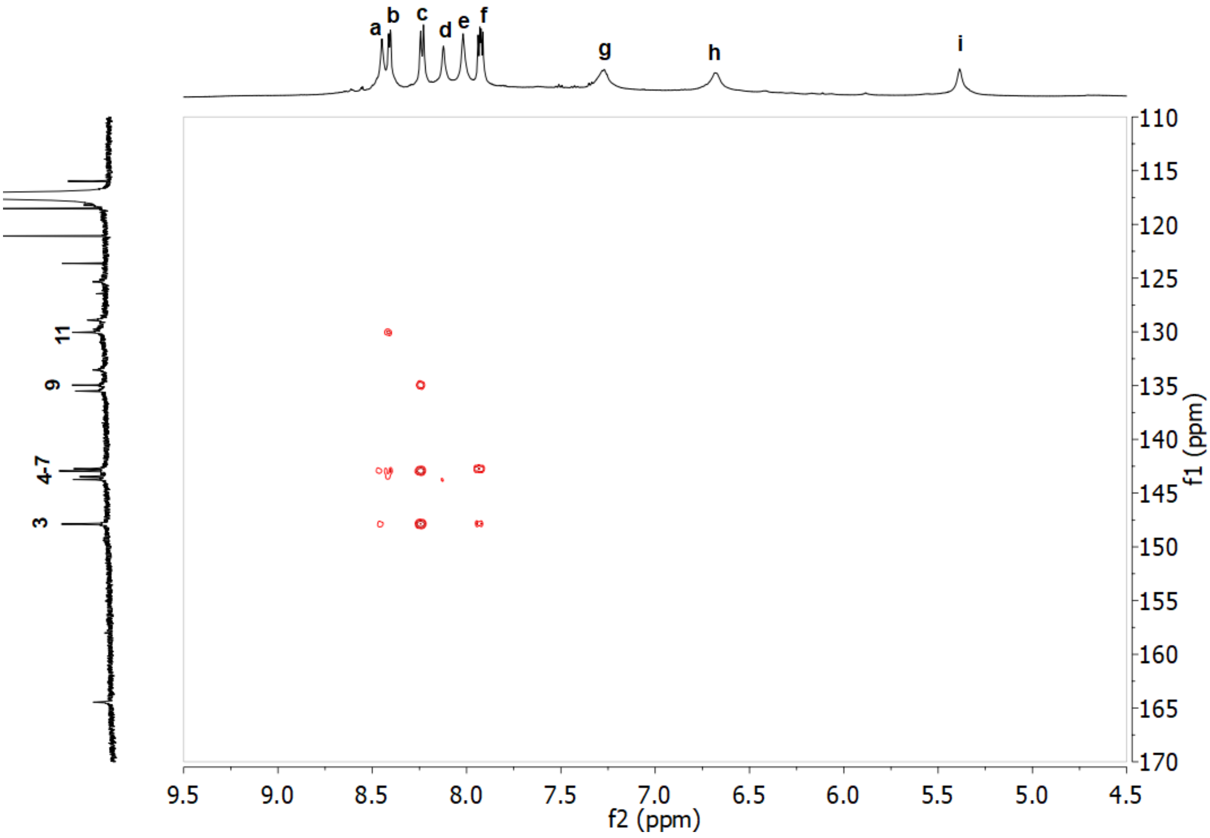

*Figure S48: ^{1}H-^{13}C HMBC spectrum of **3**·$(NTf_2)_{16}$ (500 MHz, 126 MHz, CD_3CN, 298 K).*

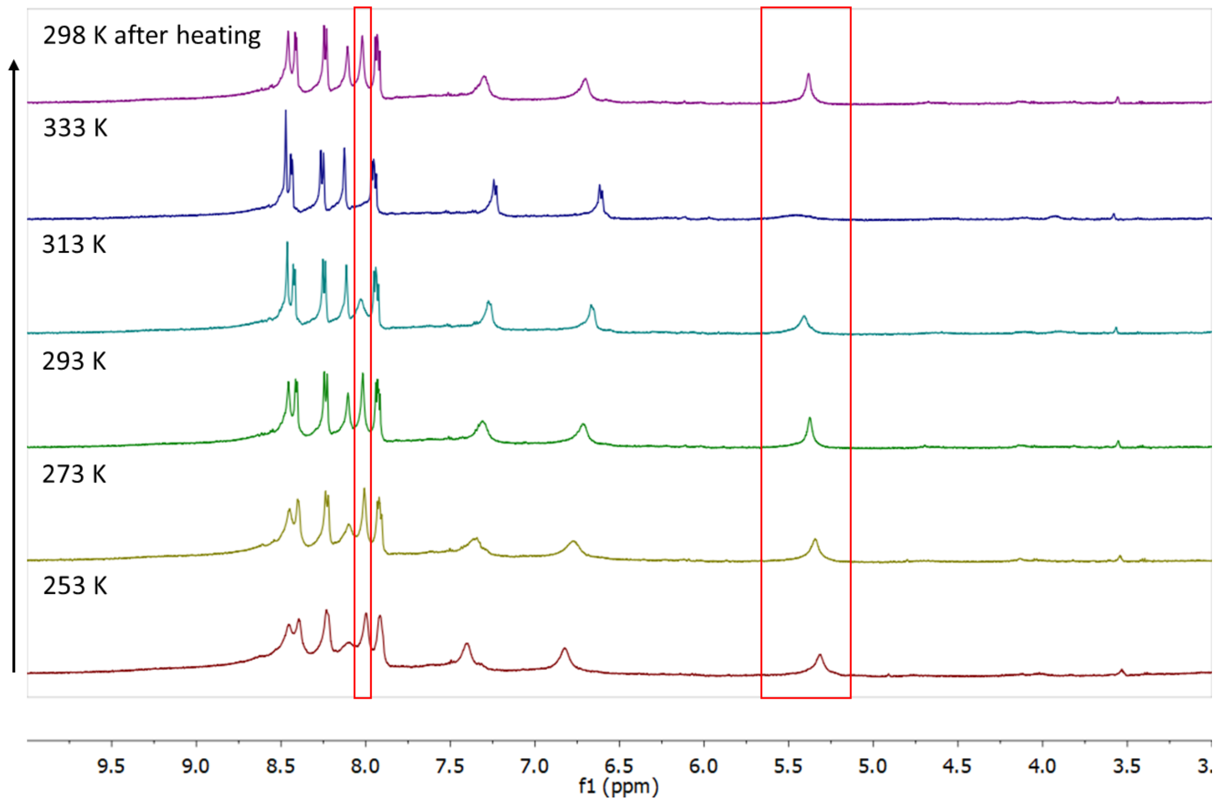

*Figure S49: Variable-temperature ^{1}H NMR spectra of **3**·$(NTf_2)_{16}$ (500 MHz, CD_3CN). Upon temperature increase, the increased rotational freedom results in significant broadening of the signals of protons e and i and a shift towards each other. However, even at 333 K coalescence was not observed. A control measurement at 298 K after heating showed the reappearance of protons e and i.*

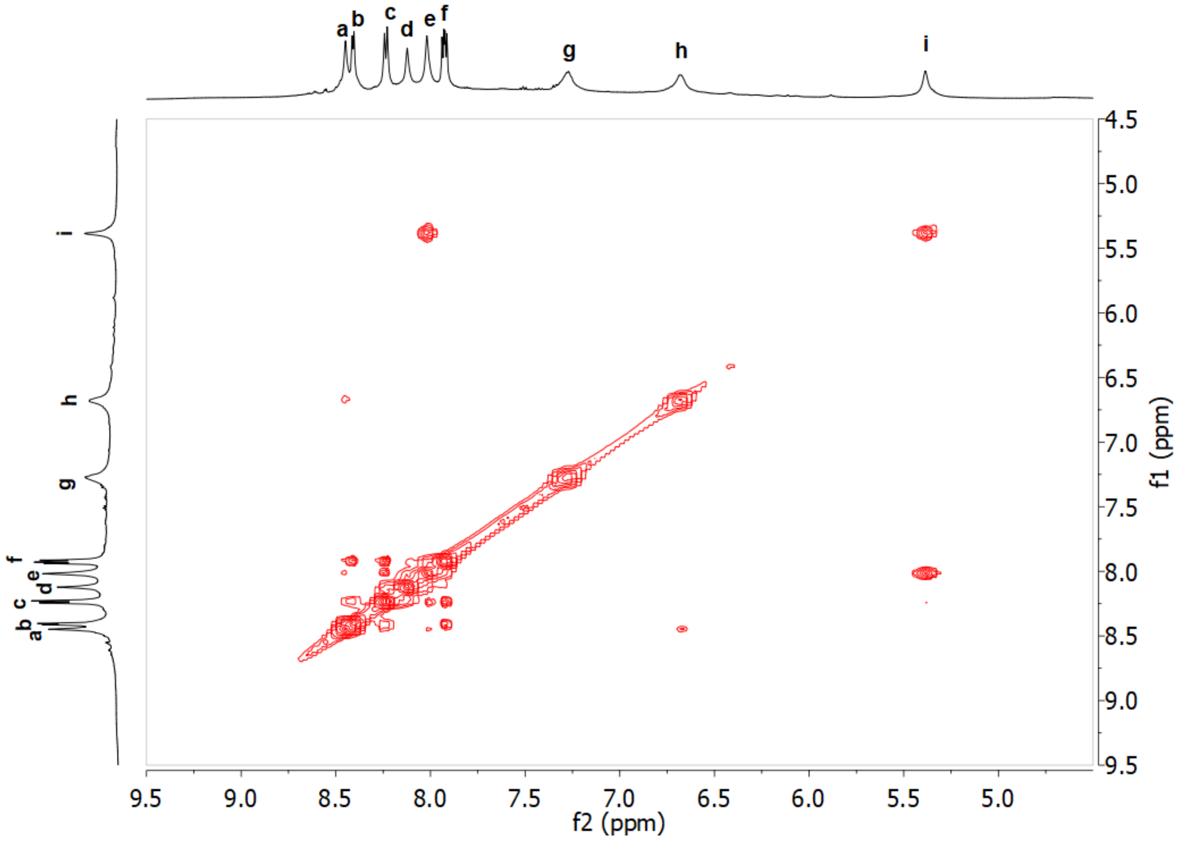

*Figure S50: ^{1}H-^{1}H NOESY spectrum of **3**·$(NTf_2)_{16}$ (500 MHz, CD_3CN, 298 K).*

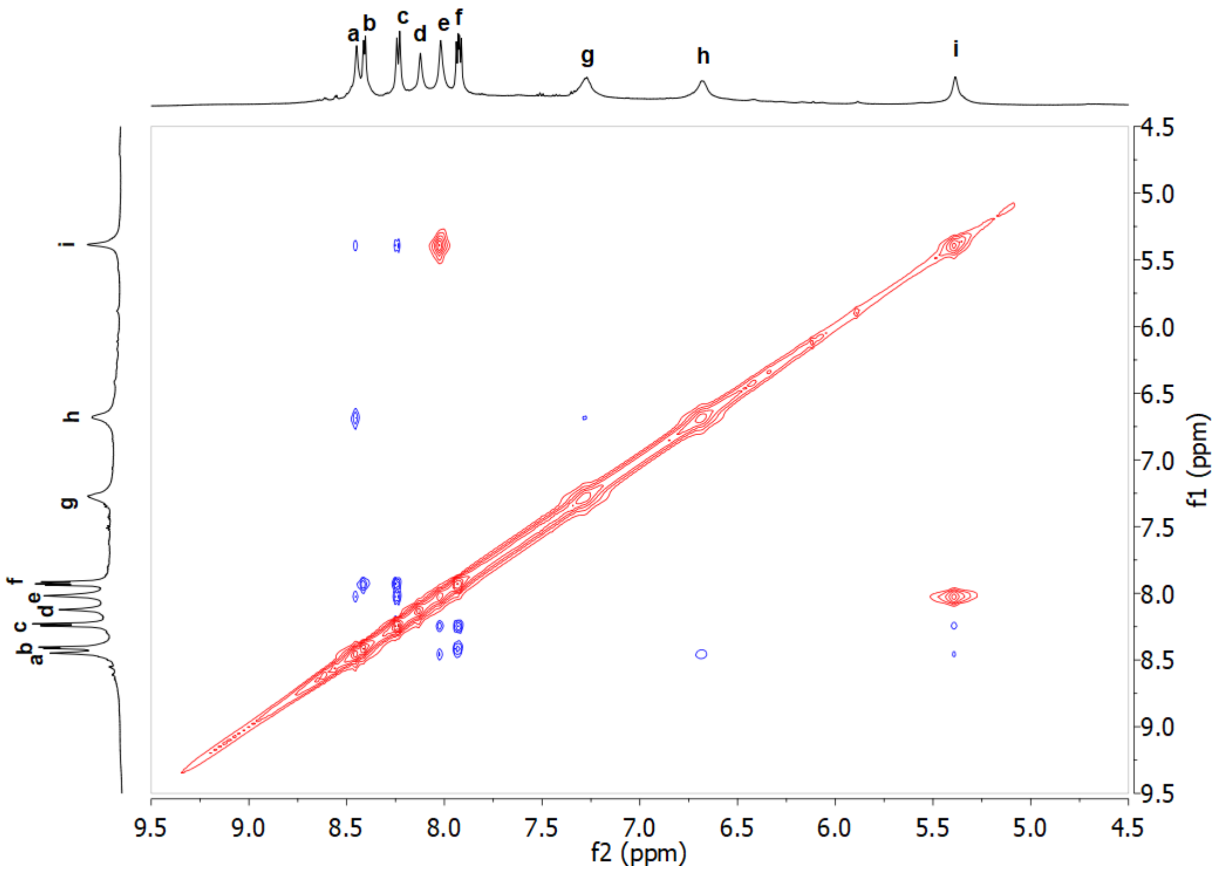

*Figure S51: 1H-1H ROESY spectrum of **3**·$(NTf_2)_{16}$ (500 MHz, CD_3CN, 298 K).*

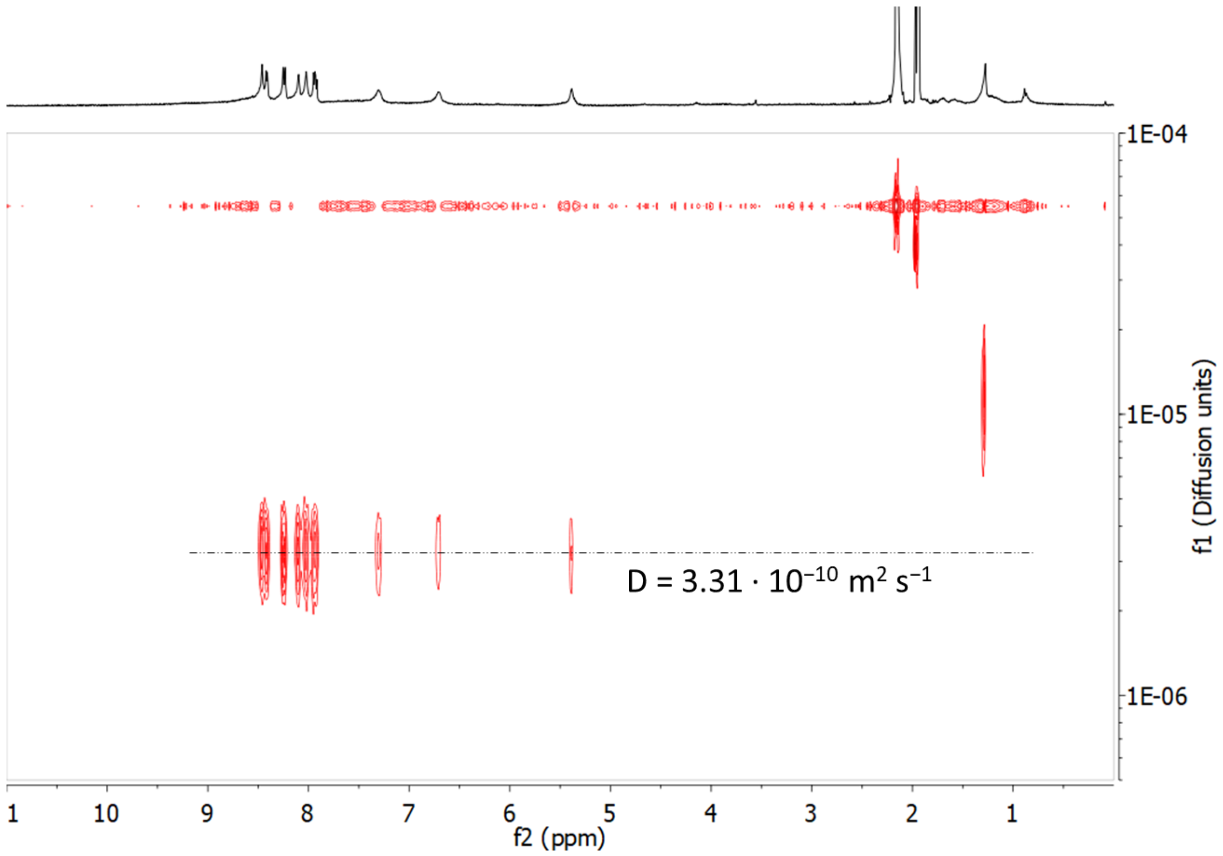

*Figure S52: ^{1}H DOSY spectrum of **3**·$(NTf_2)_{16}$ (400 MHz, CD_3CN, 298 K).*

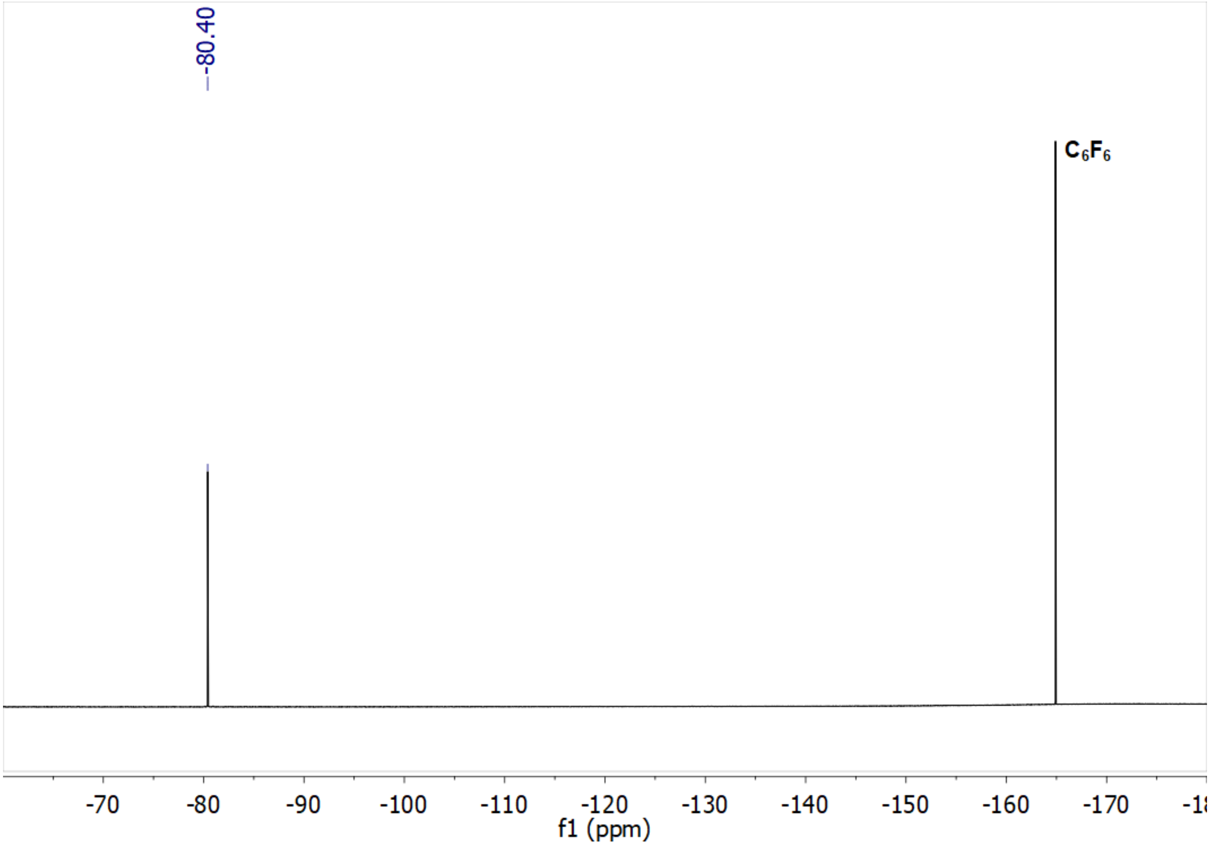

*Figure S53: ^{19}F NMR spectrum of **3**·(NTf$_2$)$_{16}$ (376 MHz, CD$_3$CN, 298 K).*

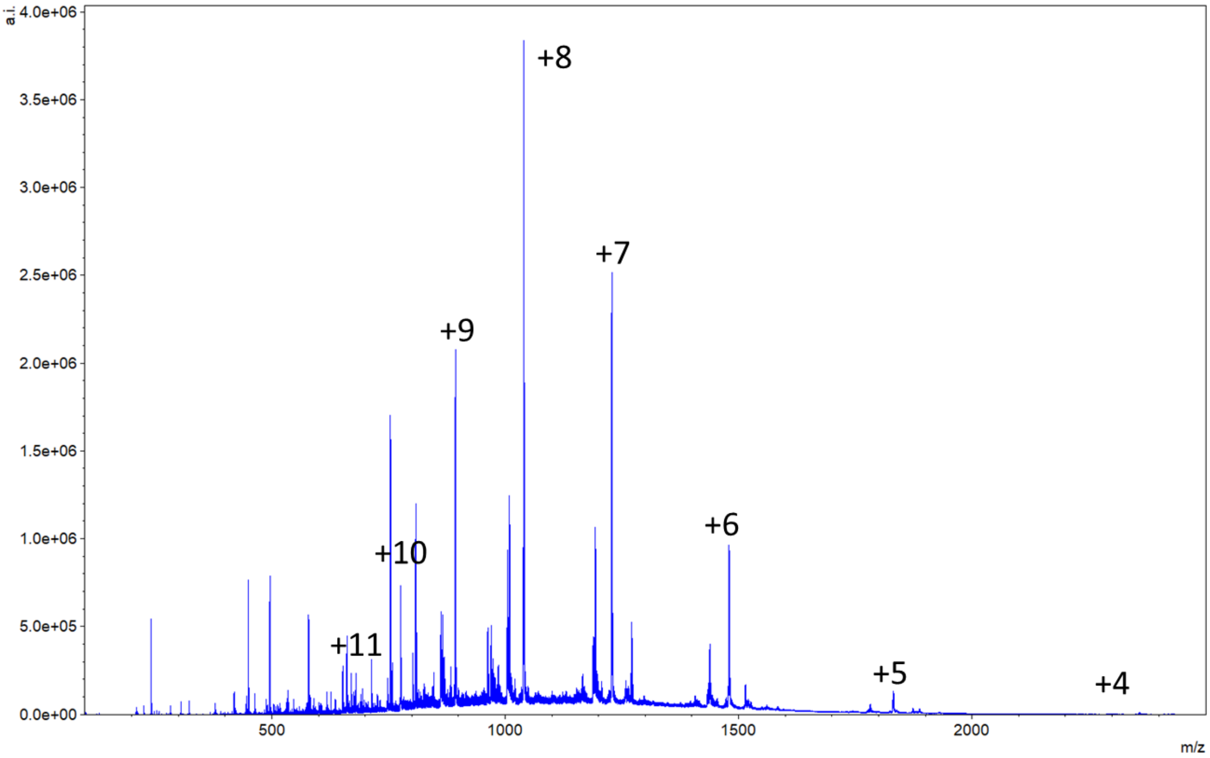

*Figure S54: High resolution ESI-MS spectrum of **3**·(NTf$_2$)$_{16}$.*

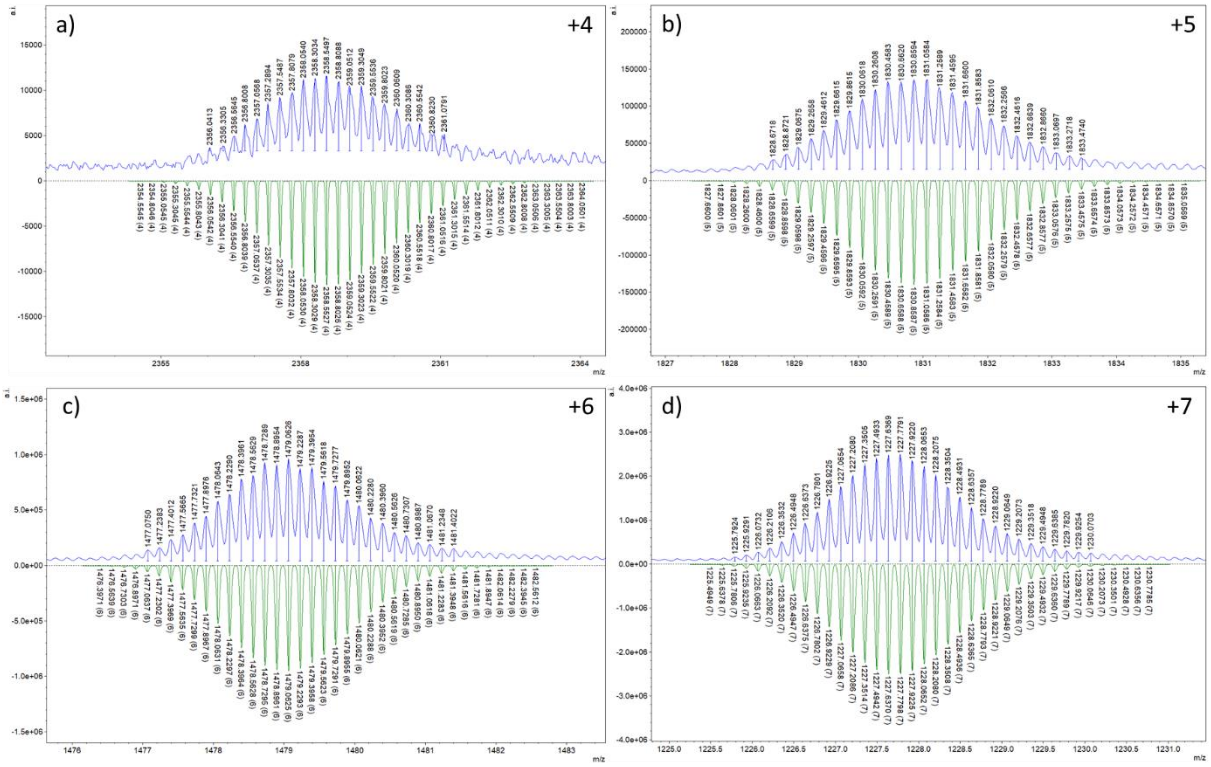

*Figure S55: Signals from the high-resolution ESI-mass spectrum for **3**·$(NTf_2)_{16}$. Experimental (blue) and calculated (green) signals for a) [**3**$(NTf_2)_{11}]^{4+}$ b) [**3**$(NTf_2)_{10}]^{5+}$ c) [**3**$(NTf_2)_9]^{6+}$, and d) [**3**$(NTf_2)_8]^{7+}$.*

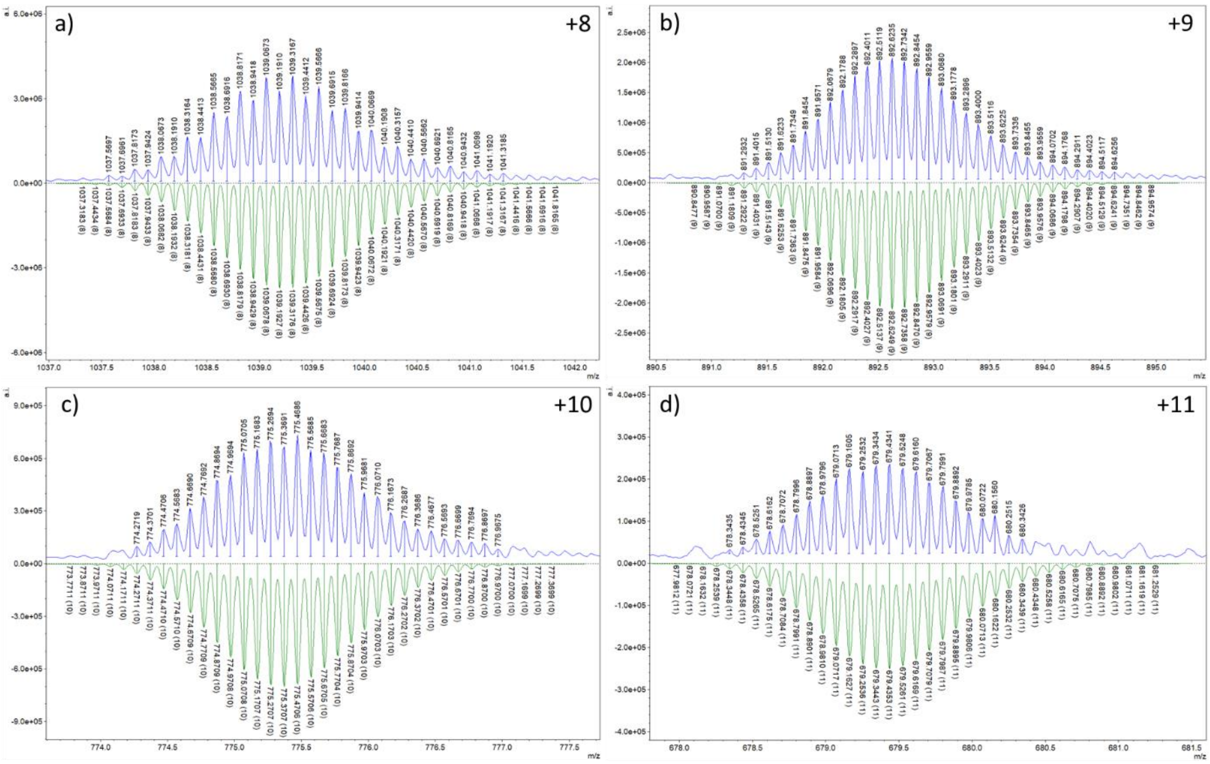

*Figure S56: Signals from the high-resolution ESI-mass spectrum for **3**·$(NTf_2)_{16}$. Experimental (blue) and calculated (green) signals for a) [**3**$(NTf_2)_{11}]^{8+}$ b) [**3**$(NTf_2)_{10}]^{9+}$ c) [**3**$(NTf_2)_9]^{10+}$, and d) [**3**$(NTf_2)_8]^{11+}$.*

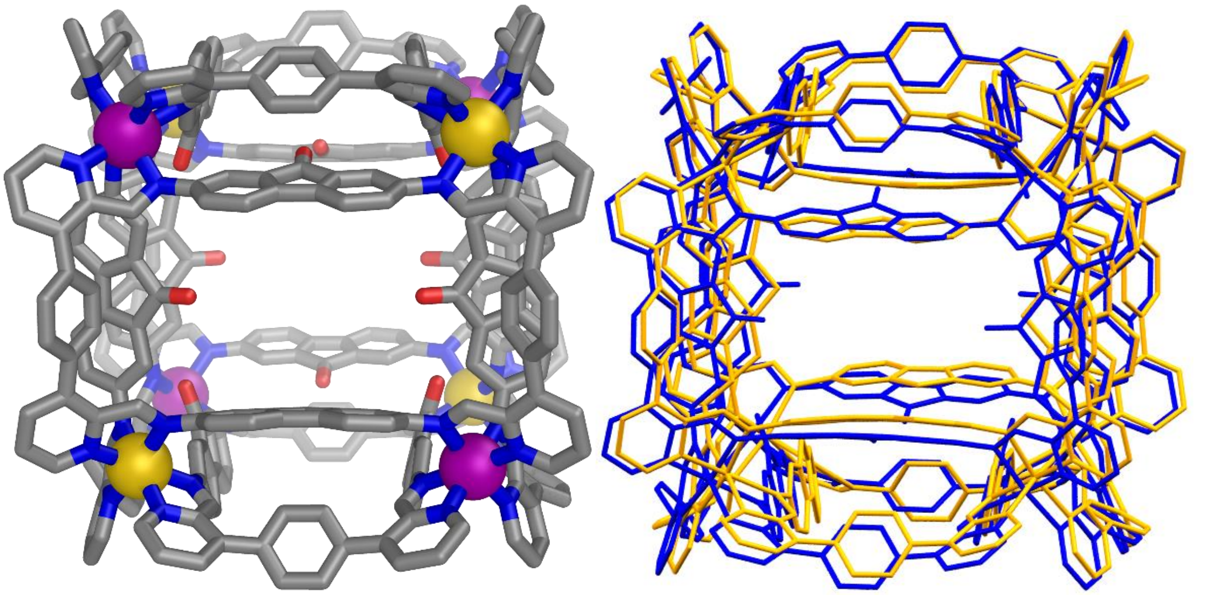

*Figure S57: GFN2-xTB-optimized structure of **3**; protons are not shown (left). Calculations were performed without inclusion of counteranions or solvent molecules. Comparison of the crystal structure of **2** (orange) and the GFN2-xTB-optimized structure of **3** (blue), highlighting the similarity between them.*

4·$(NTf_2)_{16}$

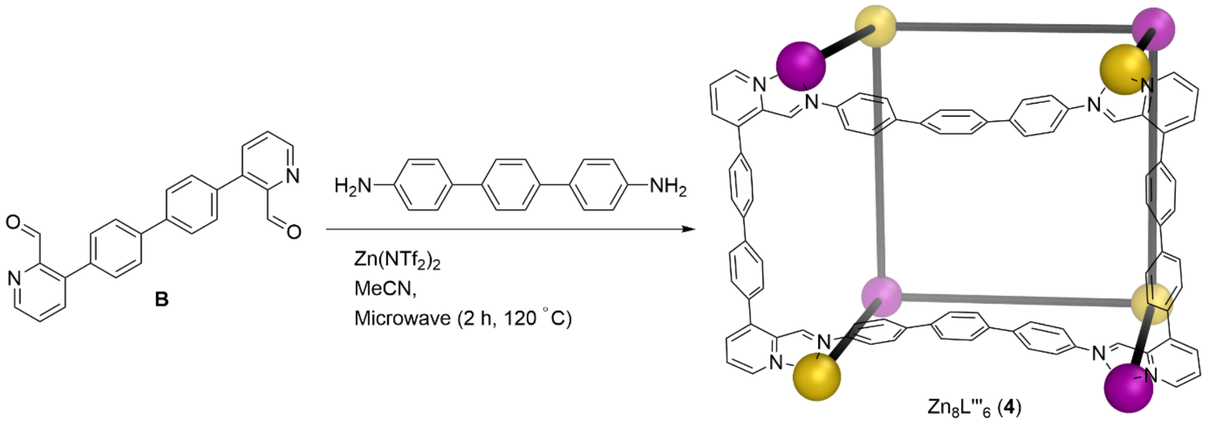

Subcomponent **B** (20.0 mg, 54.9 µmol, 1.5 eq), 4,4''-diamino-*p*-terphenyl (14.3 mg, 54.9 µmol, 1.5 eq), and $Zn(NTf_2)_2$ (25.2 mg, 40.2 µmol, 1.1 eq) were added to 5 mL freshly distilled MeCN in a microwave reactor and heated for 2 h at 120°C. After cooling, the reaction mixture was filtered over a glass fibre plug, concentrated to 1 mL and precipitated with 50 mL Et_2O. The red precipitate was collected via centrifugation, washed two times with 50 mL Et_2O, and dried in vacuo. **3**·$(NTf_2)_{16}$ was obtained as a red solid (31.7 mg, 57%).

^{1}H-NMR (500 MHz, CD_3CN, 298 K): δ = 8.62 (d, ^3J = 4.7 Hz, 1H, a); 8.35 (s, 1H, b); 8.28 (d, ^3J = 7.8 Hz, 1H, c); 7.94 (dd, ^3J = 4.9 Hz, ^3J = 7.9 Hz, 1H, d); 7.78 (d, ^3J = 8.1 Hz, 2H, e); 7.61 (3, 2H, f); 7.48 (d, ^3J = 8.3 Hz, 2H, g); 7.35 (broad d, 2H, h) ; 6.68 (d, ^3J = 7.7 Hz, 2H, i). **^{13}C-NMR** (126 MHz, CD_3CN, 298 K): δ = 163.3, 148.9, 146.9, 144.7, 144.1, 142.9, 141.2, 139.1, 138.2, 134.4, 131.5, 130.3, 129.1, 128.5, 127.2, 123.6, 120.9 (q, *J* = 325.1 Hz, NTf_2). **^{19}F-NMR** (376 MHz, CD_3CN, 298 K): δ = -80.4. **ESI-HRMS**: ([**4**·$(NTf_2)_{16}$] = (C84H56N8)6Zn8(C2F6NO4S2)16 m/z = 581.9 [**4**·$(NTf_2)_2]^{14+}$ (calc. 582.0), 948.3 [**4**·$(NTf_2)_3]^{13+}$ (calc. 648.3), 725.6 [**4**·$(NTf_2)_4]^{12+}$ (calc. 725.7), 817.1 [**4**·$(NTf_2)_5]^{11+}$ (calc. 817.1), 926.8 [**4**·$(NTf_2)_6]^{10+}$ (calc. 926.8), 1060.8 [**4**·$(NTf_2)_7]^{9+}$ (calc. 1061.0), 1228.4 [**4**·$(NTf_2)_8]^{8+}$ (calc. 1228.6), 1444.1 [**4**·$(NTf_2)_{19}]^{7+}$ (calc. 1444.1), 1731.4 [**4**·$(NTf_2)_{10}]^{6+}$ (calc. 1731.5), 2133.6 [**4**·$(NTf_2)_{11}]^{5+}$ (calc. 2133.8).

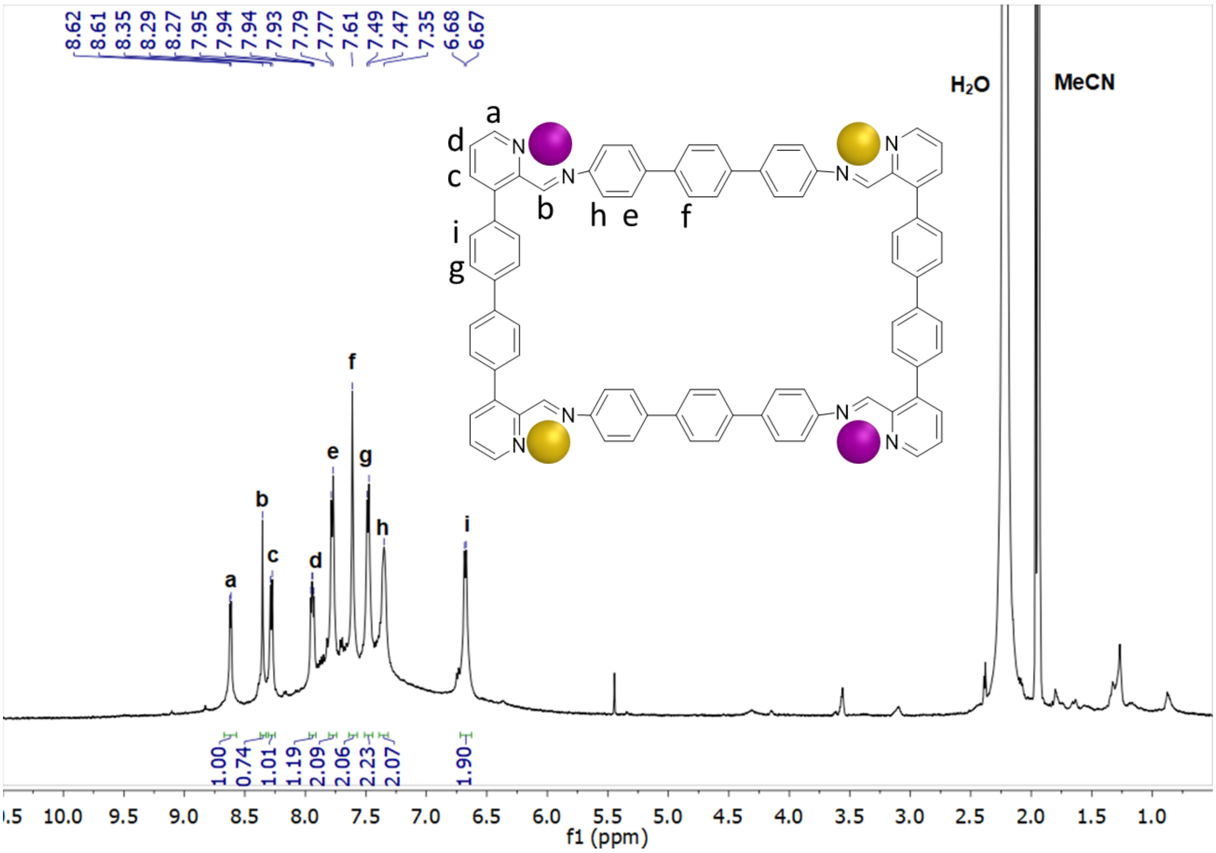

*Figure S58: ^{1}H NMR spectrum of **4**·(NTf$_2$)$_{16}$ (500 MHz, CD$_3$CN, 298 K).*

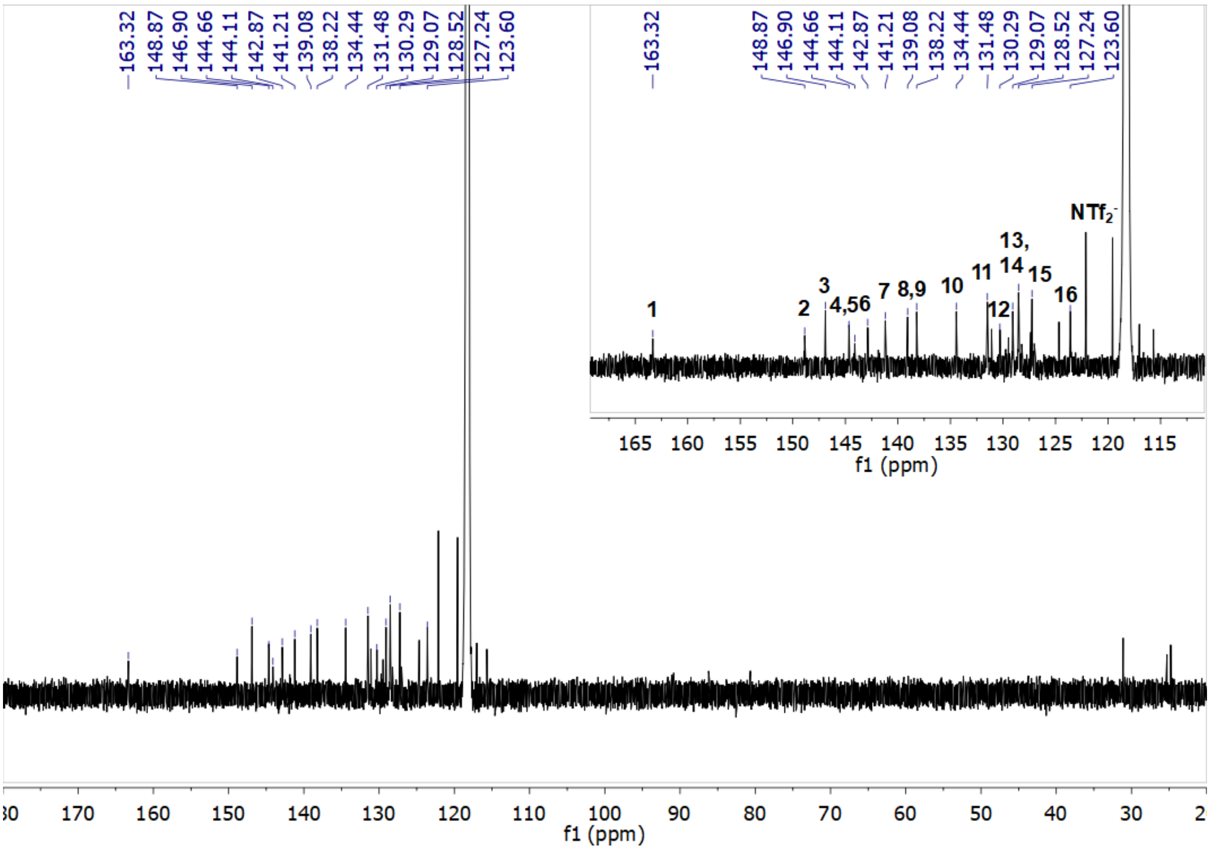

*Figure S59: ^{13}C NMR spectrum of **4**·(NTf$_2$)$_{16}$ (126 MHz, CD$_3$CN, 298 K).*

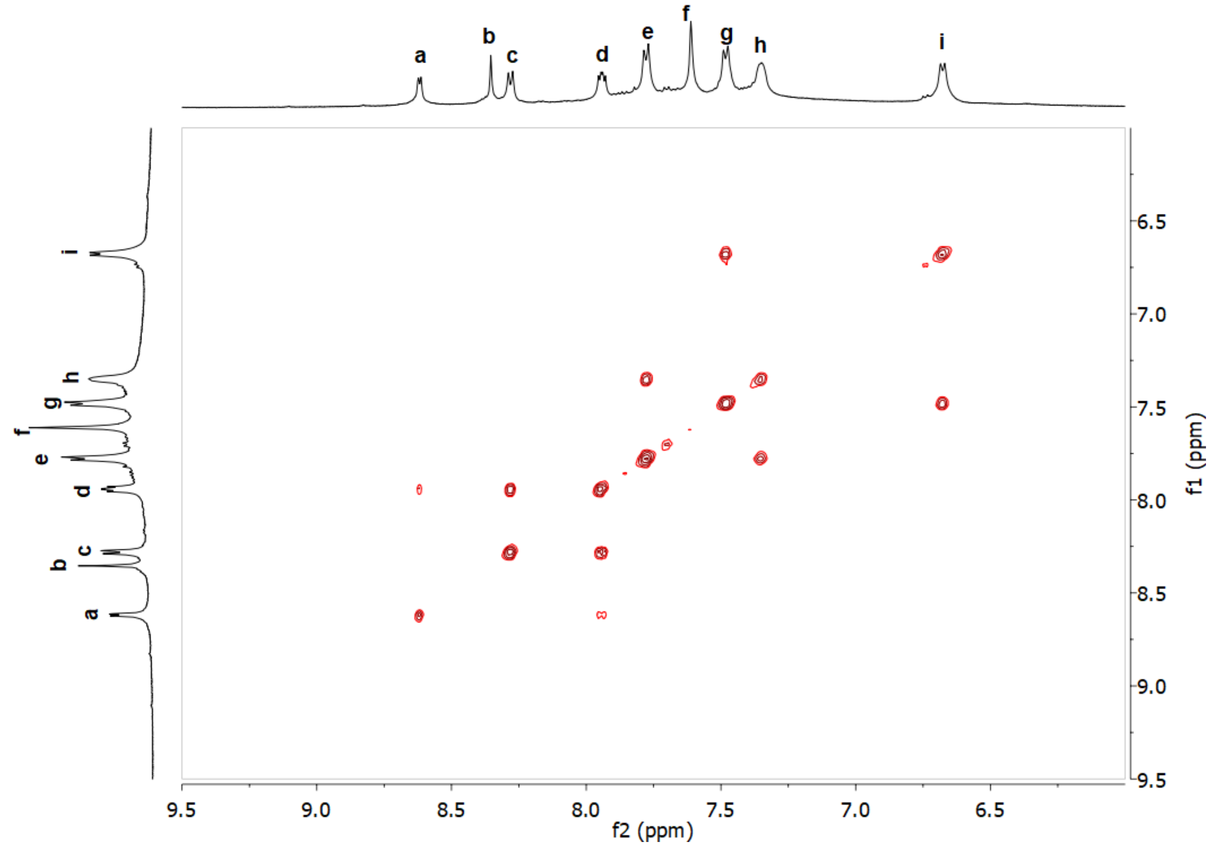

*Figure S60: ^{1}H-^{1}H COSY spectrum of **4**·$(NTf_2)_{16}$ (500 MHz, CD_3CN, 298 K).*

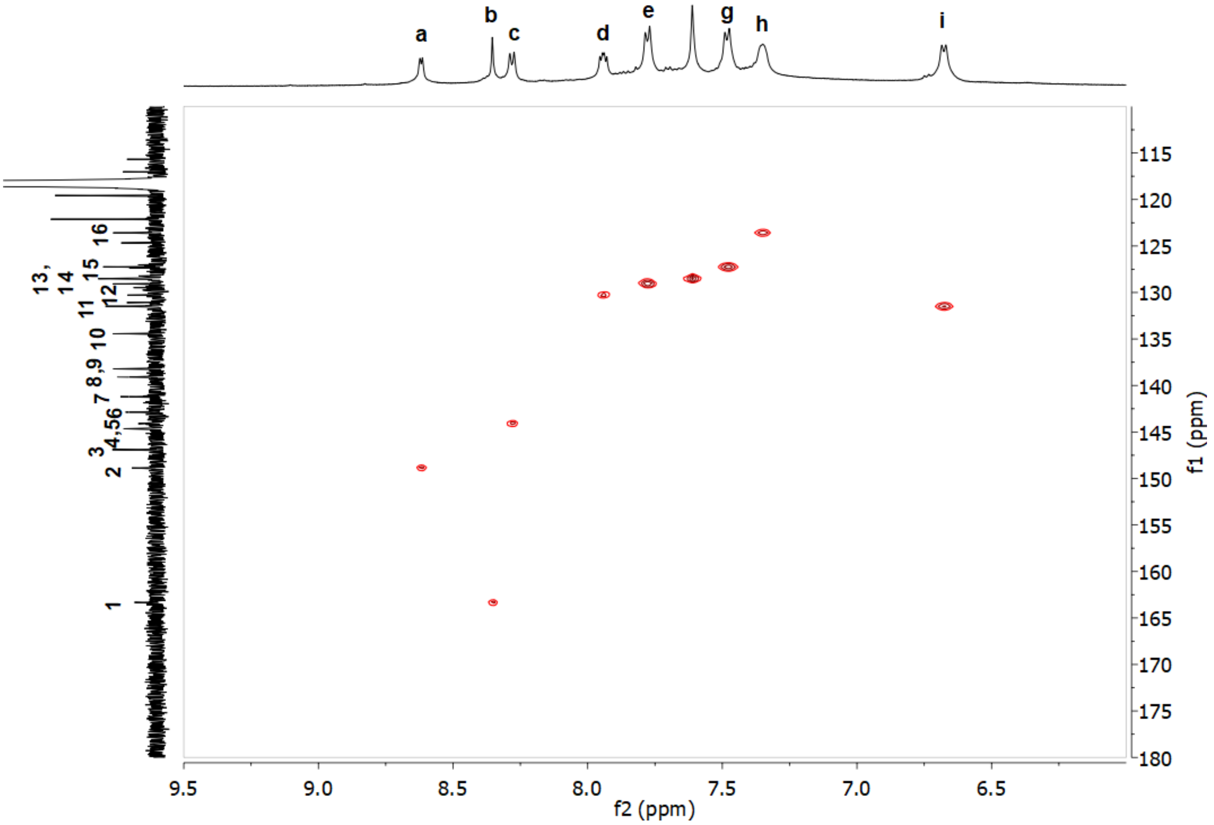

*Figure S61: ^{1}H-^{13}C HSQC spectrum of **4**·$(NTf_2)_{16}$ (500 MHz, 126 MHz, CD_3CN, 298 K).*

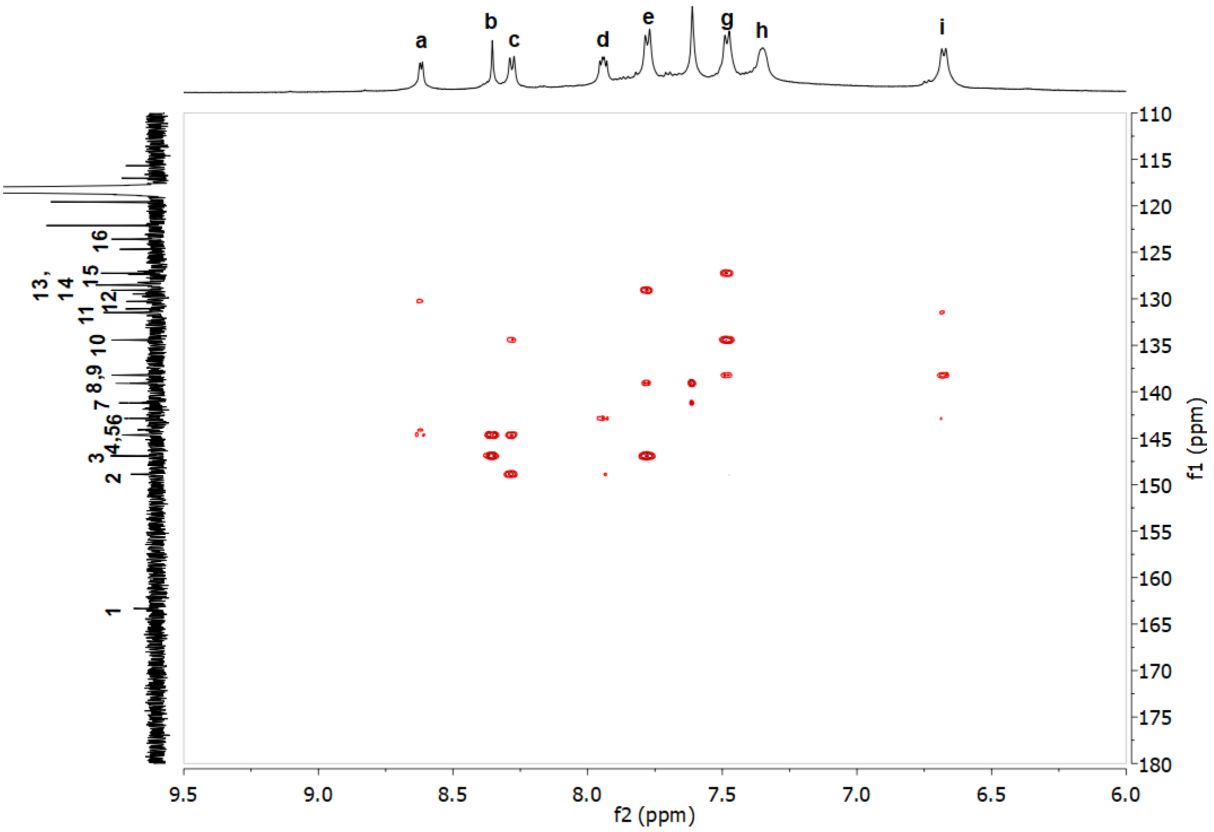

*Figure S62: ^{1}H-^{13}C HMBC spectrum of **4**·$(NTf_2)_{16}$ (500 MHz, 126 MHz, CD_3CN, 298 K).*

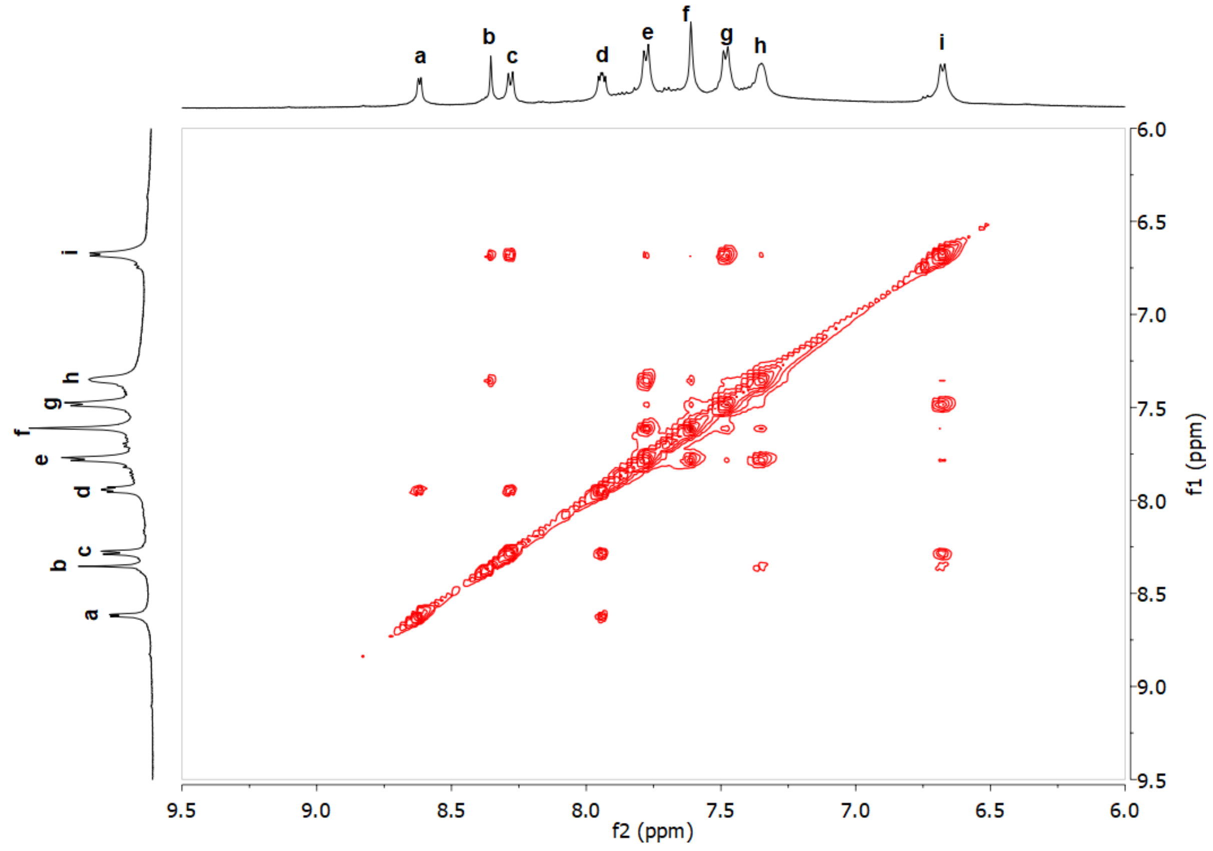

*Figure S63: ^{1}H-^{1}H NOESY spectrum of **4**·$(NTf_2)_{16}$ (500 MHz, CD_3CN, 298 K).*

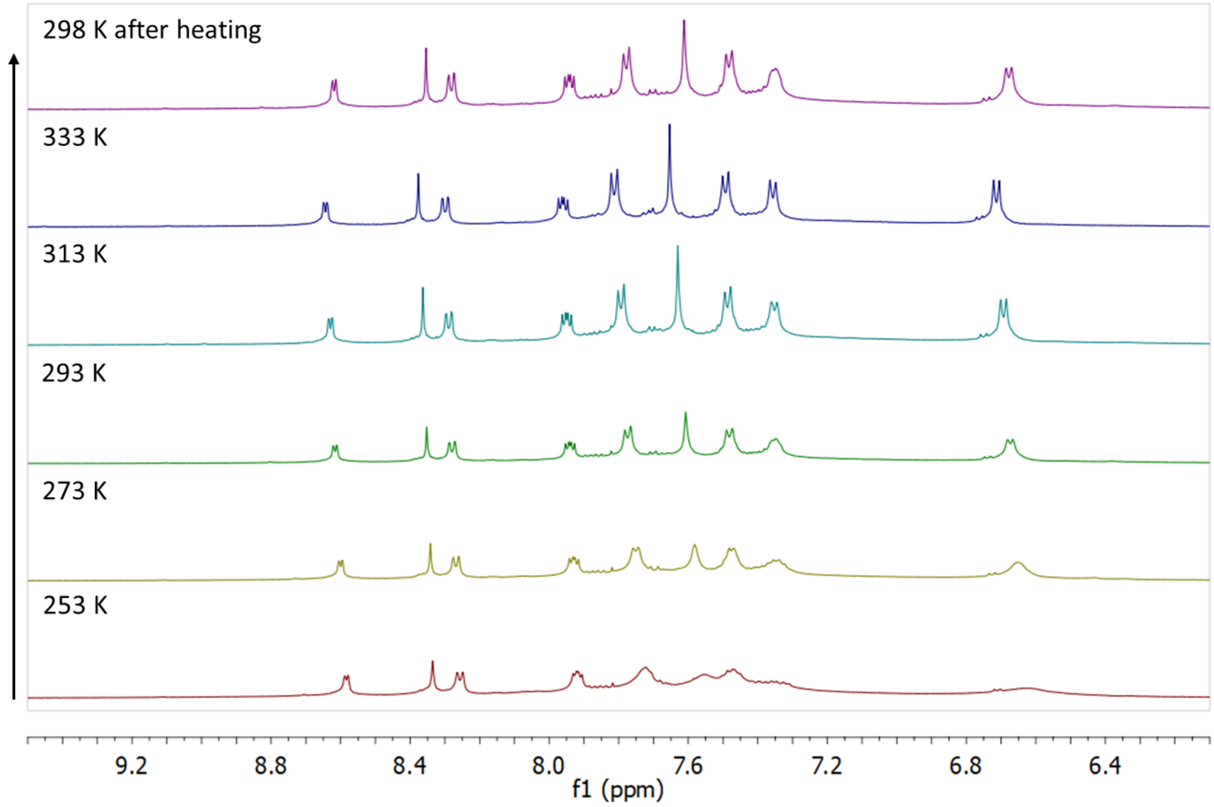

*Figure S64: Variable-temperature ^{1}H NMR spectra of **4**·$(NTf_2)_{16}$ (500 MHz, CD_3CN). A control measurement at 298 K after heating confirmed that heating did not affect the cage structure.*

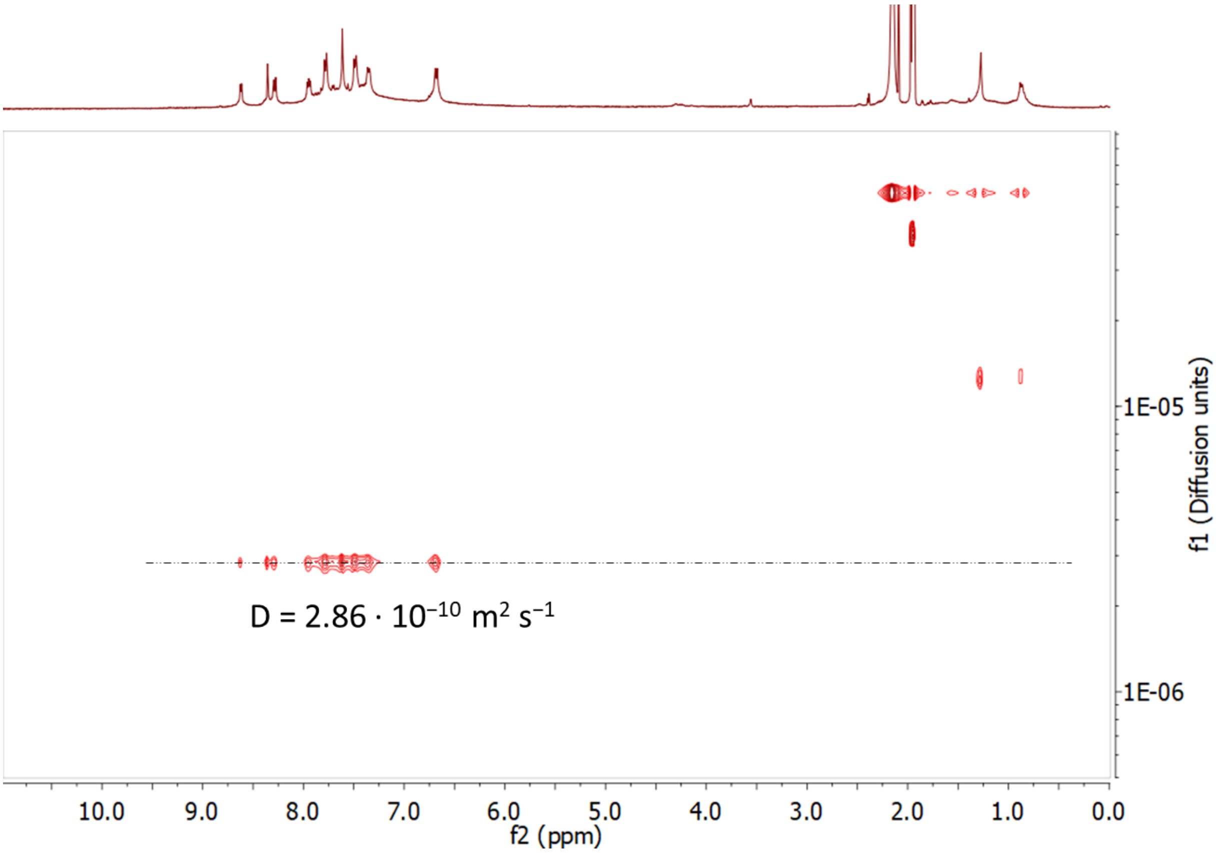

*Figure S65: ^{1}H DOSY spectrum of **4**·$(NTf_2)_{16}$ (400 MHz, CD_3CN, 298 K).*

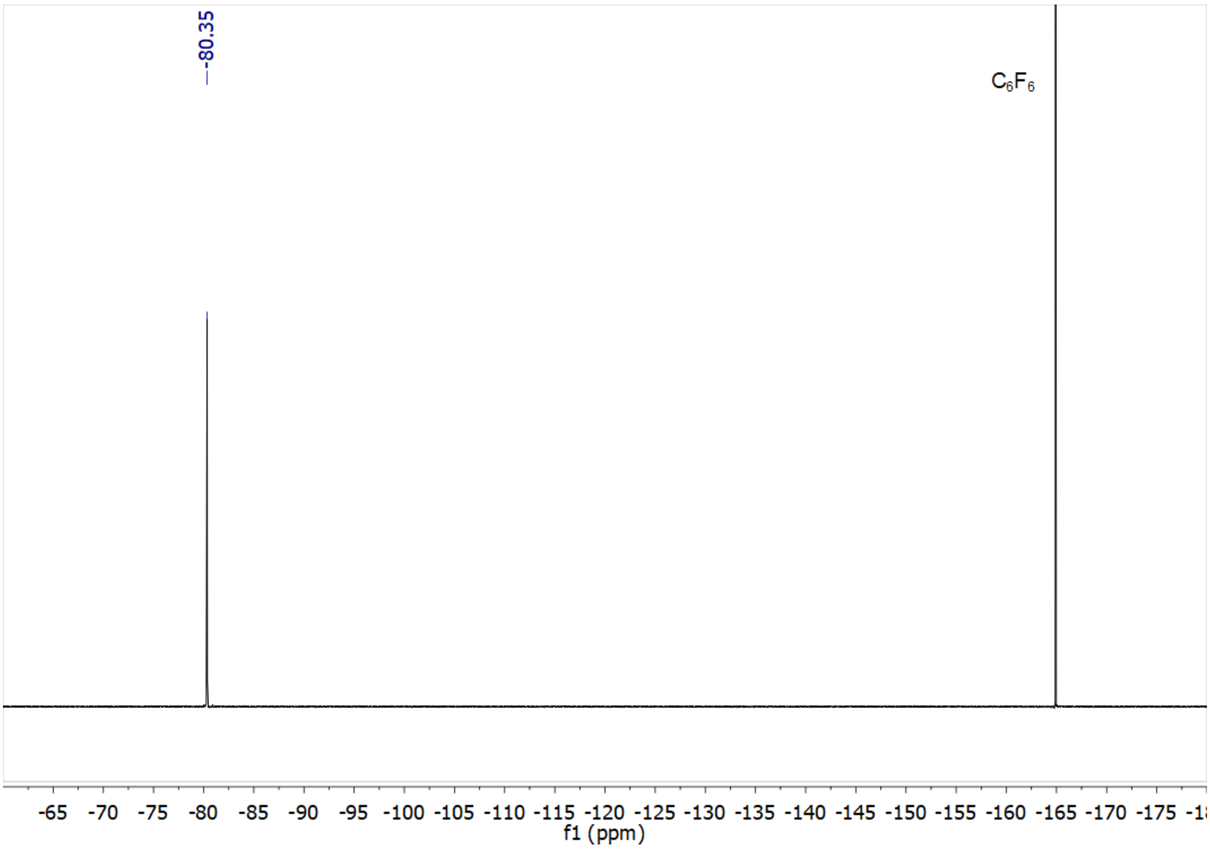

*Figure S66: ^{19}F NMR spectrum of **4**·(NTf$_2$)$_{16}$ (376 MHz, CD_3CN, 298 K).*

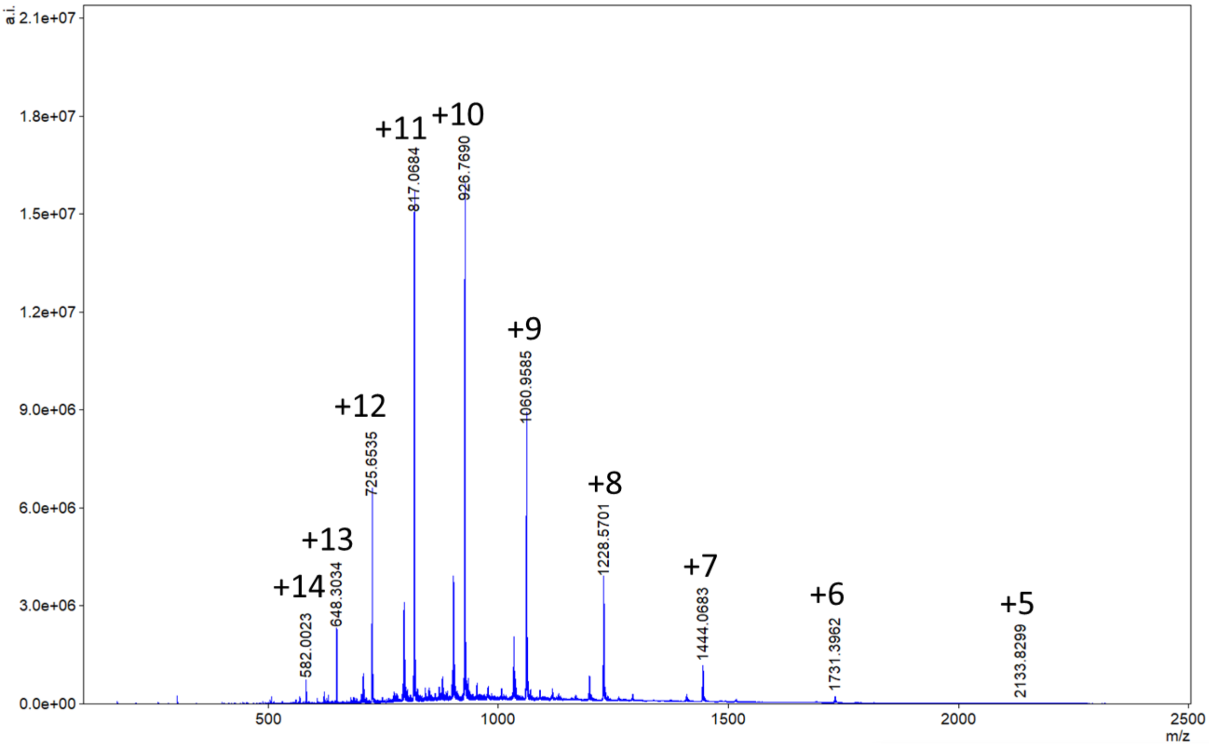

*Figure S67: High resolution ESI-MS spectrum of **4**·(NTf$_2$)$_{16}$.*

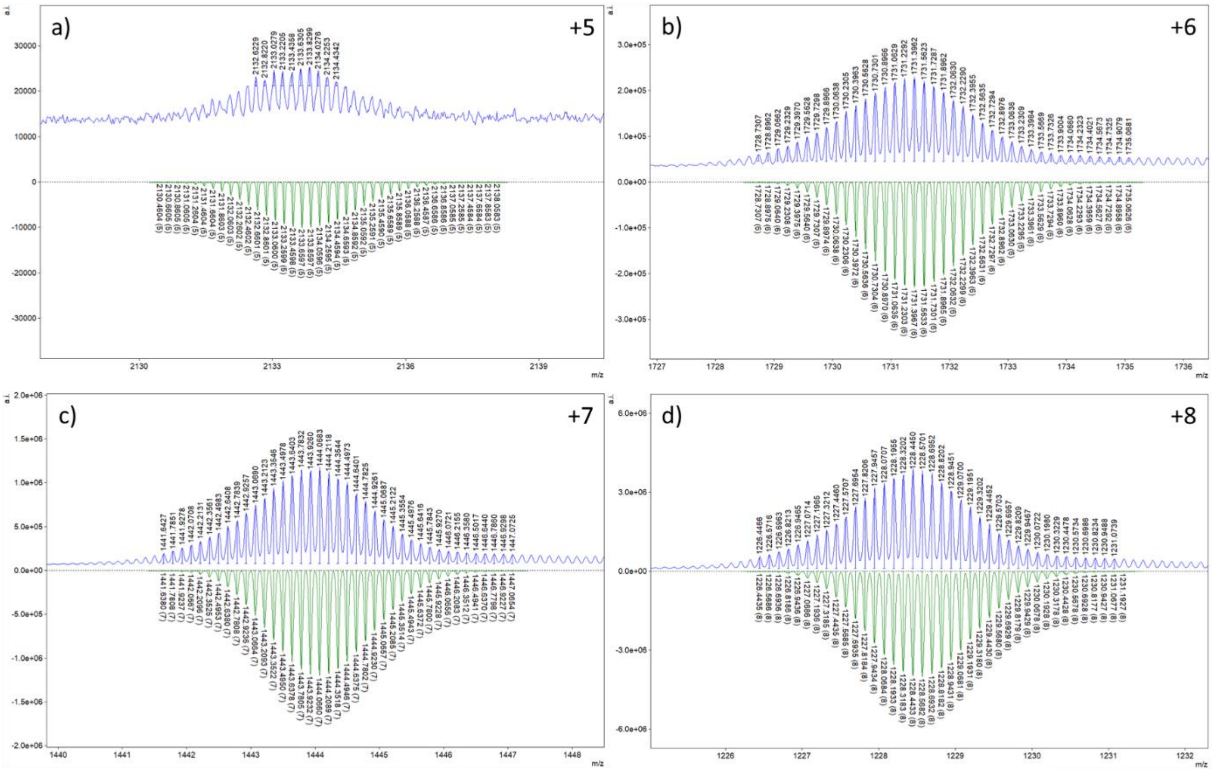

*Figure S68: Signals from the high-resolution ESI-mass spectrum for **4**·(NTf$_2$)$_{16}$. Experimental (blue) and calculated (green) signals for a) [**4**(NTf$_2$)$_{11}$]$^{5+}$ b) [**4**(NTf$_2$)$_{10}$]$^{6+}$ c) [**4**(NTf$_2$)$_9$]$^{7+}$, and d) [**4**(NTf$_2$)$_8$]$^{8+}$.*

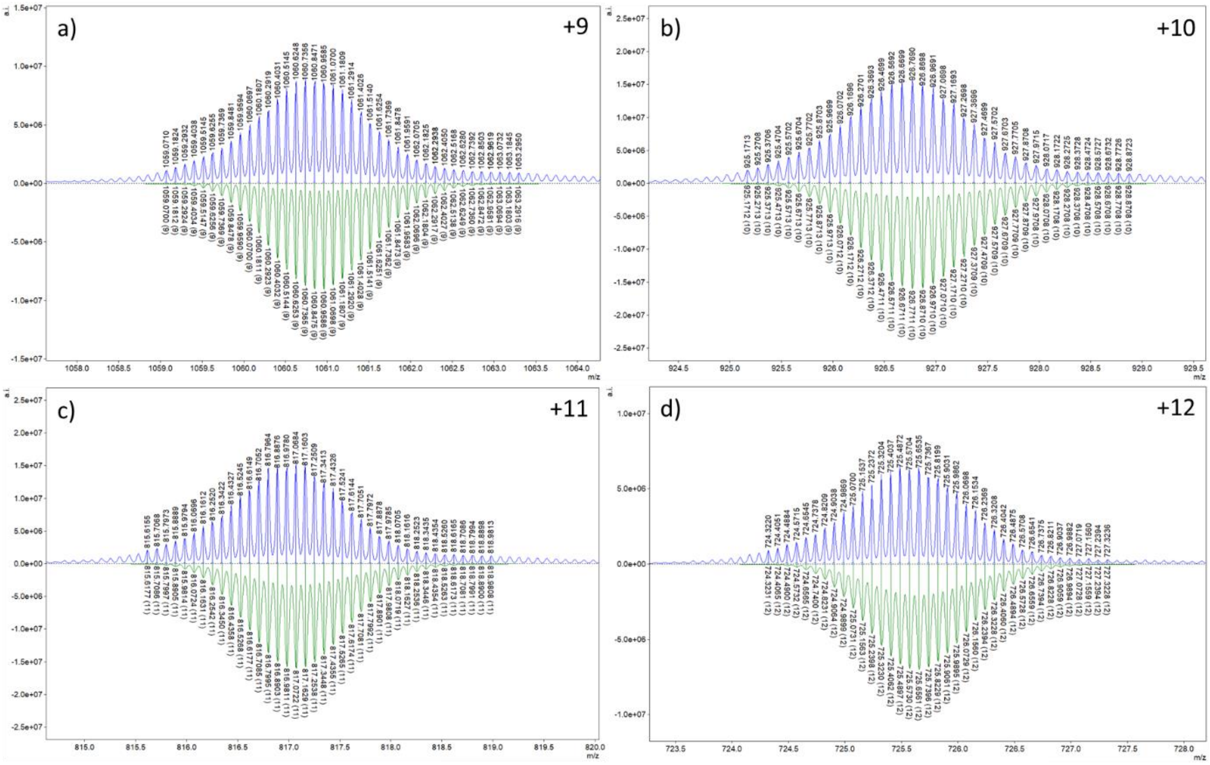

*Figure S69: Signals from the high-resolution ESI-mass spectrum for **4**·(NTf$_2$)$_{16}$. Experimental (blue) and calculated (green) signals for a) [**4**(NTf$_2$)$_7$]$^{9+}$ b) [**4**(NTf$_2$)$_6$]$^{10+}$ c) [**4**(NTf$_2$)$_5$]$^{11+}$, and d) [**4**(NTf$_2$)$_4$]$^{12+}$.*

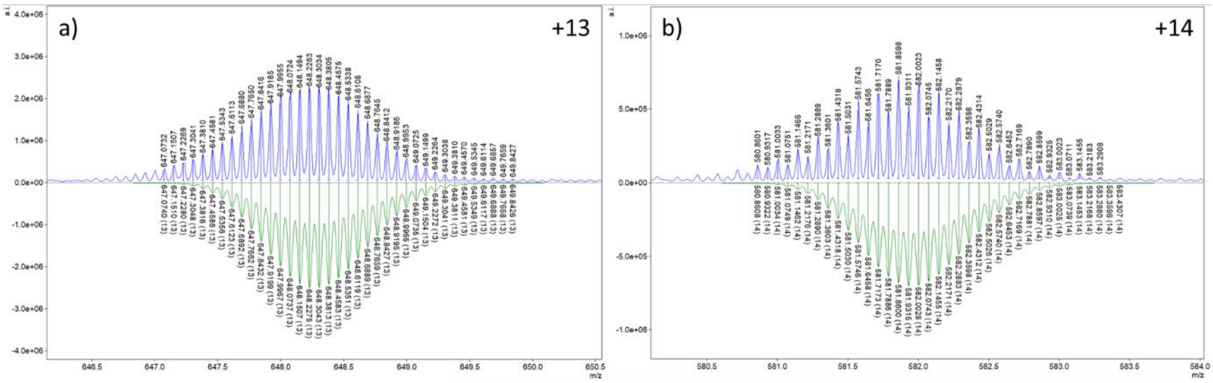

*Figure S70: Signals from the high-resolution ESI-mass spectrum for **4**·$(NTf_2)_{16}$. Experimental (blue) and calculated (green) signals for a) [**4**$(NTf_2)_3]^{13+}$, and b) [**4**$(NTf_2)_2]^{14+}$.*

4. Single-crystal X-ray Diffraction

Data were collected at Beamline I19 of Diamond Light Source employing silicon double crystal monochromated synchrotron radiation (0.6889 Å) with ω and *ψ* scans at 100(2) K.[5] Data integration and reduction were undertaken with Xia2.[6–8] Subsequent computations were carried out using the WinGX-32 and OLEX graphical user interfaces.[9,10] Multi-scan empirical absorption corrections were applied to the data using the AIMLESS[11] tool in the CCP4 suite.[12] The structures were solved by direct methods using SHELXT[13] then refined and extended with SHELXL.[14] In general, non-hydrogen atoms with occupancies greater than 0.5 were refined anisotropically. Carbon-bound hydrogen atoms were included in idealised positions and refined using a riding model. Disorder was modelled using standard crystallographic methods including constraints, restraints, and rigid bodies where necessary. Crystallographic data along with specific details pertaining to the refinement follow. Crystallographic data have been deposited with the CCDC (2362227-2362229).

*Table S1: Crystal data and structure refinement for **1**·$(SbF_6)_{16}$, **2**·$(PF_6)_{16}$, and **4**·$(BPh_4^F)_{16}$.*

Compound	**1**·$(SbF_6)_{16}$	**2**·$(PF_6)_{16}$	**4**·$(BPh_4^F)_{16}$
CCDC number	2362227	2362228	2362229
Empirical formula	C_{384} H_{312} N_{48} Zn_8, 16 (F_6 Sb)	C_{372} H_{240} N_{48} Zn_8, 16 (F_6 P)	C_{504} H_{336} N_{48} Zn_8, 16 (C_{24} H_{16} B F_4)
Formula weight	9893.76	8224.59	13845.98
Temperature [K]	100(2)	100(2)	100(2)
Crystal system	monoclinic	Cubic	trigonal
Space group	C2 (5)	-P 2 2 3 (200)	-R 3 (148)
a [Å]	39.815(2)	22.80040(10)	44.4981(10)
b [Å]	26.9653(6)	22.80040(10)	44.4981(10)
c [Å]	27.0164(9)	22.80040(10)	40.5472(18)
α [°]	90	90	90
β [°]	122.688(3)	90	90
γ [°]	90	90	120
Volume [$Å^3$]	24412.0(17)	11852.98(16)	69530(4)
Z	2	1	3
ρ_{calc} [g cm^{-3}]	1.346	1.152	0.992

μ [mm^{-1}]	1.238	0.497	0.247
F(000)	9744	4152	21456
Crystal size [mm^3]	0.050×0.040×0.030	0.04x0.03x0.03	0.04×0.035×0.03
Crystal colour	Light yellow	Yellow	Purple
Crystal shape	block	Block	Cube
Radiation	Synchrotron (λ = 0.6889 Å)	Synchrotron (λ = 0.6889 Å)	Synchrotron (λ = 0.6889 Å)
2ϑ range [°]	1.70 to 20.15	0.87 to 20.07	0.89 to 18.25
Index ranges	-39 ≤ h ≤ 39<br>-25 ≤ k ≤ 26<br>-26 ≤ l ≤ 27	-25 ≤ h ≤ 25<br>-25 ≤ k ≤ 25<br>-25 ≤ l ≤ 25	-40 ≤ h ≤ 40<br>-40 ≤ k ≤ 40<br>-36 ≤ l ≤ 36
Reflections collected	37673	49428	146372
Independent reflections	23242<br>R_{int} = 0.0978	3104<br>R_{int} = 0.0506	12136<br>R_{int} = 0.0907
Completeness to theta	Theta 20.148: 98.9 %	Theta 22.488: 100.0 %	Theta 18.248: 99.9 %
Absorption correction type	Empirical	Empirical	Empirical
Min. and max. transmission	0.9791701204349984<br>1.0	0.8667955638126491<br>1.0	0.938696291630515<br>1.0
Data/Restraints/Parameters	23242/5290/2147	3104/404/199	12136/1962/886
Goodness-of-fit on F^2	1.050	1.061	1.006
Final R indexes [I≥2σ(I)]	R_1 = 0.1424<br>wR_2 = 0.3643	R_1 = 0.1014<br>wR_2 = 0.3159	R_1 = 0.1434<br>wR_2 = 0.3765
Final R indexes [all data]	R_1 = 0.1579<br>wR_2 = 0.3809	R_1 = 0.1351<br>wR_2 = 0.3338	R_1 = 0.2199<br>wR_2 = 0.4022
Largest peak/hole [eÅ^{-3}]	1.447/−0.946	0.681/-0.593	0.421/−0.596

Specific refinement details for **1**·$(SbF_6)_{16}$ [+ solvent]

The crystals of **1**·$(SbF_6)_{16}$ were grown by diffusion of benzene into an acetonitrile solution of the **1**·$(NTf_2)_{16}$ complex containing excess potassium hexafluoroantimonate. The crystals employed immediately lost solvent after removal from the mother liquor and rapid handling prior to flash cooling in liquid nitrogen was required to collect data. Despite these measures and the use of synchrotron radiation few reflections at greater than 1.1 Å resolution were observed and the data were trimmed accordingly. The diffraction was broad, and the quality of the integration is less than ideal, hence the values of the R1, wR and wR2 factors are larger than for typical small molecule structures. Nevertheless, the quality of the data is easily sufficient to establish the connectivity of the structure. The asymmetric unit was found to contain one half of a Zn_8L_{12} assembly and associated counterions. The structure was refined in the chiral space group C2 as a racemic twin with the Flack parameter refining to 0.47(4).

Due to the limited resolution of the data and high degree of thermal motion in the toluidine groups, bond length and angle restraints were required to obtain a reasonable model for the organic parts of the structure. The GRADE program[15] was therefore employed, using the GRADE Web Server,[16] to generate a full set of bond distance and angle restraints (DFIX, DANG, FLAT) for the organic ligands. Thermal parameter restraints (SIMU, ISOR, RIGU) were also applied to all atoms except for zinc and antimony to facilitate anisotropic refinement.

One of the hexafluoroantimonate anions was modelled as disordered over two sites and another is disordered around a special position. Bond length restraints were applied to all anions and the fluorine atoms of each anion were constrained to have equal thermal parameters. All fluorine atoms were refined isotropically. The occupancies of all located hexafluoroantimonate anions were allowed to freely refine, resulting in a discrepancy of ca. 8.5 counterions per Zn_8L_{12} assembly (included as hexafluoroantimonate in the formula).

The remaining anions and solvent within the lattice were significantly disordered and despite numerous attempts at modelling, including with rigid bodies, no satisfactory model for the electron-density associated with them could be found. Consequently the SQUEEZE[17] function of PLATON[18] was employed to remove the contribution of the electron density associated with these remaining anions and further highly disordered solvent, which gave a potential solvent accessible void of 8629 Å^3 per unit cell (a total of approximately 3973 electrons). The diffuse solvent molecules could not be assigned to acetonitrile or benzene and were also not included in the formula. Consequently, the molecular weight and density given above are underestimated.

CheckCIF gives one A and three B level alerts. These alerts all result from the limited resolution of the data (low sine(theta_max)/wavelength, poor data/parameter ratio, low bond precision) and poor diffractions properties of the crystals (high wR2 value) as described above.

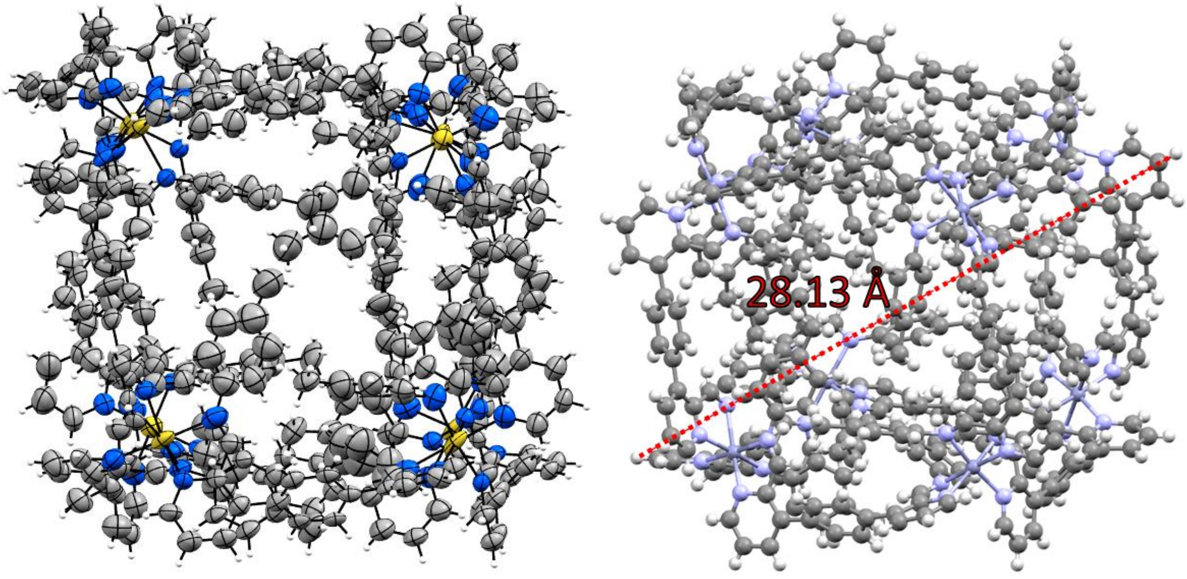

*Figure S71: Left: Ortep-style plot of the cationic portion of the crystal structure of **1**·$(SbF_6)_{16}$ [+ solvent], showing thermal ellipsoids at a probability of 50%. The asymmetric unit contains one half of a Zn_8L_{12} assembly. Right: Determination of the solvent-free diameter.*

Specific refinement details for **2**·$(PF_6)_{16}$ [+ solvent]

The crystals of **2**·$(PF_6)_{16}$ were grown by diffusion of diethylether into an acetonitrile solution of the **2**·$(NTf_2)_{16}$ complex containing excess TBA(PF_6). The crystals employed immediately lost solvent after removal from the mother liquor and rapid handling prior to flash cooling in liquid nitrogen was required to collect data. Despite these measures and the use of synchrotron radiation few reflections at greater than 0.9 Å resolution were observed and the data were trimmed accordingly. The diffraction was broad and the quality of the integration is less than ideal hence the values of the R1, wR and wR2 factors are larger than for typical small molecule structures. Nevertheless, the quality of the data is clearly sufficient to establish the connectivity of the structure. The asymmetric unit was found to contain 1/24 of a $Zn_8L'_6$ assembly and associated counterions. Due to the limited resolution of the data

and high degree of thermal motion of the atoms of the counteranions, bond length and angle restraints were required in order to obtain a reasonable model for the organic parts of the structure. Thermal parameter restraints (SIMU, RIGU) were also applied to all atoms except for zinc to facilitate anisotropic refinement. Due to high thermal motion of the counteranion, additionally ISOR and DELU were applied to phosphorus and fluorine atoms.

The occupancy of the counteranions was determined by free variables (FVAR). The remaining anions (4.9 per Zn_8L_{12} assembly) and solvent within the lattice were significantly disordered and despite numerous attempts at modelling, including with rigid bodies, no satisfactory model for the electron-density associated with them could be found. Consequently the solvent mask of OLEX based on BYPASS[17] was employed to remove the contribution of the electron density associated with these remaining anions and further highly disordered solvent, which gave a potential solvent accessible void of 3280 Å^3 per unit cell (a total of approximately 672 electrons). The diffuse solvent molecules could not be assigned to acetonitrile or diethylether and were also not included in the formula. Consequently, the molecular weight and density given above are underestimated.

CheckCIF gives one B level alert, which results from the limited resolution of the data.

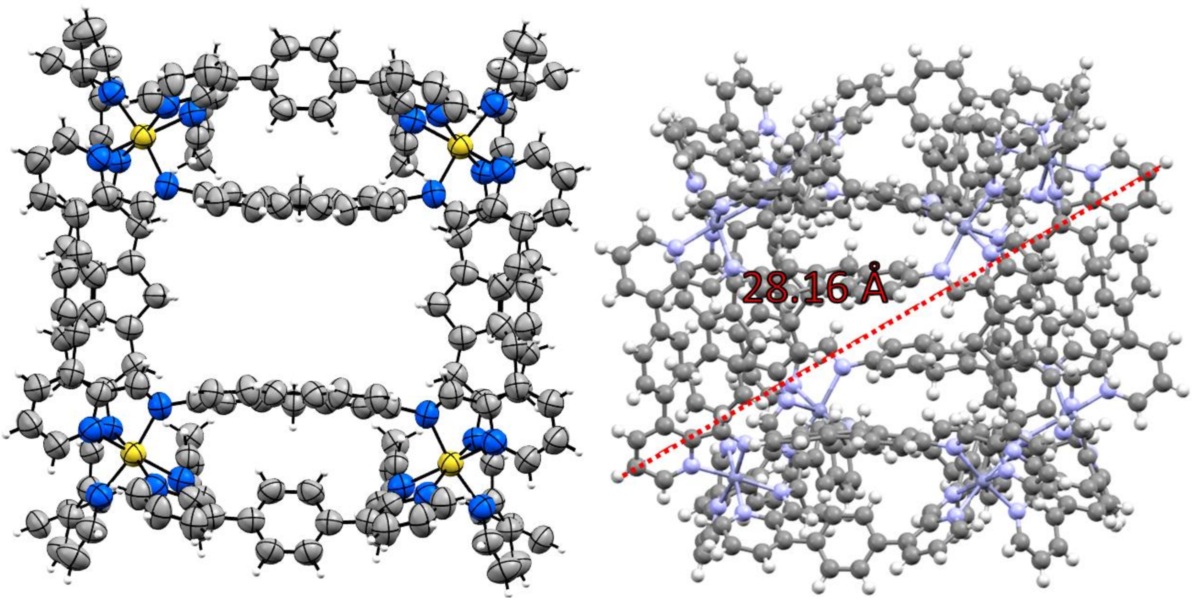

*Figure S72: Left: Ortep-style plot of the cationic portion of the crystal structure of **2**·$(PF_6)_{16}$ [+ solvent], showing thermal ellipsoids at a probability of 50%. The asymmetric unit contains 1/24 of a $Zn_8L'_6$ assembly. Right: Determination of the solvent-free diameter.*

Specific refinement details for **4**·$(BPh_4^F)_{16}$ [+ solvent]

The crystals of **4**·$(BPh_4^F)_{16}$ were grown by diffusion of benzene into an acetonitrile solution of the **4**·$(NTf_2)_{16}$ complex containing excess Na(BPh_4^F). The crystals employed immediately lost solvent after removal from the mother liquor and rapid handling prior to flash cooling in liquid nitrogen was required to collect data. Despite these measures and the use of synchrotron radiation, few reflections at greater than 1.1 Å resolution were observed and the data were trimmed accordingly. The diffraction was broad and the quality of the integration is less than ideal, hence the values of the R1, wR and wR2 factors are larger than for typical small molecule structures. Nevertheless, the quality of the data is far more than sufficient to establish the connectivity of the structure. The asymmetric unit was found to contain 1/6 of a $Zn_8L'''_6$ assembly and associated counterions. Due to the limited resolution of the data and high degree of thermal motion in the phenyl groups of the counteranions, bond length and angle restraints were required in order to obtain a reasonable model for the organic parts of the

structure. Thermal parameter restraints (SIMU, RIGU) were also applied to all atoms except for zinc and antimony to facilitate anisotropic refinement. Due to high thermal motion of the phenyl rings of BPh_4^F, additionally ISOR and DELU were applied to the carbons and fluorines.

The remaining anions (10 per Zn_8L_{12} assembly) and solvent within the lattice were significantly disordered and despite numerous attempts at modelling, including with rigid bodies no satisfactory model for the electron-density associated with them could be found. Consequently the solvent mask of OLEX based on BYPASS[17] was employed to remove the contribution of the electron density associated with these remaining anions and further highly disordered solvent, which gave a potential solvent accessible void of 39233 Å^3 per unit cell (a total of approximately 8521 electrons). The diffuse solvent molecules could not be assigned to acetonitrile or benzene and were also not included in the formula. Consequently, the molecular weight and density given above are underestimated.

CheckCIF gives two A and six B level alerts. Most alerts result from the limited resolution of the data (low sine(theta_max)/wavelength, poor data/parameter ratio, low bond precision) and poor diffraction properties of the crystals (high wR2 value) as described above. One alert results from a short contact between the phenyl rings of the counteranions featuring a high degree of thermal motion.

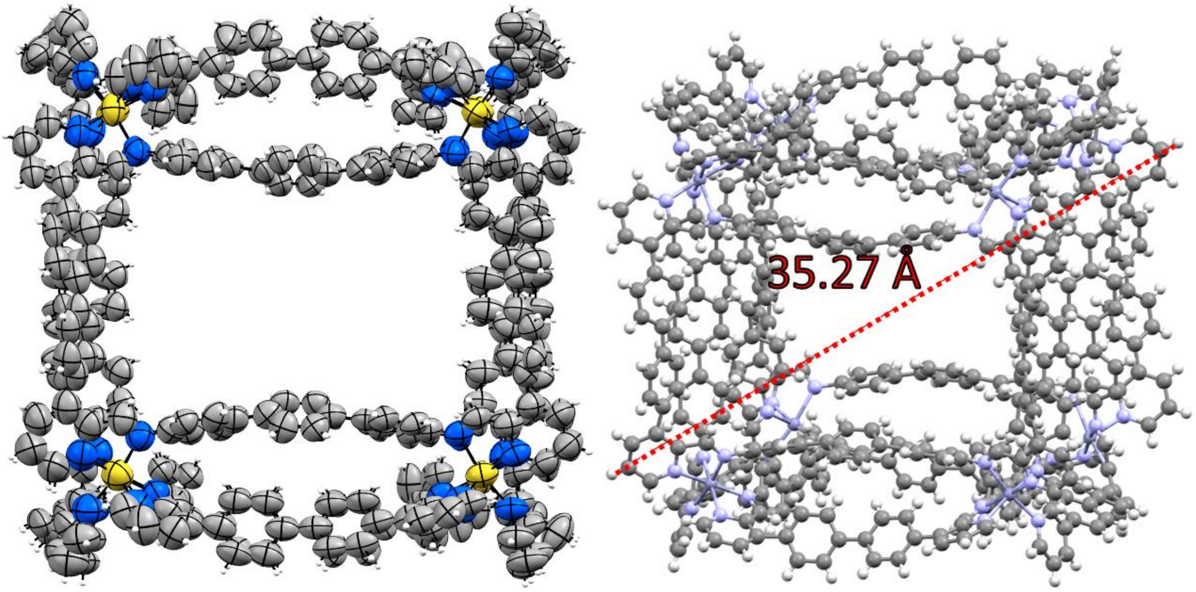

*Figure S73: Left: Ortep-style plot of the cationic portion of the crystal structure of **4**·$(BPh_4^F)_{16}$ [+ solvent], showing thermal ellipsoids at a probability of 50%. The asymmetric unit contains 1/6 of a $Zn_8L'''_6$ assembly. Right: Determination of the solvent-free diameter.*

5. Conversion from **1** to **2**

NMR and UV-vis studies

Experimental details:

Conversion experiments were performed in NMR tubes using a 0.4 mM CD_3CN solution of the respective cage. Either 2,7-diaminofluorene (12 eq) or *p*-toluidine (24 eq) was added, and after heating for 20 h at 70°C, a ^{1}H NMR spectrum was taken. As the conversion from **1** to **2** was already complete after addition of 12 equiv 2,7-diaminofluorene, the product was purified by washing three times with 15 mL Et_2O. As no conversion from **2** to **1** was observed upon addition of 24 equiv of *p*-toluidine, further equivalents of *p*-toluidine were added to the same solution and the solution was heated again

for 20 h at 70°C in a stepwise manner. The number of equivalents listed below correspond to the total quantity added to the solution.

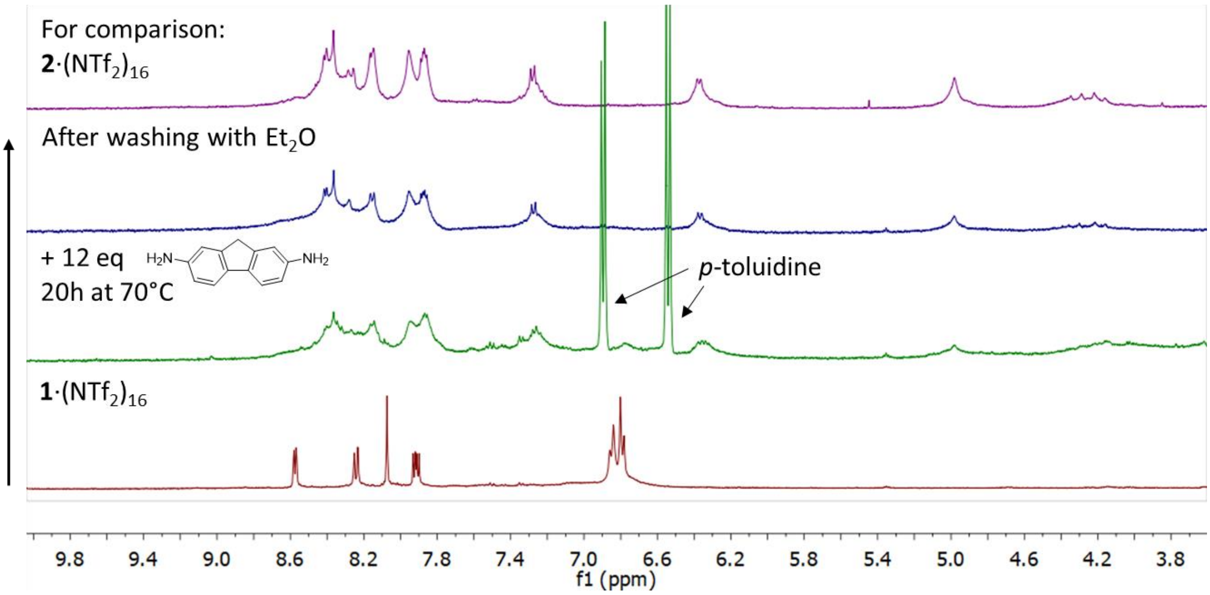

*Figure S74: ^{1}H NMR spectra showing conversion from **1** to **2** upon addition of 12 equiv 2,7-diaminofluorene to **1**·$(NTf_2)_{16}$ (400 MHz, CD_3CN, 298 K). Full conversion of **1** to **2** was observed upon addition of 12 equiv of 2,7-diaminofluorene.*

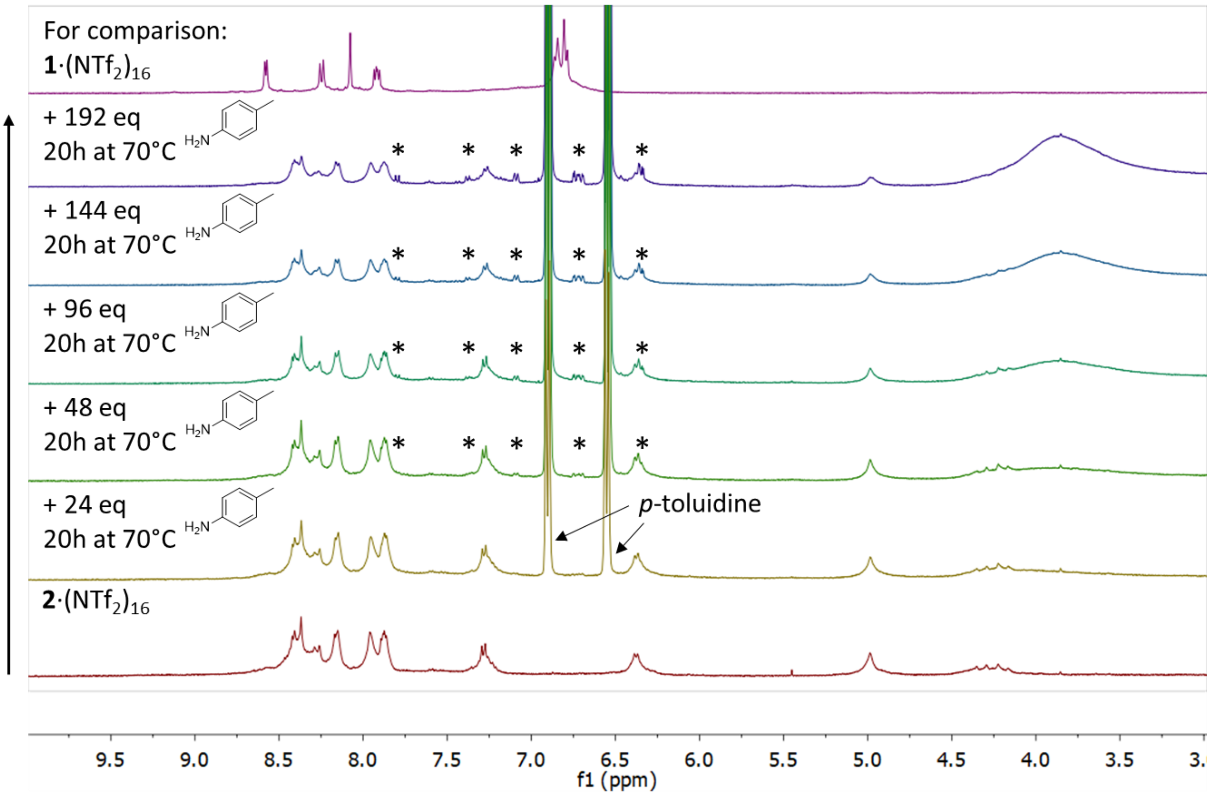

*Figure S75: ^{1}H NMR spectra of titration experiment of **2**·$(NTf_2)_{16}$ with p-toluidine (400 MHz, CD_3CN, 298 K). The number of equivalents corresponds to the total equivalent quantity added to the solution. No conversion of **2** to **1** was observed even upon addition of 192 equiv of p-toluidine. We note the formation of an unidentified product after addition of more than 48 equiv of p-toluidine (marked with asterisks).*

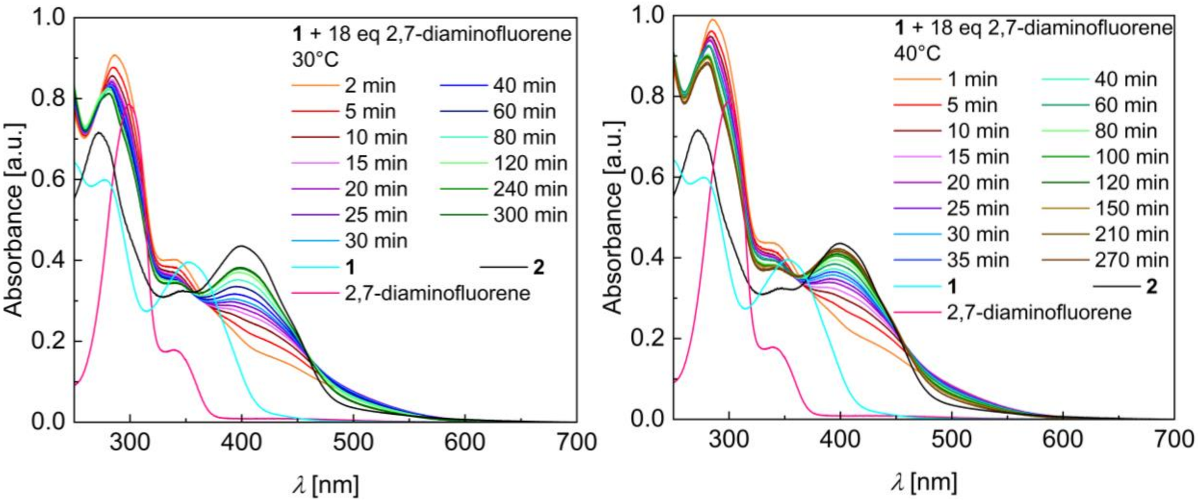

*Figure S76: UV-vis spectra showing time-dependent conversion from **1** to **2** upon addition of 18 equiv 2,7-diaminofluorene to a 0.04 mM **1**·$(NTf_2)_{16}$ solution at 30°C (left) and 40°C (right).*

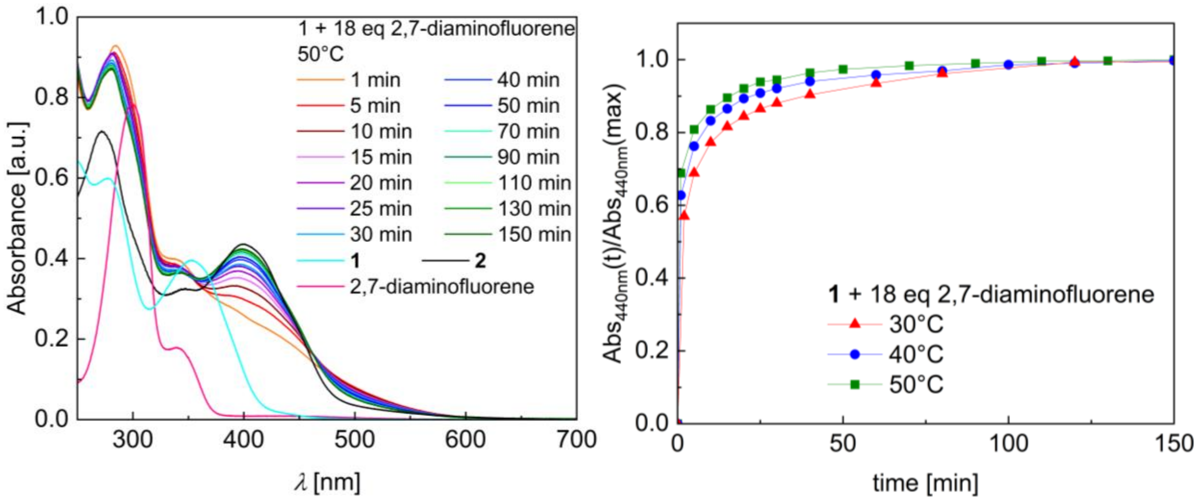

*Figure S77: UV-vis spectra showing time-dependent conversion from **1** to **2** upon addition of 18 equiv 2,7-diaminofluorene to a 0.04 mM **1**·$(NTf_2)_{16}$ solution at 50°C (left). Plot of the absorbance change (Abs(t)/Abs(max)) at 440 nm vs. time at different temperatures (right).*

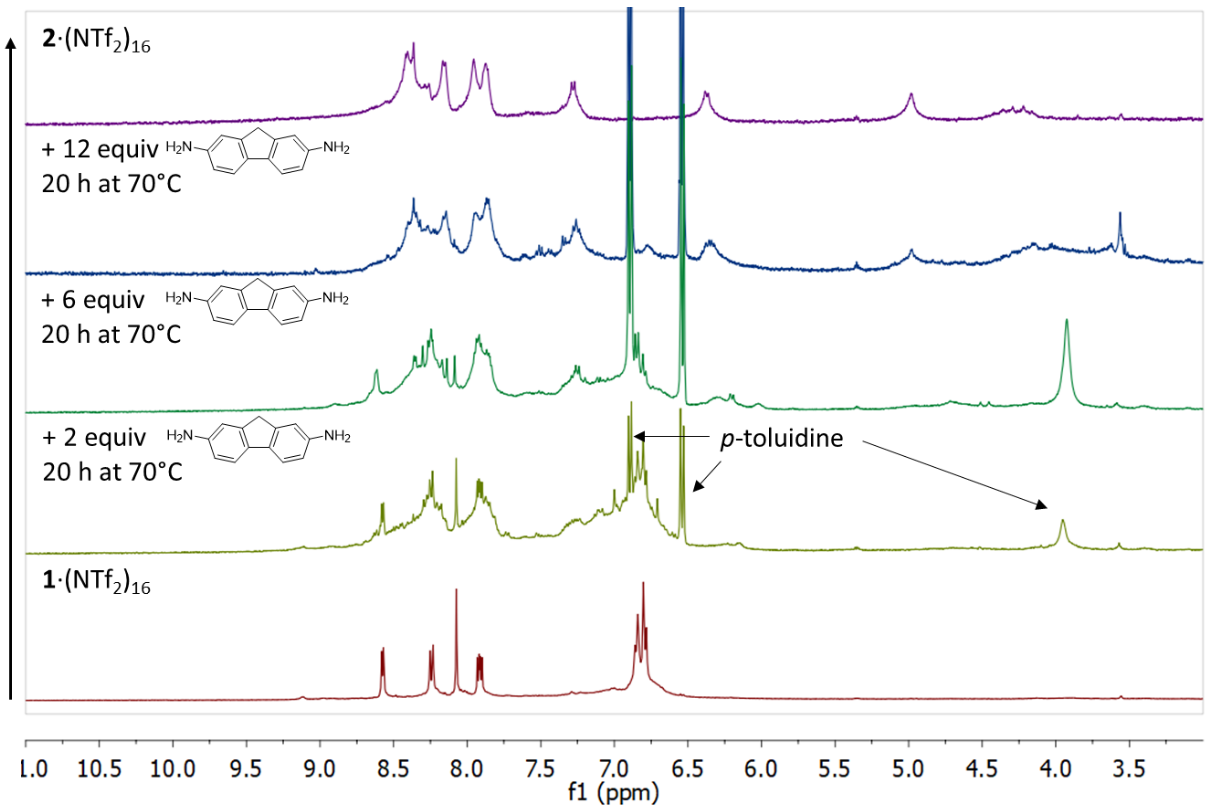

*Figure S78: ^{1}H NMR spectra of **1**·(NTf$_2$)$_{16}$ and upon successive addition of various equiv of 2,7-diaminofluorene (500 MHz, CD_3CN, 298 K). Upon addition of 2 and 6 equiv of 2,7-diaminofluorene a mixture of cage **1**, cage **2**, and an undefined species was observed. Upon addition of 12 equiv of 2,7-diaminofluorene, only cage **2** and small amounts of impurities, which could be washed off, were observed. We note that neither an aldehyde signal nor signals corresponding to free 2,7-diamiofluorene were observed. In contrast, free p-toluidine was observed.*

Computational Studies

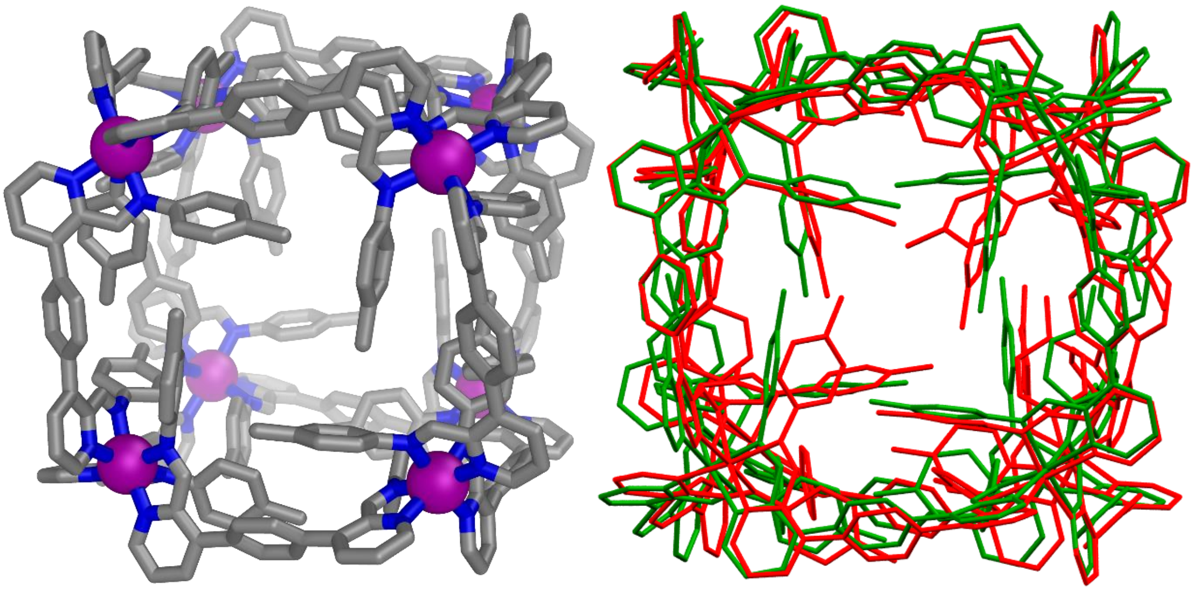

*Figure S79: GFN2-xTB-optimized structure of **1**; protons are omitted for clarity (left). Calculations were performed without inclusion of counteranions or solvent molecules. Comparison of the crystal structure (red) and the GFN2-xTB-optimized structure (green) of **1**, showing that the crystal and GFN2-xTB-optimized structure are very similar.*

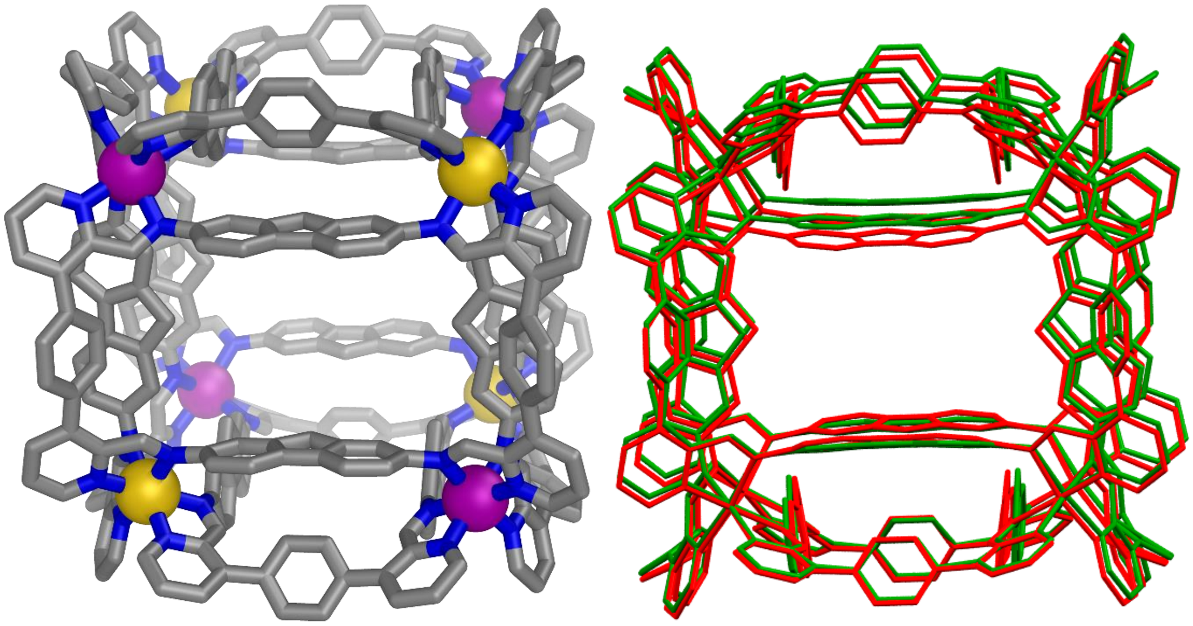

*Figure S80: GFN2-xTB-optimized structure of **2**; protons are omitted for clarity (left). Calculations were performed without inclusion of counteranions or solvent molecules. Comparison of the crystal structure (red) and the GFN2-xTB-optimized structure (green) of **2**, showing that the crystal and GFN2-xTB-optimized structure are very similar.*

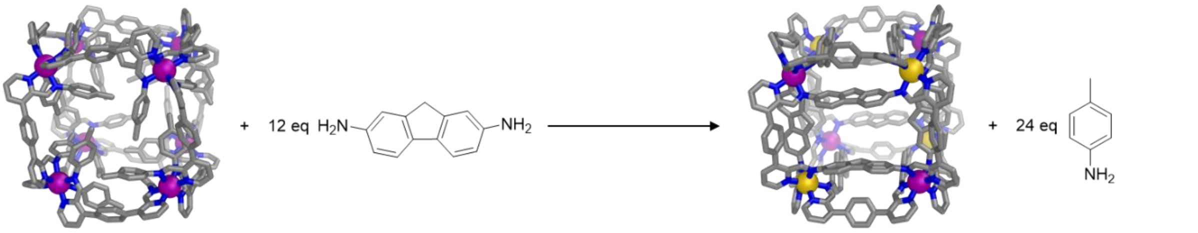

1 **2,7-diaminofluorene (a)** **2** ***p*-toluidine (b)**

*Figure S81: Reaction pathway of transition from **1** to **2** upon addition of 12 equiv 2,7-diaminofluorene.*

Semi-empirical quantum mechanical calculations were carried out using OPTIM program,[19] which contains a wide variety of geometry optimization tools for locating stationary points on potential energy surfaces and calculating reaction pathways. Geometry optimizations were performed on structures **1**, 2,7-diaminofluorene (**a**), **2**, and *p*-toluidine (**b**) using the OPTIM interface[20] to the XTB program, which was used to calculate the energy and gradient at the GFN2-xTB level of theory.[21–23] These calculations were followed by optimisations at the GFN2-xTB level of theory with CREST through the tblite package.[24,25] Input structures for the two cages (**1** and **2**) were created by deleting the counterions from their corresponding crystal structures. A charge of 16 and 0 unpaired electrons were assigned to the cage structures **1** and **2**, whereas a charge of 0 and 0 unpaired electrons were assigned to the 2,7-diaminofluorene (**a**) and *p*-toluidine (**b**). For these calculations the 'ohess' runtype was used. Vibrational analysis of the optimized structures of **a** and **b** showed no imaginary frequencies, indicating well converged minima in the gas-phase. Similar analyses of the cages exhibited one small remaining imaginary frequency for cage **2** (-43 cm^{-1}) and eight remaining imaginary frequencies for cage **1** (> -100 cm^{-1}). (Note that imaginary frequencies are conventionally reported as "negative" for convenience.) The small negative frequencies were reversed in sign for the purpose of calculating free energy contributions, following standard practice. Calculations with implicit acetonitrile solvation did not lead to physically correct minima for cage **1**, but were successful for **2**, 2,7-diaminofluorene (**a**),

and *p*-toluidine (**b**), and are therefore omitted. Using the gas phase results, the total energy change ΔE^{GFN2} for the reaction shown in Figure S54 was calculated using equation 1.

$$\Delta E^{\text{GFN2}} = E_{\mathbf{2}}^{\text{GFN2}} + 24E_{\boldsymbol{b}}^{\text{GFN2}} - E_{\mathbf{1}}^{\text{GFN2}} - 12E_{\boldsymbol{a}}^{\text{GFN2}} \quad \text{Eq. 1}$$

Vibrational analyses of **1**, **2**, 2,7-diaminofluorene (**a**), and *p*-toluidine (**b**) were performed at the GFN2-xTB level using CREST, where a modified and scaled rigid-rotor-harmonicoscillator approximation (msRRHO) was employed.[26,27] Using these results, the reaction Gibbs free energy ΔG^{GFN2} of the reaction was calculated using equation 2-4.

$$\Delta G^{\text{GFN2}} = G_{\mathbf{2}}^{\text{GFN2}} + 24G_{\boldsymbol{b}}^{\text{GFN2}} - G_{\mathbf{1}}^{\text{GFN2}} - 12G_{\boldsymbol{a}}^{\text{GFN2}} \quad \text{Eq. 2}$$

$$G_{\text{i}\in\mathbf{1},\mathbf{a},\mathbf{2},\mathbf{b}}^{\text{GFN2}} = E_{i}^{\text{GFN2}} + \delta G_{\text{rrho},i}^{\text{GFN2}} \quad \text{Eq. 3}$$

$$\delta G_{\text{rrho},i} = [H(0) - H(T)]_i + ZPVE_i - TS_i \quad \text{Eq. 4}$$

Here, G_i is the Gibbs free energy of structure *i, $H(T)$* is the enthalpy of structure *i* at temperature T, $ZPVE_i$ is the zero-point vibrational energy of structure *i* and S_i is the entropy of structure *i*. $H(T)$ and TS_i values calculated at the GFN2-xTB level for each structure can be found in table 1 (T = 298.15 K).

The reaction enthalpy ΔH^{GFN2} was calculated by applying the thermodynamic correction $\Delta\delta\text{H}^{\text{GFN2}}$ to ΔE^{GFN2} as shown in equation 5 and 6. were calculated using equation 5 and 6.

$$\Delta H^{\text{GFN2}} = \Delta E^{\text{GFN2}} + \Delta\delta\text{H}^{\text{GFN2}} \quad \text{Eq. 5}$$

$$\Delta\delta\text{H}^{\text{GFN2}} = H(T)_{\text{products}}^{\text{GFN2}} - H(T)_{\text{reactants}}^{\text{GFN2}} \quad \text{Eq. 6}$$

$$= H(T)_{\mathbf{2}}^{\text{GFN2}} + 24H(T)_{\boldsymbol{b}}^{\text{GFN2}} - H(T)_{\mathbf{1}}^{\text{GFN2}} - 12H(T)_{\boldsymbol{a}}^{\text{GFN2}}$$

The reaction entropy multiplied by the temperature ($T\Delta S^{\text{GFN2}}$) was calculated from the thermodynamic correction $\Delta\delta\text{S}^{\text{GFN2}}$ using equation 7 and 8.

$$T\Delta S^{\text{GFN2}} = -\,\text{T}\Delta\delta\text{S}^{\text{GFN2}} \quad \text{Eq. 7}$$

$$\text{T}\Delta\delta\text{S}^{\text{GFN2}} = TS_{\text{products}}^{\text{GFN2}} - TS_{\text{reactants}}^{\text{GFN2}} \quad \text{Eq. 8}$$

$$= TS_{\mathbf{2}}^{\text{GFN2}} + 24TS_{\boldsymbol{b}}^{\text{GFN2}} - TS_{\mathbf{1}}^{\text{GFN2}} - 12TS_{\boldsymbol{a}}^{\text{GFN2}}$$

Single-point energy (SPE) calculations at the density functional theory (DFT) level of theory were performed via ORCA on the GFN2-xTB optimised structures of **1**, **a**, **2** and **b**.[28] Calculations were performed using the r^{2}scan-3c method.[29–33] Additionally, the ORCA calculation was performed with energy gradients (*EnGrad*), tight self-consistent field (SCF) convergence criteria (*TightSCF*), and the *defgrid3* integration grid. The maximum memory allocation was set to 10,000 MB *(%maxcore 10000*). A charge of 16 and a spin multiplicity of 1 were applied to the input structures. This method, where a SPE calculation is performed on GFN2-xTB optimised structures, is also called '*r^{2}SCAN-3c//GFN2-xTB'* and is further referred to as '*DFT//GFN2'*.[34,35]

The reaction Gibbs free energy (ΔG), enthalpy (ΔH) and entropy (ΔS) at the DFT//GFN2 level of theory for the reaction were calculated using equation 9-14, see Table 3. In equation 11-13, the thermodynamic corrections calculated at the GFN2-xTB level ($\delta G_{\text{rrho},i}^{\text{GFN2}}$, $\Delta\delta\text{H}^{\text{GFN2}}$ and $\text{T}\Delta\delta\text{S}^{\text{GFN2}}$) were applied to the total energy values obtained with r^{2}SCAN-3c. A value of 131.70 kcal/mol was obtained

for $\mathrm{T}\Delta S^{\mathrm{DFT//GFN2}}$ at T = 298.15 K, revealing a remarkably large entropic contribution to the stabilization caused by exchanging amine **a** with diamine **b**.

$$\Delta E^{\mathrm{DFT//GFN2}} = E_{\mathbf{2}}^{\mathrm{DFT//GFN2}} + 24E_{\boldsymbol{b}}^{\mathrm{DFT//GFN2}} - E_{\mathbf{1}}^{\mathrm{DFT//GFN2}} - 12E_{\boldsymbol{a}}^{\mathrm{DFT//GFN2}} \quad \text{Eq. 9}$$

$$\Delta G^{\mathrm{DFT//GFN2}} = G_{\mathbf{2}}^{\mathrm{DFT//GFN2}} + 24G_{\boldsymbol{b}}^{\mathrm{DFT//GFN2}} - G_{\mathbf{1}}^{\mathrm{DFT//GFN2}} - 12G_{\boldsymbol{a}}^{\mathrm{DFT//GFN2}} \quad \text{Eq. 10}$$

$$G_{\mathrm{i}\in\mathbf{1},\mathbf{a},\mathbf{2},\mathbf{b}}^{\mathrm{DFT//GFN2}} = E_{i}^{\mathrm{DFT//GFN2}} + \delta G_{\mathrm{rrho},i}^{\mathrm{GFN2}} \quad \text{Eq. 11}$$

$$\Delta H^{\mathrm{DFT//GFN2}} = \Delta E^{\mathrm{DFT//GFN2}} + \Delta\delta\mathrm{H}^{\mathrm{GFN2}} \quad \text{Eq. 12}$$

$$\Delta G^{\mathrm{DFT//GFN2}} = \Delta H^{\mathrm{DFT//GFN2}} + \mathrm{T}\Delta\delta\mathrm{S}^{\mathrm{GFN2}} \quad \text{Eq. 13}$$

$$\mathrm{T}\Delta S^{\mathrm{DFT//GFN2}} = -T\Delta\delta\mathrm{S}^{\mathrm{GFN2}} \quad \text{Eq. 14}$$

*Table S2: Thermodynamic properties of compounds **1**, **a**, **2** and **b** calculated at the GFN2-xTB level and total energies of their minima calculated with both GFN2-xTB and r^{2}SCAN-3c//GFN2-xTB. These values are given in hartree units and correspond to a temperature of T = 298.15 K.*

Compound	$\boldsymbol{E}^{\mathbf{GFN2}}$	$\boldsymbol{H(T)}^{\mathbf{GFN2}}$	$\boldsymbol{TS}^{\mathbf{GFN2}}$	$\boldsymbol{E}^{\mathbf{DFT//GFN2}}$
**1**	-1116.744721	6.214060	0.865944	-31673.776739
**a**	-39.807012	0.227440	0.050059	-611.996625
**2**	-1054.558570	5.337410	0.693688	-31173.483701
**b**	-22.486594	0.149443	0.040951	-326.836319

Table S3: Thermodynamic properties of the reaction at the GFN2-xTB and r^{2}SCAN-3c//GFN2-xTB levels of theory. These values are given in kcal/mol and correspond to a temperature of T = 298.15 K.

Method	$\Delta\boldsymbol{E}$	$\Delta\delta\mathbf{H}$	$\mathbf{T}\Delta\delta\mathbf{S}$	$\Delta\boldsymbol{H}$	$\Delta\boldsymbol{G}$	$\boldsymbol{T}\Delta\boldsymbol{S}$
**GFN2-xTB**	120.51	-12.12	-131.70	108.40	-23.39	131.70
**r2SCAN-3c //GFN2-xTB**	113.51	x	x	101.40	-30.39	131.70

Geometry optimisations at the GFN2-xTB level of theory with CREST through the tblite package were performed on cage structures **3** and **4** similarly to **1** and **2**. MM3 optimized geometries were used as the input structures and a charge of 16 and 0 unpaired electrons were assigned. Well converged minima were obtained for the gas-phase. Vibrational analysis of the optimized structure of **3** revealed one small remaining negative frequency (-30 cm^{-1}), whereas vibrational analysis of structure **4** showed five small remaining negative frequencies (> -100 cm^{-1}). Adjusting the optimization level to 'extreme' did not lead to removal of these frequencies, and they probably result from the projection of translational and rotational degrees of freedom from the second derivative matrix, and from remaining noise in the potential.

Quantification of molecular rigidity

Although various measures have been proposed to assess the rigidity of molecular systems, mainly in the context of biomolecules,[36] no *de facto* standard method exists for this purpose. A physical quantity associated with the flexibility is the configurational entropy of the system.[27] Unfortunately, sampling

of the entropy requires tremendous computational effort, which makes it impractical for investigating systems with over a few hundred atoms, like **1** or **2**.

Hence, in an alternative approach, we quantify the molecular flexibility of the **1** and **2** system via the time evolution of these systems at finite temperature. Starting from crystal structure cut-outs, initial minima were obtained at the GFN2-xTB level of theory[21,23] using the L-BFGS algorithm[37,38] implemented in the OPTIM program.[39] These structures were used as input for *NVT* molecular dynamics (MD) simulations as implemented in CREST program,[24,25] employing a time-step of 1 fs and a target temperature of 300 K, adjusted via a simple Berendsen thermostat. The overall simulation length was set to 50 ps for GFN2-xTB and 0.5 ns for GFN-FF.[40] Unfortunately, implicit solvation effects could not be included for **1**, since they led to a structural collapse (dissociating hydrogen atoms) for both GFN-FF and GFN2-xTB. Hence, all calculations were formally conducted for the gas-phase to avoid such issues. Furthermore, translational and rotational degrees of freedom were projected out in all simulations. Three quantities allow us to judge the molecular rigidity from the simulation: First, the integrated path lengths, which quantify the cumulative molecular displacement from the start to the end of the trajectory. Second, the average Cartesian root-mean-square-deviation (RMSD) of the MD snapshots with respect to the GFN2-xTB optimized input structure, providing a simple measure of total average deformation. And lastly, the fluctuation of total energy (E_{pot} + E_{kin}), obtained as the standard deviation (SD) of the average total energy over the entire simulation, effectively quantifying the magnitude of atom movement in between time steps. The integrated path length and Cartesian RMSD include only contributions from the heavier elements (C, N, Zn).

*Table S4: Comparison of molecular rigidity for the **1** and **2** systems based on the integrated path length [Å], average Cartesian RMSD [Å], and total energy fluctuation [kcal/mol]. Higher values indicate a higher molecular flexibility.*

		GFN-FF	GFN2-xTB
**1**	Integrated path length [Å]	3360.62	141.49
	Average RMSD [Å]	6.1270	0.5560
	Energy fluctuation [kcal/mol]	45.74	19.57
**2**	Integrated path length [Å]	3043.94	126.41
	Average RMSD [Å]	0.6503	0.2746
	Energy fluctuation [kcal/mol]	10.30	8.69

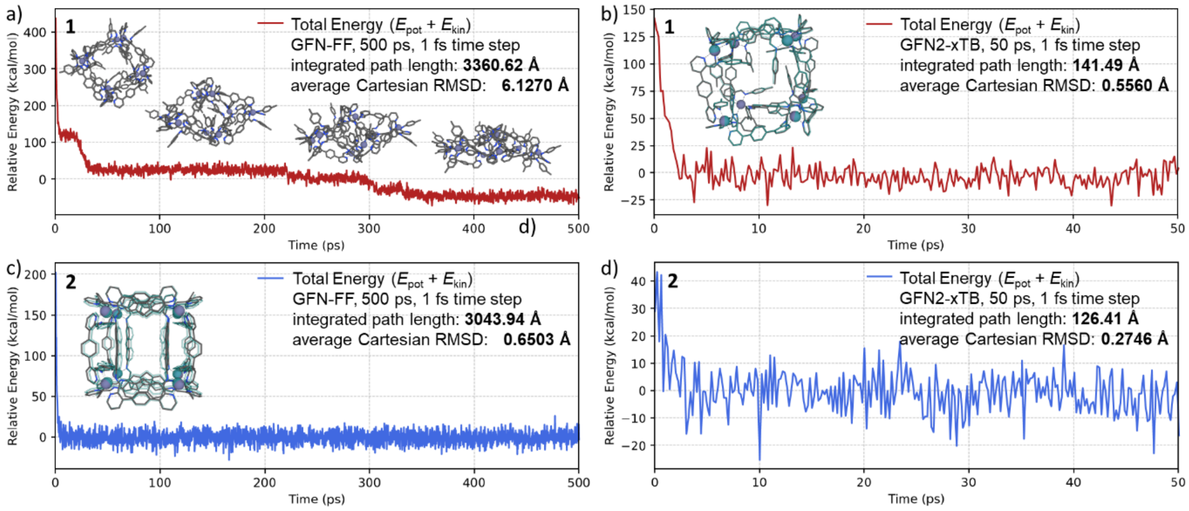

*Figure S82: Trajectory profiles for a) **1** at GFN-FF level of theory, b) **1** at GFN2-xTB level of theory, c) **2** at GFN-FF level of theory, and d) **2** at GFN2-xTB level of theory. All plots include values for the respective integrated path length and average Cartesian RMSD with respect to the starting point. Figure a) includes example snapshots corresponding to the discernible*

"plateaus" in the MD profile. Figures b) and c) include an overlay between the starting geometry and the structure averaged over all snapshots.

Results are provided in Table 3 and Figure 54. Since the integrated path length depends on the overall simulation lengths, values obtained at the GFN-FF level of theory are larger by one order of magnitude compared to those of GFN2-xTB, which is consistent with the respective simulation times of 500 ps and 50 ps. At both levels of theory, the integrated path lengths for **1** are around 10% larger than those for **2**, providing out first evidence of greater rigidity for the latter system. This result is further confirmed by the average Cartesian RMSD. Surprisingly, the RMSD of **1** at GFN-FF level of theory exceeds that of **2** by approximately a factor of 10. As we can see in Figure 1a, this difference appears to be caused by a partial collapse of the cluster during the simulations. The corresponding trajectory clearly shows several "plateaux" in the total energy profile, most likely corresponding to stable local minima along the pathway. Such artifacts may be caused by the absence of solvent molecules filling the cavity in the simulations, or due to shortcomings in the force-field itself. In fact, the combination of both effects is sometimes referred to as "gas-phase collapse" and was mentioned in the original GFN-FF publication.[40] A comparison with the GFN2-xTB method and Figure 1b immediately reveals better behaviour of the semiempirical quantum mechanical treatment. Here, non-covalent interactions, in particular C-H-π interactions between neighbouring *p*-tolyl groups, seem to be stabilizing the overall framework of **1**. Consequently, and despite the observed average Cartesian RMSD of 0.556 Å, the snapshot-averaged structure is almost identical to the optimized GFN2-xTB minimum. For **2** investigated at the GFN-FF level of theory, the average RMSD of 0.6503 Å is slightly higher than the value for **1** at the GFN2-xTB level. This difference can be explained by a structural difference between the GFN2-xTB optimized input structure, and the GFN-FF level of theory (cf. Figure 1c). Geometry optimization of the system at the force-field level yields a minimum very close to the snapshot-averaged structure. On the other hand, the average RMSD of **2** at GFN2-xTB level (Figure 1d) is the lowest observed structural difference across all simulations, with a value of 0.2746 Å. Finally, the fluctuation of total energy for **2** amounts to 10.30 kcal mol^{-1} with GFN-FF, and 8.69 kcal mol^{-1} at the GFN2-xTB level, which are much smaller than the corresponding values for **1**, amounting to 45.75 kcal mol^{-1} (caused by the gas-phase collapse) and 19.57 kcal mol^{-1}, respectively. In summary, all these metrics indicate higher structural rigidity of **2** compared to **1**. Greater integrated path lengths and higher average Cartesian RMSDs per MD snapshot for **1** compared to **2**, at both the GFN-FF and GFN2-xTB level of theory, support this hypothesis. A further clear confirmation is the presence of the gas-phase collapse of **1**, which is entirely avoided for **2**.

6. Robustness Investigations of **1** and **2**

Experimental details:

Robustness investigations were performed in NMR tubes using a 0.4 mM CD_3CN solution of the respective cage. The respective compound (aniline, TBA(OTf), $Fe(NTf_2)_2$) was added, and after heating the solution for 20 h at 70°C, a ^{1}H NMR spectrum was taken. In the case of no or minor changes, further compound was added to the same solution and after heating the solution again for 20 h at 70°C, a ^{1}H NMR spectrum was taken. The number of equivalents listed below corresponds to the total quantity added to the solution. The robustness against the Lewis base DMSO was probed upon step-wise addition of a certain volume of d_6-DMSO to 0.5 mL of a 0.4 mM CD_3CN solution of the respective cage.

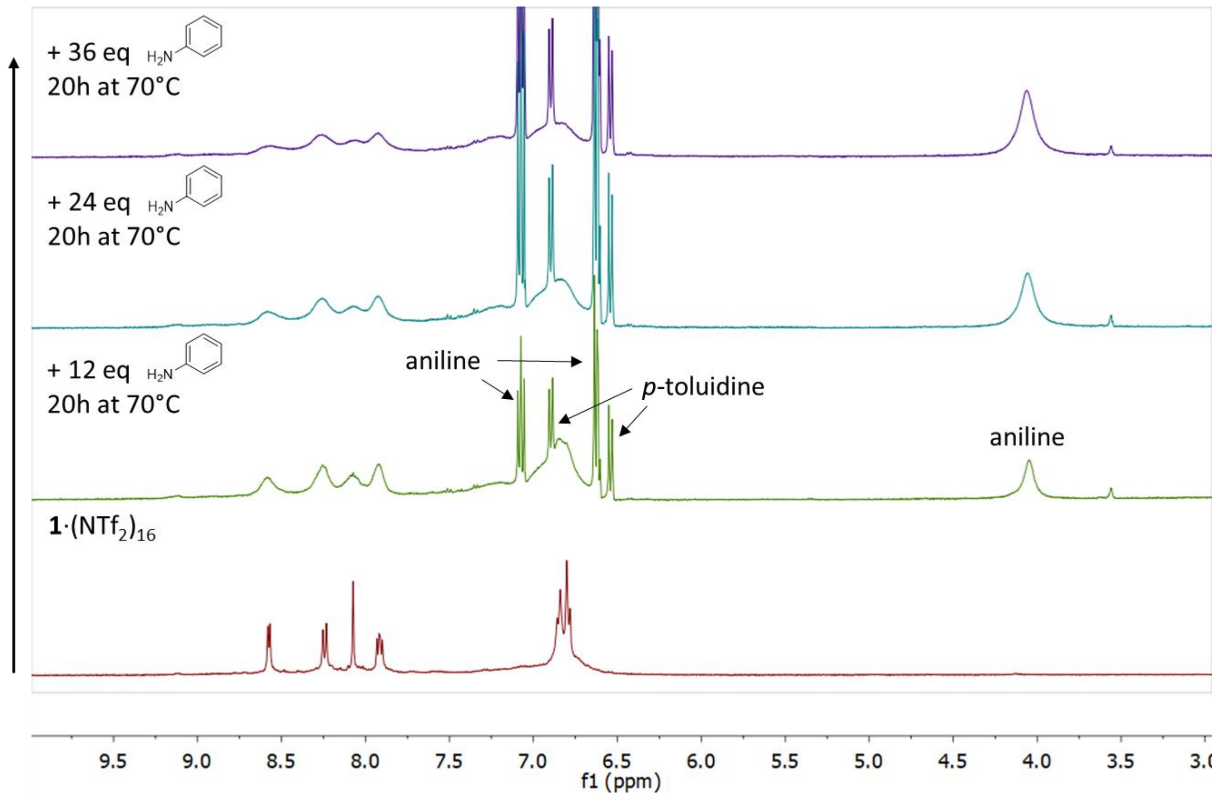

*Figure S83: ^{1}H NMR spectra of titration experiment of **1**·(NTf_2)$_{16}$ with aniline (400 MHz, CD_3CN, 298 K). The number of equivalents corresponds to the total equivalent quantity added to the solution. Broadening of the NMR signal assigned to **1**, which indicate decomposition of cage **1**, was observed upon addition of 12 equiv aniline.*

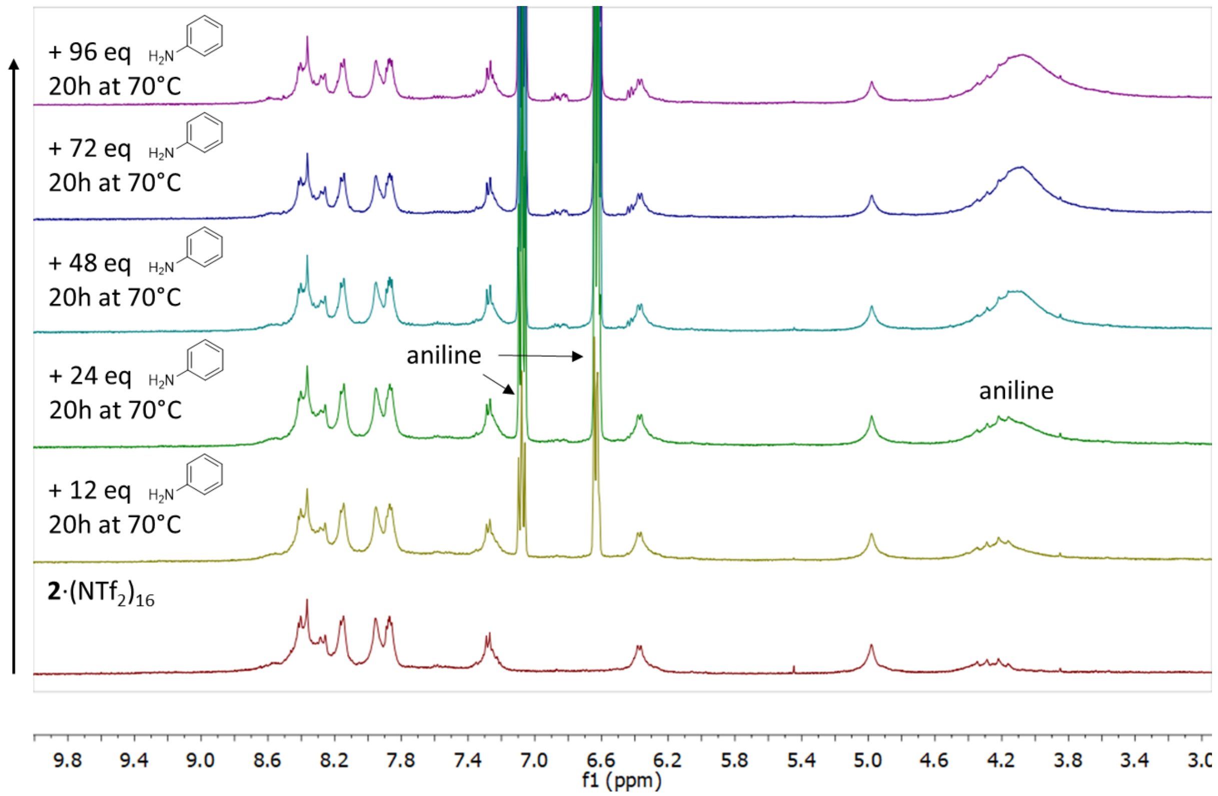

*Figure S84: ^{1}H NMR spectra of titration experiment of **2**·(NTf_2)$_{16}$ with aniline (400 MHz, CD_3CN, 298 K). The number of equivalents corresponds to the total equivalent quantity added to the solution. No decomposition of cage **2** was observed even upon addition of 96 equiv aniline.*

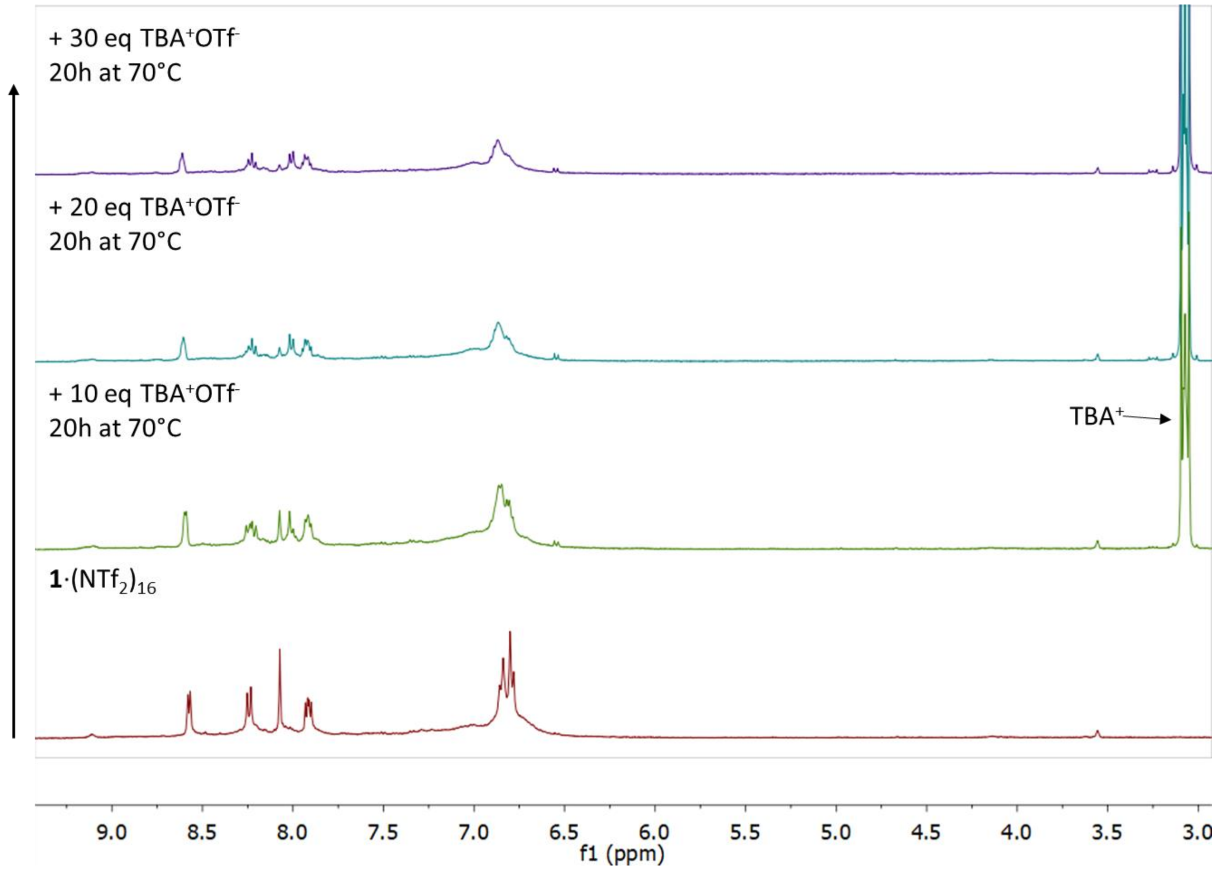

*Figure S85: ^{1}H NMR spectra of titration experiment of **1**·(NTf$_2$)$_{16}$ with TBA$^+$OTf$^-$ (400 MHz, CD_3CN, 298 K). The number of equivalents corresponds to the total equivalent quantity added to the solution. Broadening of the NMR signal assigned to **1**, which indicate decomposition of cage **1**, was observed upon addition of 20 equiv TBA$^+$OTf$^-$.*

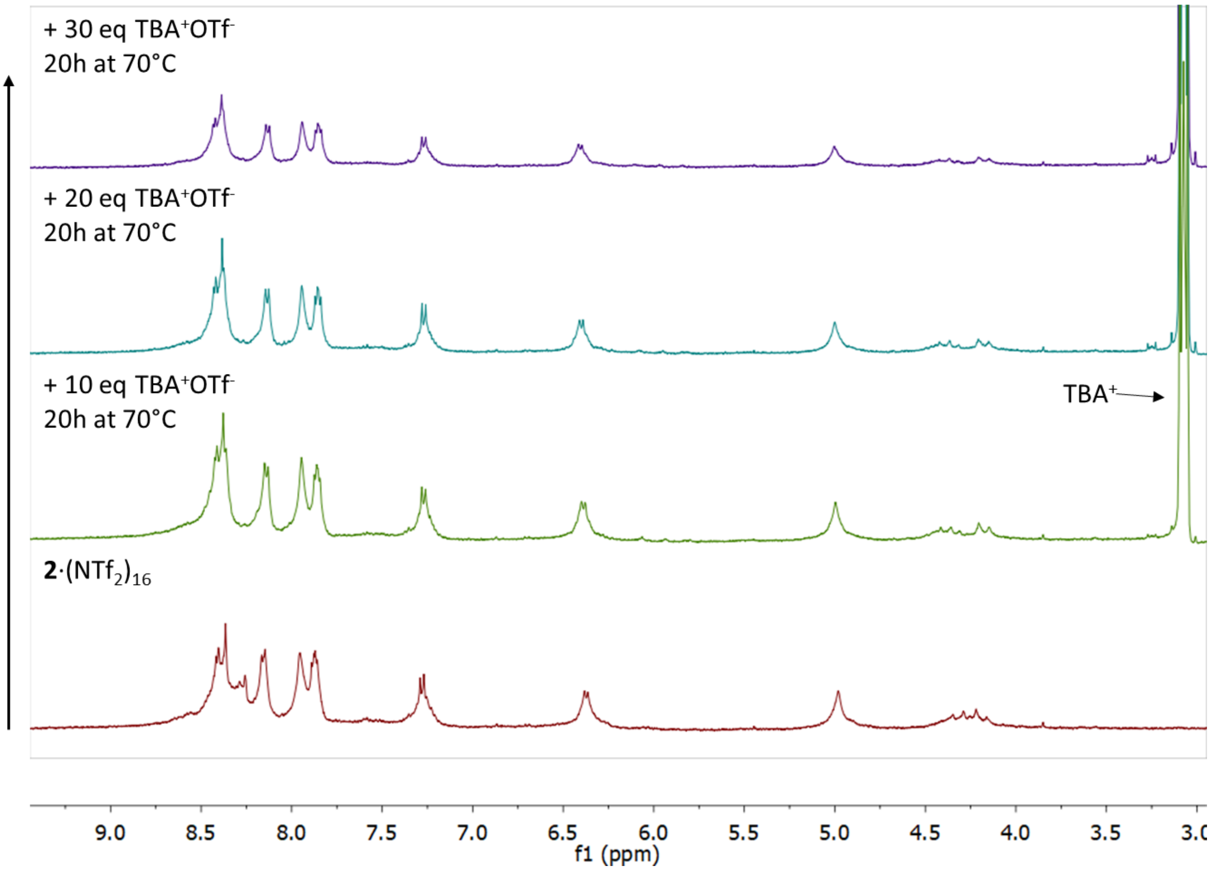

*Figure S86: ^{1}H NMR spectra of titration experiment of **2**·(NTf$_2$)$_{16}$ with TBA$^+$OTf$^-$ (400 MHz, CD$_3$CN, 298 K). The number of equivalents corresponds to the total equivalent quantity added to the solution. No decomposition of cage **2** was observed even upon addition of 30 equiv TBA$^+$OTf$^-$.*

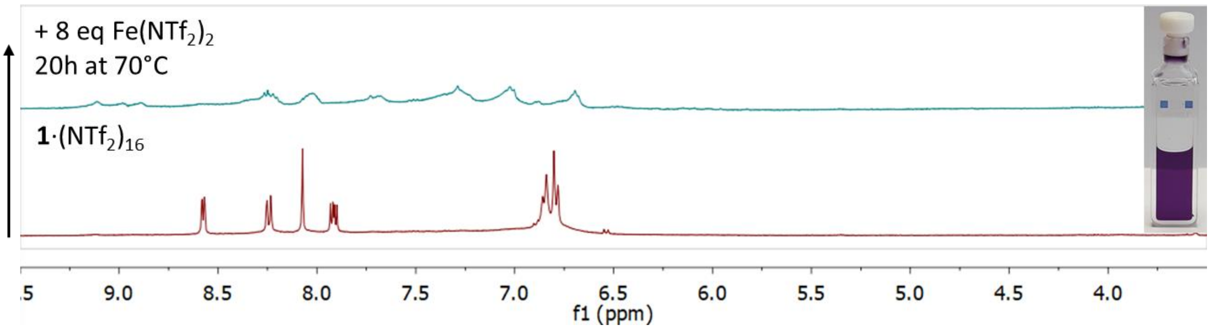

*Figure S87: ^{1}H NMR spectra of **1**·(NTf$_2$)$_{16}$ upon addition of Fe(NTf$_2$)$_2$ (400 MHz, CD$_3$CN, 298 K). The number of equivalents corresponds to the total equivalent quantity added to the solution. Broadening of the NMR signal assigned to **1**, which indicate decomposition of cage **1**, was observed upon addition of 8 equiv Fe(NTf$_2$)$_2$. The photo shows the intense purple color of the solution, indicating the formation of a Fe(II) low-spin species.*

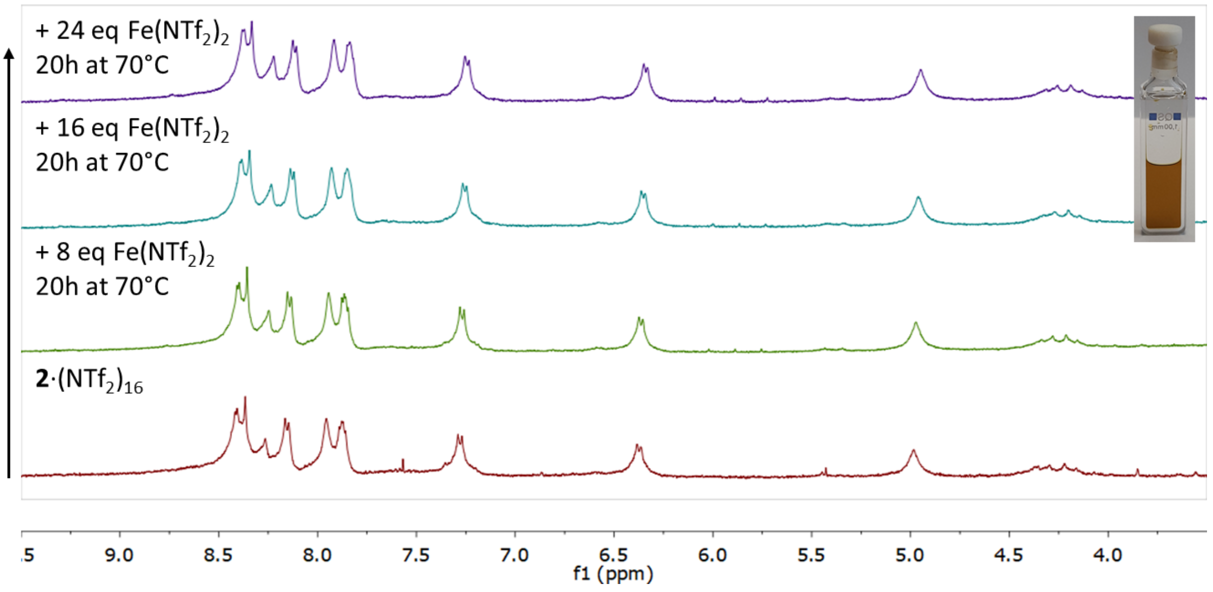

*Figure S88: ^{1}H NMR spectra of **2**·(NTf$_2$)$_{16}$ upon addition of Fe(NTf$_2$)$_2$ (400 MHz, CD$_3$CN, 298 K). The number of equivalents corresponds to the total equivalent quantity added to the solution. No decomposition of cage **2** was observed even upon addition of 24 equiv Fe(NTf$_2$)$_2$.*

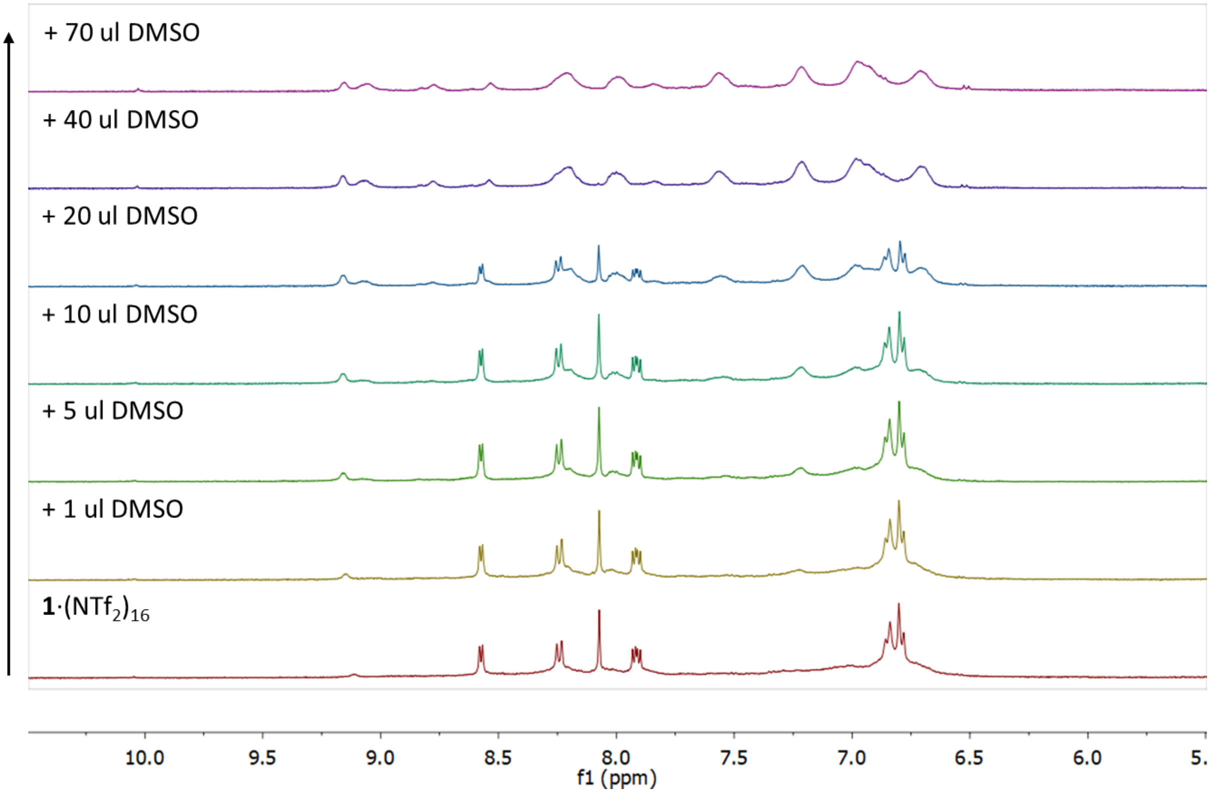

*Figure S89: ^{1}H NMR spectra of titration experiment of **1**·(NTf$_2$)$_{16}$ with d$_6$-DMSO (400 MHz, CD$_3$CN, 298 K). The volume corresponds to the total DMSO volume added to the solution. Full decomposition was observed upon addition of 40 µL d$_6$-DMSO to 0.5 mL of a 0.4 mM solution of **1**·(NTf$_2$)$_{16}$.*

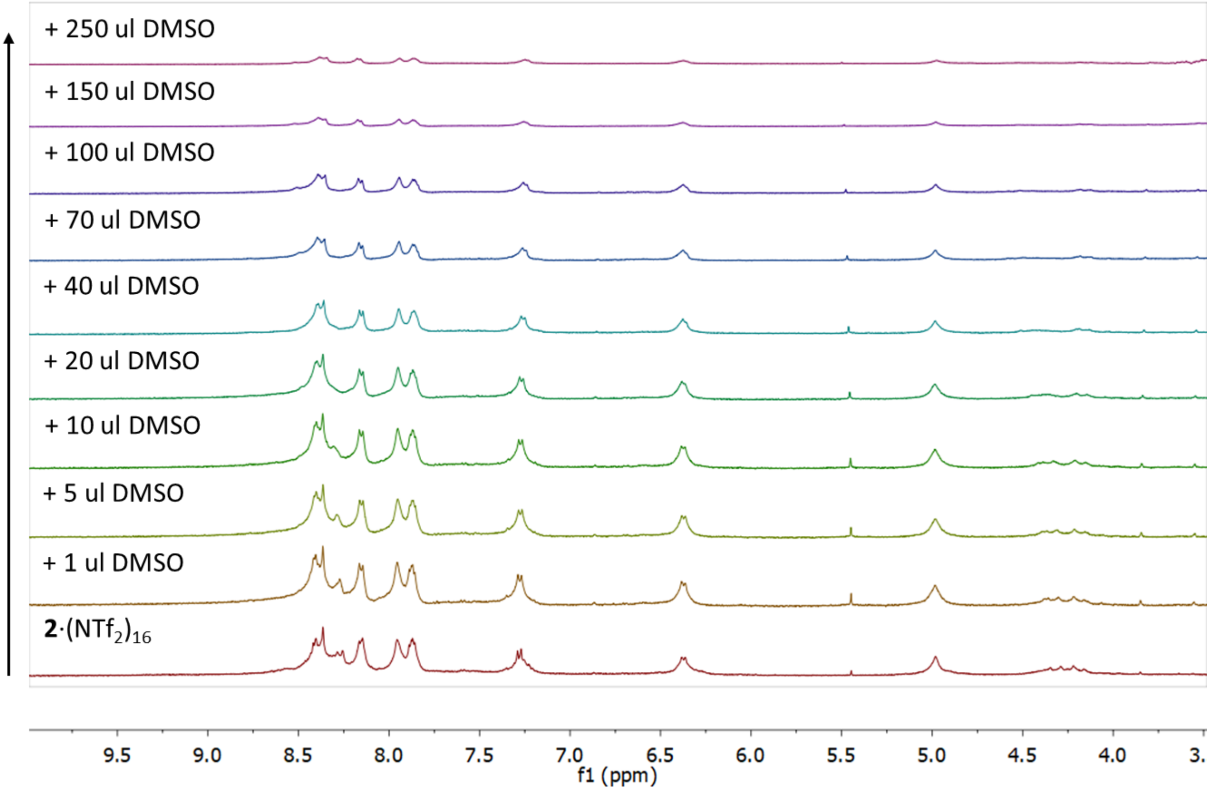

*Figure S90: ^{1}H NMR spectra of titration experiment of **2**·(NTf$_2$)$_{16}$ with d$_6$-DMSO (400 MHz, CD$_3$CN, 298 K). The volume corresponds to the total DMSO volume added to the solution. No decomposition was observed even upon addition of 250 µL d$_6$-DMSO to 0.5 mL of a 0.4 mM solution of **2**·(NTf$_2$)$_{16}$.*

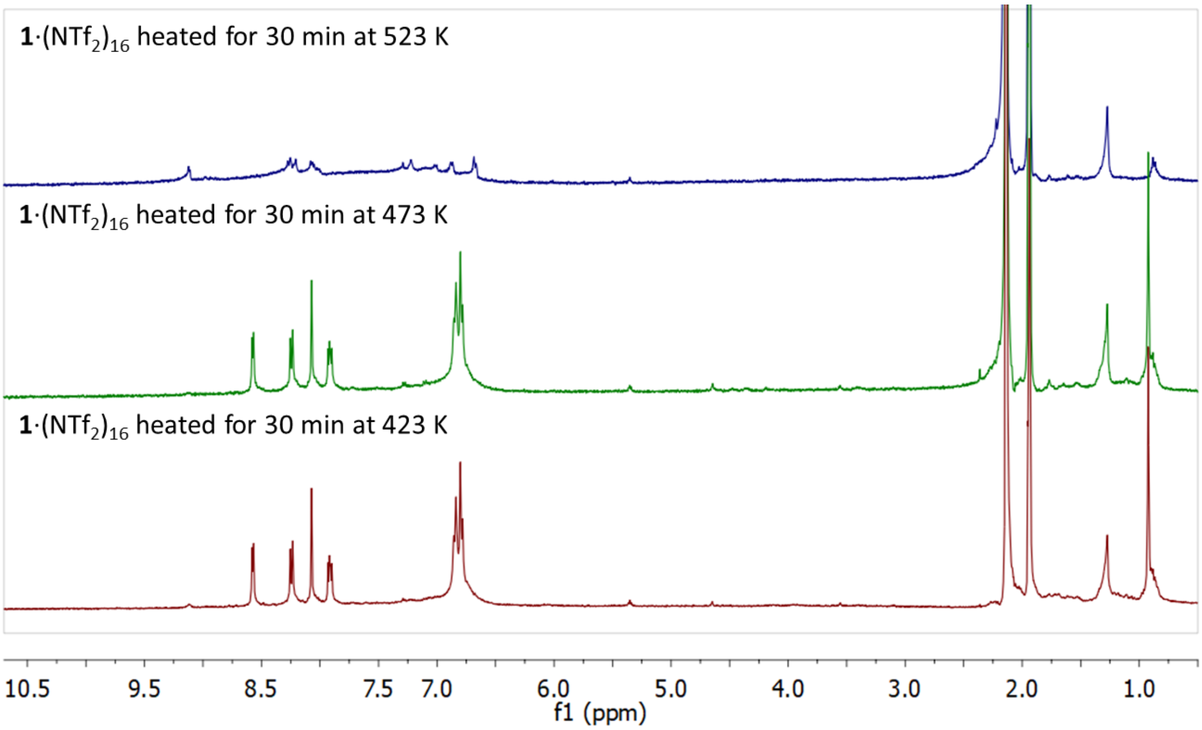

*Figure S91: ^{1}H NMR spectrum of **1**·(NTf$_2$)$_{16}$ upon heating for 30 min in the solid state at various temperatures (500 MHz, CD$_3$CN, 298 K). While the cage remained intact upon heating at 423 K and 473 K for 30 min in the solid state, complete cage decomposition was observed upon heating at 523 K for 30 min.*

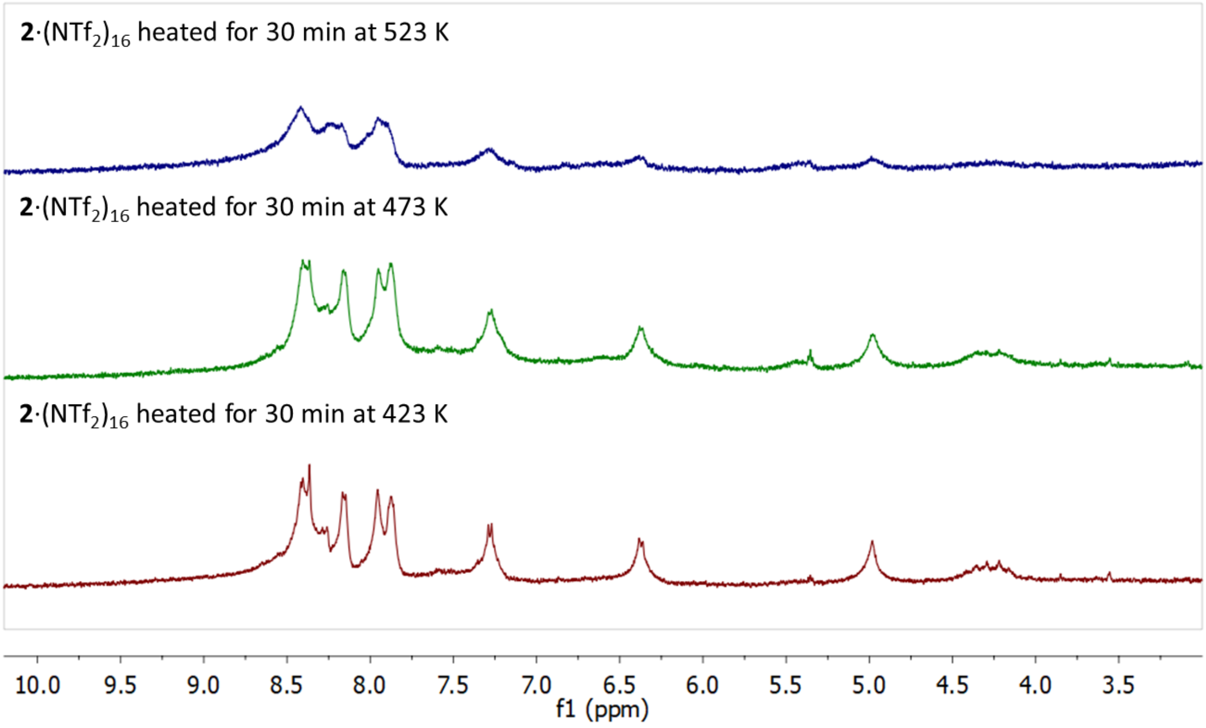

*Figure S92: ^{1}H NMR spectrum of **2**·(NTf$_2$)$_{16}$ upon heating for 30 min in the solid state at various temperatures (500 MHz, CD$_3$CN, 298 K). While the cage remained intact upon heating at 423 K and 473 K for 30 min in the solid state, a broadening of the signals was observed upon heating at 523 K for 30 min.*

7. Host-Guest Experiments

Determination of cavity void:

To determine the available void space of **1**, **2**, **3**, and **4**, MoloVol[41] calculations based on the GFN2-xTB optimized structures were performed. The cavity volumes of the three cages were calculated as 354 Å^3, x, and z, respectively. Calculations were performed on MoloVol 1.0.0 with the parameters reported below and default element radii:
1: Probe mode: one probe, Probe radius: 5.5 Å, Grid resolution: 0.2 Å, Optimization depth: 4
2: Probe mode: one probe, Probe radius: 4.2 Å, Grid resolution: 0.2 Å, Optimization depth: 4
3: Probe mode: one probe, Probe radius: 4.6 Å, Grid resolution: 0.2 Å, Optimization depth: 4
4: Probe mode: one probe, Probe radius: 7.1 Å, Grid resolution: 0.3 Å, Optimization depth: 4

Experimental details:

NMR host-guest experiments were performed in NMR tubes using a 0.4 mM CD$_3$CN solution of the respective cage. 10 equiv of the respective guest compound were added, the NMR tube shaken for 2 min and the spectrum taken after 20 min. Measuring all samples again after 20 h revealed no further shifts of the NMR signals.

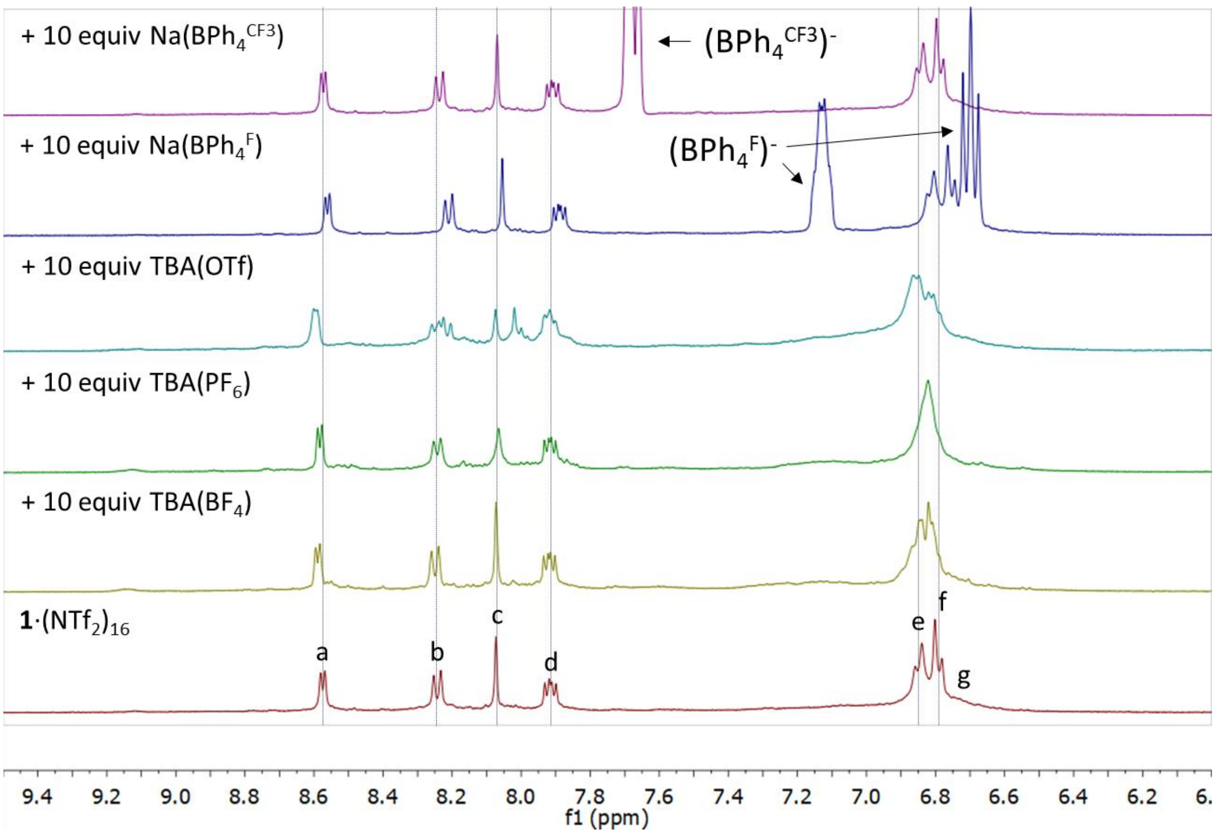

*Figure S93: Stack plot showing the changes observed in the ^{1}H NMR spectrum of **1**·$(NTf_2)_{16}$, upon the addition of 10 equiv of various anionic guest molecules (BPh_4^{F-} = tetrakis(4-fluorophenyl)borate, BPh_4^{CF3-} = tetrakis[3,5-bis(trifluoromethyl)phenyl]-borate).*

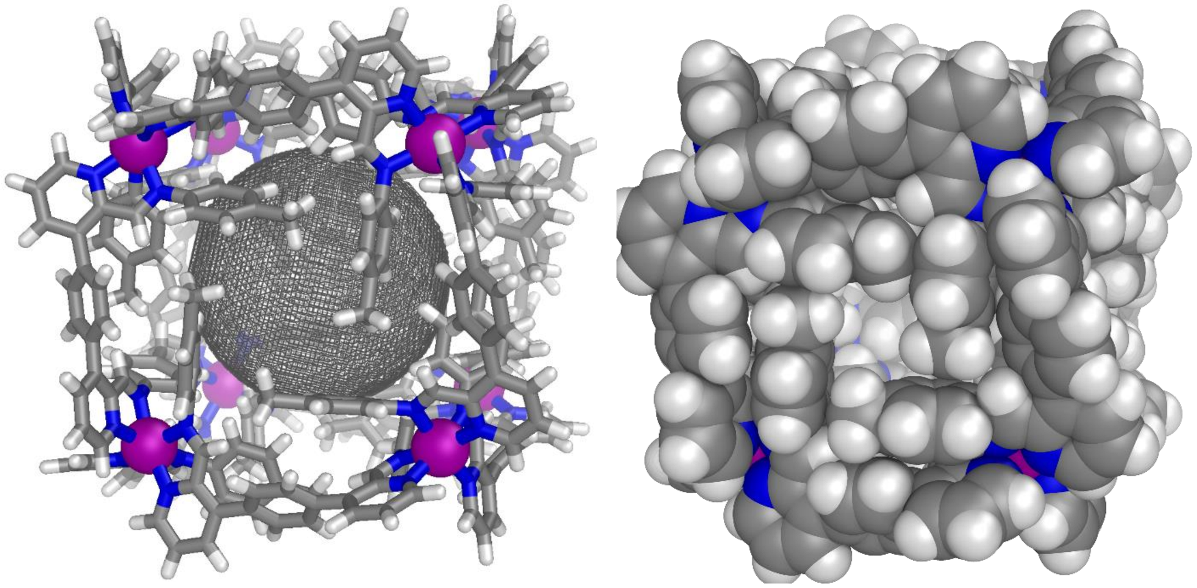

*Figure S94: Model of **1** based GFN2-xTB calculations showing the MoloVol[41] calculated cavity of 698 Å^3 in grey (left) and as a space-filling model (right). Calculations were performed without inclusion of counteranions or solvent molecules.*

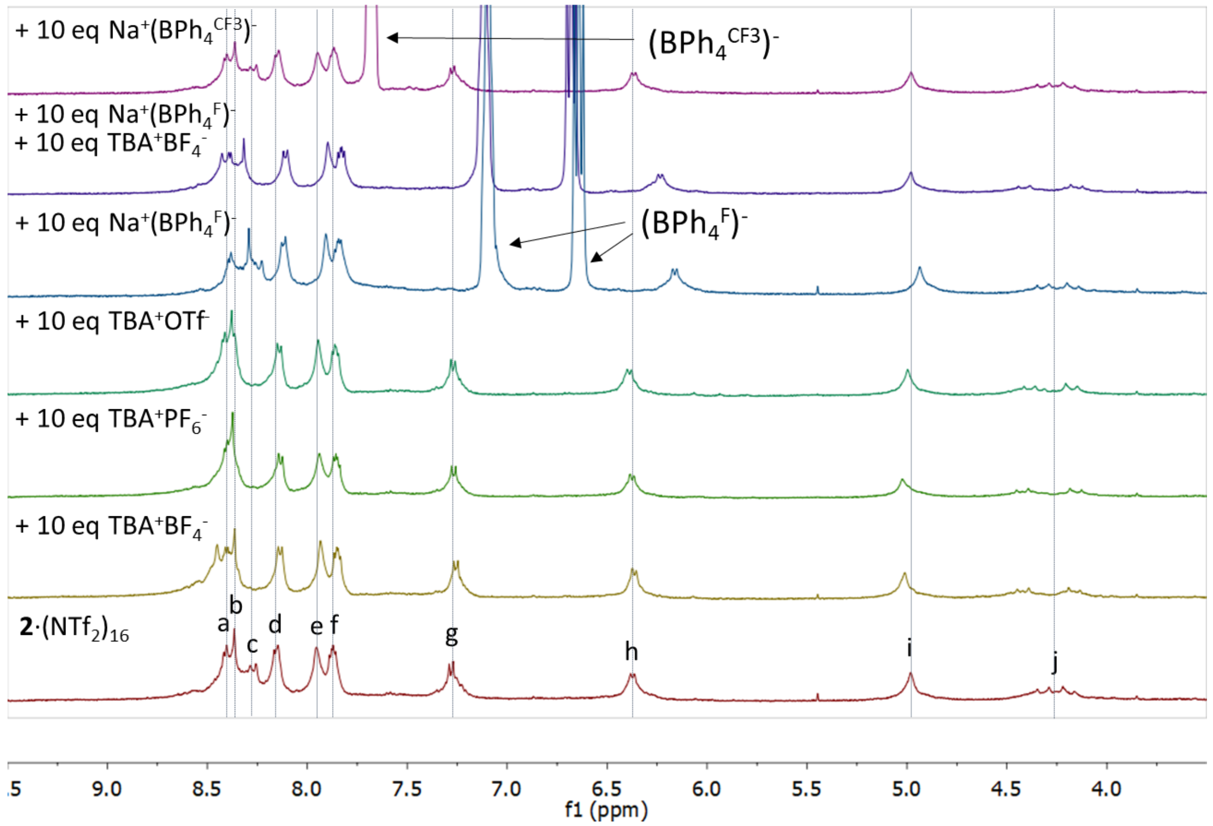

*Figure S95: Stack plot showing the changes observed in the ^{1}H NMR spectrum of **2**·$(NTf_2)_{16}$, upon the addition of 10 equiv of various anionic guest molecules (BPh_4^{F-} = tetrakis(4-fluorophenyl)borate, BPh_4^{CF3-} = tetrakis[3,5-bis(trifluoromethyl)phenyl]-borate).*

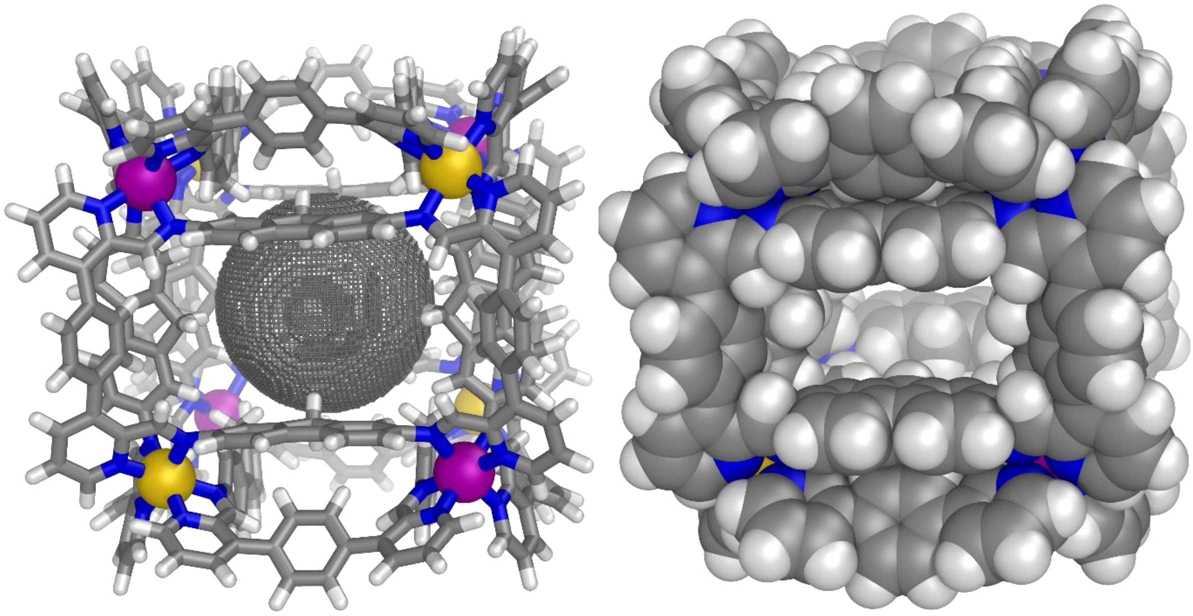

*Figure S96: Model of **2** based GFN2-xTB calculations showing the MoloVol[41] calculated cavity of 354 Å³ in grey (left) and as a space-filling model (right). Calculations were performed without inclusion of counteranions or solvent molecules.*

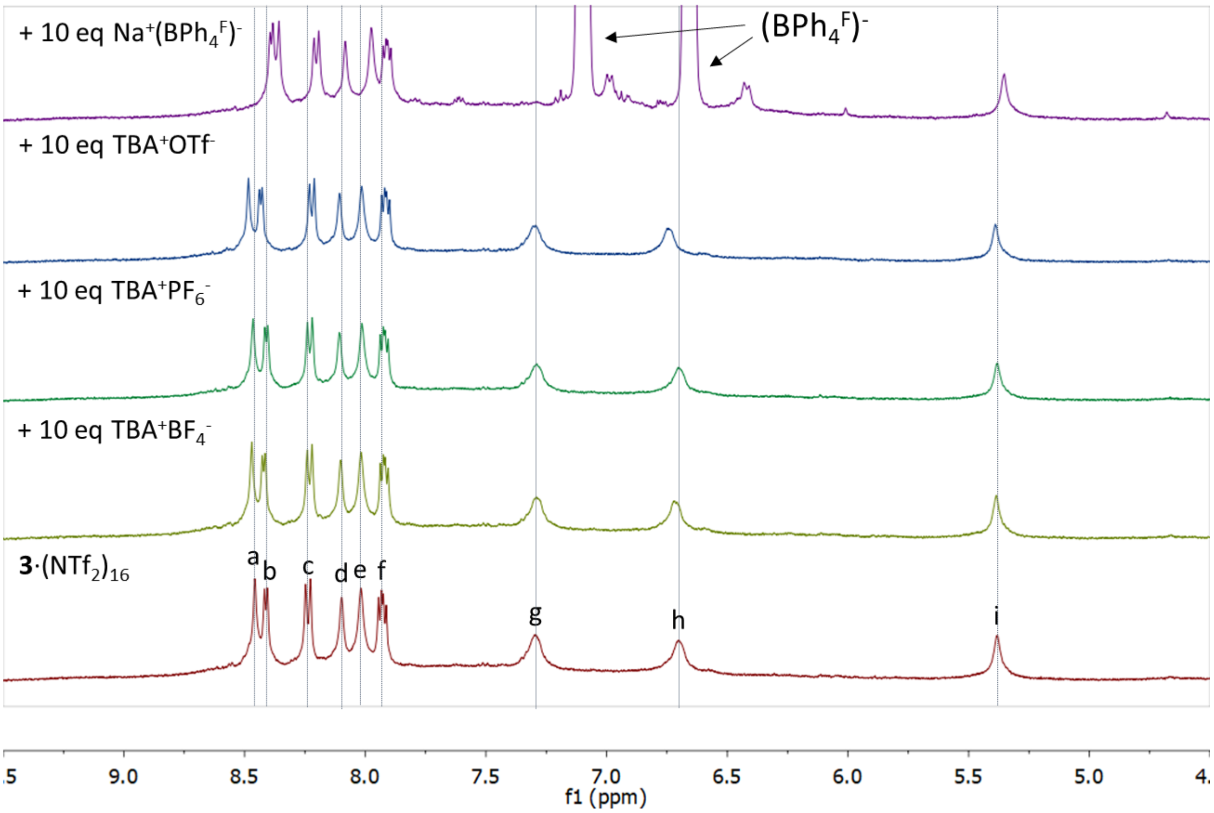

Figure S97: Stack plot showing the changes observed in the ^{1}H NMR spectrum of ***3****·(NTf₂)₁₆, upon the addition of 10 equiv of various anionic guest molecules (BPh_4^{F-} = tetrakis(4-fluorophenyl)borate, BPh_4^{CF3-} = tetrakis[3,5-bis(trifluoromethyl)phenyl]-borate).*

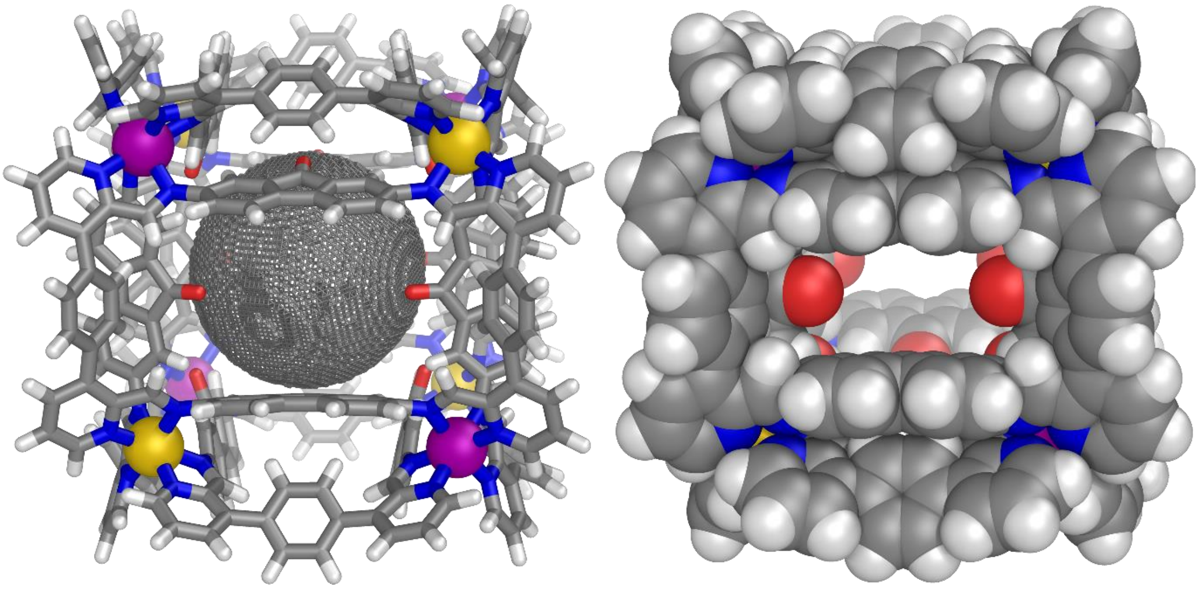

Figure S98: Model of ***3*** *based GFN2-xTB calculations showing the MoloVol[41]-calculated cavity of 443 Å³ in grey (left) and as a space-filling model (right). Calculations were performed without inclusion of counteranions or solvent molecules.*

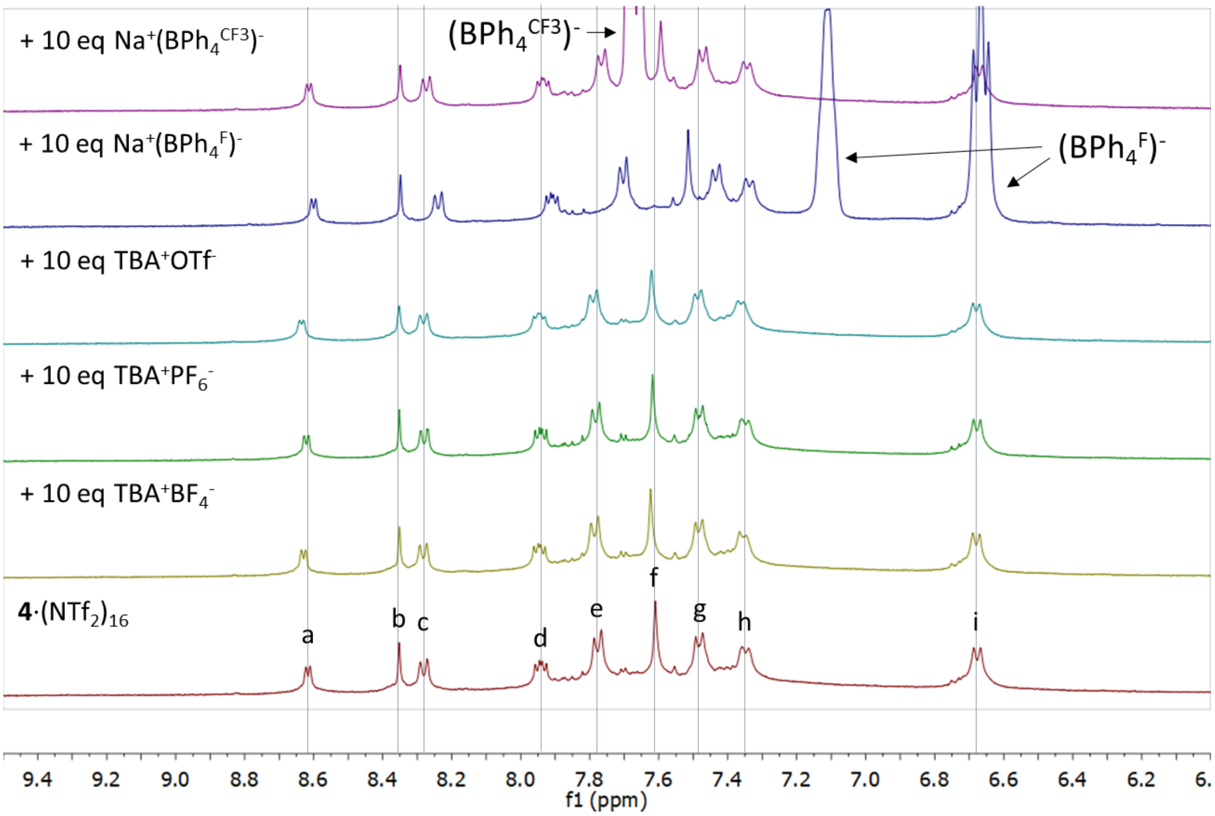

*Figure S99: Stack plot showing the changes observed in the ^{1}H NMR spectrum of **4**·(NTf$_2$)$_{16}$, upon the addition of 10 equiv of various anionic guest molecules (BPh$_4^{F-}$ = tetrakis(4-fluorophenyl)borate, BPh$_4^{CF3-}$ = tetrakis[3,5-bis(trifluoromethyl)phenyl]-borate).*

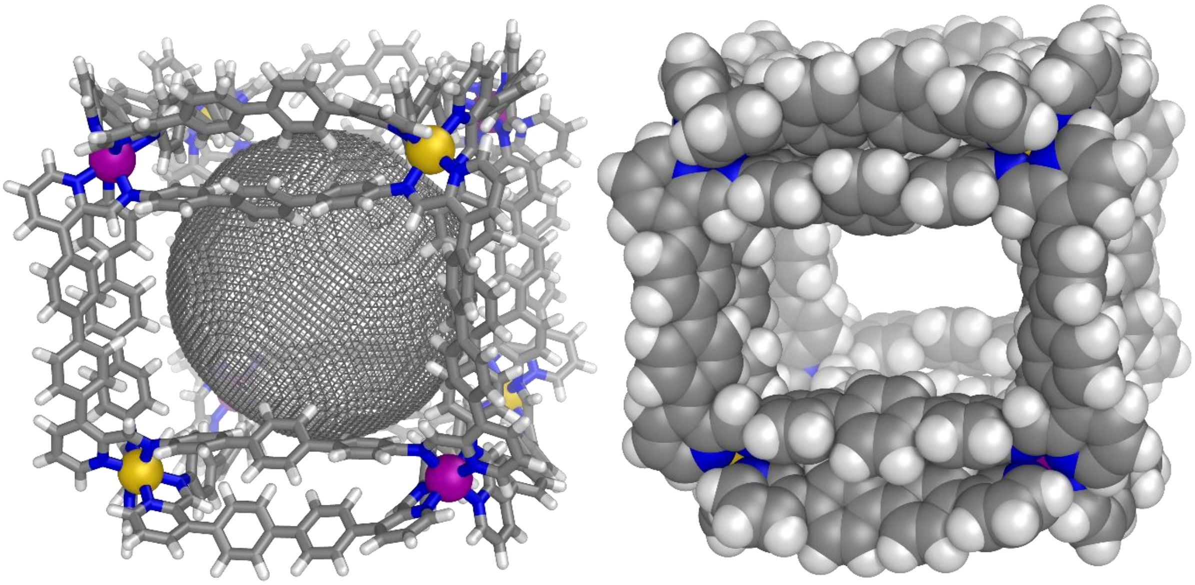

*Figure S100: Model of **4** based GFN2-xTB calculations showing the MoloVol[41]-calculated cavity of 2188 Å^3 in grey (left) and as a space-filling model (right). Calculations were performed without inclusion of counteranions or solvent molecules.*

8. Literature

(1) Rodríguez, J. G.; Tejedor, J. L.; La Parra, T.; Díaz, C. Synthesis of Conjugated 2,7-Bis(Trimethylsilylethynyl)-(Phenylethynyl)$_n$Fluoren-9-One and 9-(p-Methoxyphenyl)-9-Methyl Derivatives: Optical Properties. *Tetrahedron* **2006**, *62* (14), 3355–3361. https://doi.org/10.1016/j.tet.2006.01.058.

(2) Gilmartin, P.; Vu, C.; Rotella, M.; Kaur, J.; Kozlowski, M. Edge-Decorated Polycyclic Aromatic Hydrocarbons by an Oxidative Coupling Approach. *Chem. Eur. J.* **2023**, *29* (10), e202203405. https://doi.org/10.1002/chem.202203405.
(3) Ma, S.; Smulders, M. M. J.; Hristova, Y. R.; Clegg, J. K.; Ronson, T. K.; Zarra, S.; Nitschke, J. R. Chain-Reaction Anion Exchange between Metal–Organic Cages. *J. Am. Chem. Soc.* **2013**, *135* (15), 5678–5684. https://doi.org/10.1021/ja311882h.
(4) Browne, C.; Brenet, S.; Clegg, J. K.; Nitschke, J. R. Solvent-Dependent Host–Guest Chemistry of an Fe_8L_{12} Cubic Capsule. *Angew. Chem. Int. Ed.* **2013**, *52* (7), 1944–1948. https://doi.org/10.1002/anie.201208740.
(5) Allan, D.; Nowell, H.; Barnett, S.; Warren, M.; Wilcox, A.; Christensen, J.; Saunders, L.; Peach, A.; Hooper, M.; Zaja, L.; Patel, S.; Cahill, L.; Marshall, R.; Trimnell, S.; Foster, A.; Bates, T.; Lay, S.; Williams, M.; Hathaway, P.; Winter, G.; Gerstel, M.; Wooley, R. A Novel Dual Air-Bearing Fixed-χ Diffractometer for Small-Molecule Single-Crystal X-Ray Diffraction on Beamline I19 at Diamond Light Source. *Crystals* **2017**, *7* (11), 336. https://doi.org/10.3390/cryst7110336.
(6) Evans, P. Scaling and Assessment of Data Quality. *Acta Crystallogr. D Biol. Crystallogr.* **2006**, *62* (1), 72–82. https://doi.org/10.1107/S0907444905036693.
(7) Winter, G. *Xia2*: An Expert System for Macromolecular Crystallography Data Reduction. *J. Appl. Crystallogr.* **2010**, *43* (1), 186–190. https://doi.org/10.1107/S0021889809045701.
(8) Winter, G.; Waterman, D. G.; Parkhurst, J. M.; Brewster, A. S.; Gildea, R. J.; Gerstel, M.; Fuentes-Montero, L.; Vollmar, M.; Michels-Clark, T.; Young, I. D.; Sauter, N. K.; Evans, G. *DIALS*: Implementation and Evaluation of a New Integration Package. *Acta Crystallogr. D Struct. Biol.* **2018**, *74* (2), 85–97. https://doi.org/10.1107/S2059798317017235.
(9) Farrugia, L. J. *WinGX* and *ORTEP for Windows*: An Update. *J Appl. Crystallogr.* **2012**, *45* (4), 849–854. https://doi.org/10.1107/S0021889812029111.
(10) Dolomanov, O. V.; Bourhis, L. J.; Gildea, R. J.; Howard, J. A. K.; Puschmann, H. *OLEX2*: A Complete Structure Solution, Refinement and Analysis Program. *J Appl. Crystallogr.* **2009**, *42* (2), 339–341. https://doi.org/10.1107/S0021889808042726.
(11) Evans, P. R.; Murshudov, G. N. How Good Are My Data and What Is the Resolution? *Acta Crystallogr. D Biol. Crystallogr.* **2013**, *69* (7), 1204–1214. https://doi.org/10.1107/S0907444913000061.
(12) Winn, M. D.; Ballard, C. C.; Cowtan, K. D.; Dodson, E. J.; Emsley, P.; Evans, P. R.; Keegan, R. M.; Krissinel, E. B.; Leslie, A. G. W.; McCoy, A.; McNicholas, S. J.; Murshudov, G. N.; Pannu, N. S.; Potterton, E. A.; Powell, H. R.; Read, R. J.; Vagin, A.; Wilson, K. S. Overview of the *CCP* 4 Suite and Current Developments. *Acta Crystallogr. D Biol. Crystallogr.* **2011**, *67* (4), 235–242. https://doi.org/10.1107/S0907444910045749.
(13) Sheldrick, G. M. *SHELXT* – Integrated Space-Group and Crystal-Structure Determination. *Acta Crystallogr. A Found Adv.* **2015**, *71* (1), 3–8. https://doi.org/10.1107/S2053273314026370.
(14) Sheldrick, G. M. Crystal Structure Refinement with *SHELXL*. *Acta Crystallogr. C Struct. Chem.* **2015**, *71* (1), 3–8. https://doi.org/10.1107/S2053229614024218.
(15) Bricogne, G.; Blanc, E.; Brandle, M.; Flensburg, C.; Keller, P.; Paciorek, W.; Roversi, P.; Sharff, A.; Smart, O. S.; Vonrhein, C.; Womack, T. O., (last). 2011.
(16) Smart, O. S.; Womack, T. O. 2014.
(17) van der Sluis, P.; Spek, A. L. BYPASS: An Effective Method for the Refinement of Crystal Structures Containing Disordered Solvent Regions. *Acta Crystallogr. A Found Crystallogr.* **1990**, *46* (3), 194–201. https://doi.org/10.1107/S0108767389011189.
(18) Spek, A. L. 2008.
(19) OPTIM: A Program for Geometry Optimisation and Pathway Calculations. http://www-wales.ch.cam.ac.uk/software.html.
(20) Wesołowski, P. A.; Wales, D. J.; Pracht, P. Multilevel Framework for Analysis of Protein Folding Involving Disulfide Bond Formation. *J. Phys. Chem. B* **2024**, *128* (13), 3145–3156. https://doi.org/10.1021/acs.jpcb.4c00104.

(21) Bannwarth, C.; Caldeweyher, E.; Ehlert, S.; Hansen, A.; Pracht, P.; Seibert, J.; Spicher, S.; Grimme, S. Extended TIGHT-BINDING Quantum Chemistry Methods. *WIREs Comput. Mol. Sci.* **2021**, *11* (2), e1493. https://doi.org/10.1002/wcms.1493.
(22) Bursch, M.; Neugebauer, H.; Grimme, S. Structure Optimisation of Large Transition-Metal Complexes with Extended Tight-Binding Methods. *Angew. Chem. Int. Ed.* **2019**, *58* (32), 11078–11087. https://doi.org/10.1002/anie.201904021.
(23) Bannwarth, C.; Ehlert, S.; Grimme, S. GFN2-xTB—An Accurate and Broadly Parametrized Self-Consistent Tight-Binding Quantum Chemical Method with Multipole Electrostatics and Density-Dependent Dispersion Contributions. *J. Chem. Theory Comput.* **2019**, *15* (3), 1652–1671. https://doi.org/10.1021/acs.jctc.8b01176.
(24) Pracht, P.; Bohle, F.; Grimme, S. Automated Exploration of the Low-Energy Chemical Space with Fast Quantum Chemical Methods. *Phys. Chem. Chem. Phys.* **2020**, *22* (14), 7169–7192. https://doi.org/10.1039/C9CP06869D.
(25) Pracht, P.; Grimme, S.; Bannwarth, C.; Bohle, F.; Ehlert, S.; Feldmann, G.; Gorges, J.; Müller, M.; Neudecker, T.; Plett, C.; Spicher, S.; Steinbach, P.; Wesołowski, P. A.; Zeller, F. CREST—A Program for the Exploration of Low-Energy Molecular Chemical Space. *J. Chem. Phys.* **2024**, *160* (11), 114110. https://doi.org/10.1063/5.0197592.
(26) Grimme, S. Supramolecular Binding Thermodynamics by Dispersion-Corrected Density Functional Theory. *Chem. Eur. J.* **2012**, *18* (32), 9955–9964. https://doi.org/10.1002/chem.201200497.
(27) Pracht, P.; Grimme, S. Calculation of Absolute Molecular Entropies and Heat Capacities Made Simple. *Chem. Sci.* **2021**, *12* (19), 6551–6568. https://doi.org/10.1039/D1SC00621E.
(28) Neese, F.; Wennmohs, F.; Becker, U.; Riplinger, C. The ORCA Quantum Chemistry Program Package. *J. Chem. Phys.* **2020**, *152* (22), 224108. https://doi.org/10.1063/5.0004608.
(29) Bursch, M.; Hansen, A.; Pracht, P.; Kohn, J. T.; Grimme, S. Theoretical Study on Conformational Energies of Transition Metal Complexes. *Phys. Chem. Chem. Phys.* **2021**, *23* (1), 287–299. https://doi.org/10.1039/D0CP04696E.
(30) Kruse, H.; Grimme, S. A Geometrical Correction for the Inter- and Intra-Molecular Basis Set Superposition Error in Hartree-Fock and Density Functional Theory Calculations for Large Systems. *J. Chem. Phys.* **2012**, *136* (15), 154101. https://doi.org/10.1063/1.3700154.
(31) Caldeweyher, E.; Bannwarth, C.; Grimme, S. Extension of the D3 Dispersion Coefficient Model. *J. Chem. Phys.* **2017**, *147* (3), 034112. https://doi.org/10.1063/1.4993215.
(32) Tarzia, A.; Jelfs, K. E. Unlocking the Computational Design of Metal–Organic Cages. *Chem. Commun.* **2022**, *58* (23), 3717–3730. https://doi.org/10.1039/D2CC00532H.
(33) Caldeweyher, E.; Ehlert, S.; Hansen, A.; Neugebauer, H.; Spicher, S.; Bannwarth, C.; Grimme, S. A Generally Applicable Atomic-Charge Dependent London Dispersion Correction. *J. Chem. Phys.* **2019**, *150* (15), 154122. https://doi.org/10.1063/1.5090222.
(34) Grimme, S.; Hansen, A.; Ehlert, S.; Mewes, J.-M. r2SCAN-3c: A “Swiss Army Knife” Composite Electronic-Structure Method. *J. Chem. Phys.* **2021**, *154* (6), 064103. https://doi.org/10.1063/5.0040021.
(35) Ree, N.; Göller, A. H.; Jensen, J. H. What the Heck? – Automated Regioselectivity Calculations of Palladium-Catalyzed Heck Reactions Using Quantum Chemistry. *ACS Omega* **2022**, *7* (49), 45617–45623. https://doi.org/10.1021/acsomega.2c06378.
(36) Hermans, S. M. A.; Pfleger, C.; Nutschel, C.; Hanke, C. A.; Gohlke, H. Rigidity Theory for Biomolecules: Concepts, Software, and Applications. *WIREs Compu.t Mol. Sci.* **2017**, *7* (4), e1311. https://doi.org/10.1002/wcms.1311.
(37) Nocedal, J. Updating Quasi-Newton Matrices with Limited Storage. *Math. Comp.* **1980**, *35* (151), 773–782. https://doi.org/10.1090/S0025-5718-1980-0572855-7.
(38) Liu, D. C.; Nocedal, J. On the Limited Memory BFGS Method for Large Scale Optimization. *Math. Program.* **1989**, *45* (1–3), 503–528. https://doi.org/10.1007/BF01589116.

(39) *Software developments by D.J. Wales and co-workers*. http://www-wales.ch.cam.ac.uk/software.html.
(40) Spicher, S.; Grimme, S. Robust Atomistic Modeling of Materials, Organometallic, and Biochemical Systems. *Angew. Chem. Int. Ed.* **2020**, *59* (36), 15665–15673. https://doi.org/10.1002/anie.202004239.
(41) Maglic, J. B.; Lavendomme, R. *MoloVol*: An Easy-to-Use Program for Analyzing Cavities, Volumes and Surface Areas of Chemical Structures. *J. Appl. Crystallogr.* **2022**, *55* (4), 1033–1044. https://doi.org/10.1107/S1600576722004988.
